# Real‐world evidence for preventive effects of statins on cancer incidence: A trans‐Atlantic analysis

**DOI:** 10.1002/ctm2.726

**Published:** 2022-02-20

**Authors:** Bjoern‐O Gohlke, Fabian Zincke, Andreas Eckert, Dennis Kobelt, Saskia Preissner, Juliane Maria Liebeskind, Nikolas Gunkel, Kerstin Putzker, Joe Lewis, Sally Preissner, Benedikt Kortüm, Wolfgang Walther, Cameron Mura, Philip E. Bourne, Ulrike Stein, Robert Preissner

**Affiliations:** ^1^ Department of Information Technology Science‐IT Charité‐Universitätsmedizin Berlin Berlin Germany; ^2^ Experimental and Clinical Research Center Charité‐Universitätsmedizin Berlin Berlin Germany; ^3^ Max‐Delbrück‐Center for Molecular Medicine Berlin Germany; ^4^ Dennis Kobelt, Wolfgang Walther and Ulrike Stein German Cancer Consortium (DKTK) Heidelberg Germany; ^5^ Cancer Drug Development Group German Cancer Research Center (DKFZ) Heidelberg Germany; ^6^ Chemical Biology Core Facility European Molecular Biology Laboratory (EMBL) Heidelberg Germany; ^7^ Institute for Physiology Charité‐Universitätsmedizin Berlin Berlin Germany; ^8^ School of Data Science University of Virginia Charlottesville Virginia USA; ^9^ Department of Biomedical Engineering University of Virginia Charlottesville Virginia USA

**Keywords:** cancer, electronic health record, MACC1, real‐world evidence, statin

Dear Editor,

Cancer metastases severely limit therapeutic options: their aggressiveness and treatment resistance^1^ demand novel therapeutic targets, new/repositioned drugs, or alternative interventions. We previously discovered that lovastatin restricts colorectal cancer (CRC) growth and metastasis via transcriptional inhibition of a tumour‐promoting “metastasis‐associated in colon cancer 1″ (MACC1) gene.^2^ MACC1, identified by our group,^3^ is a known prognostic and predictive biomarker for more than 20 solid tumour entities.^4^ In our present high‐throughput drug screen with HCT116/pMACC1‐Luc cells (Figure 1A) we identified novel transcriptional MACC1 suppressors, for example, fluvastatin, as promising inhibitors. Atorvastatin and simvastatin also reduced MACC1 mRNA and protein expression in a concentration‐dependent manner (Figure 1B). We confirmed the inhibitory effects of statins in pancreatic and gastric cancer cells (Figure S1).

**FIGURE 1:**
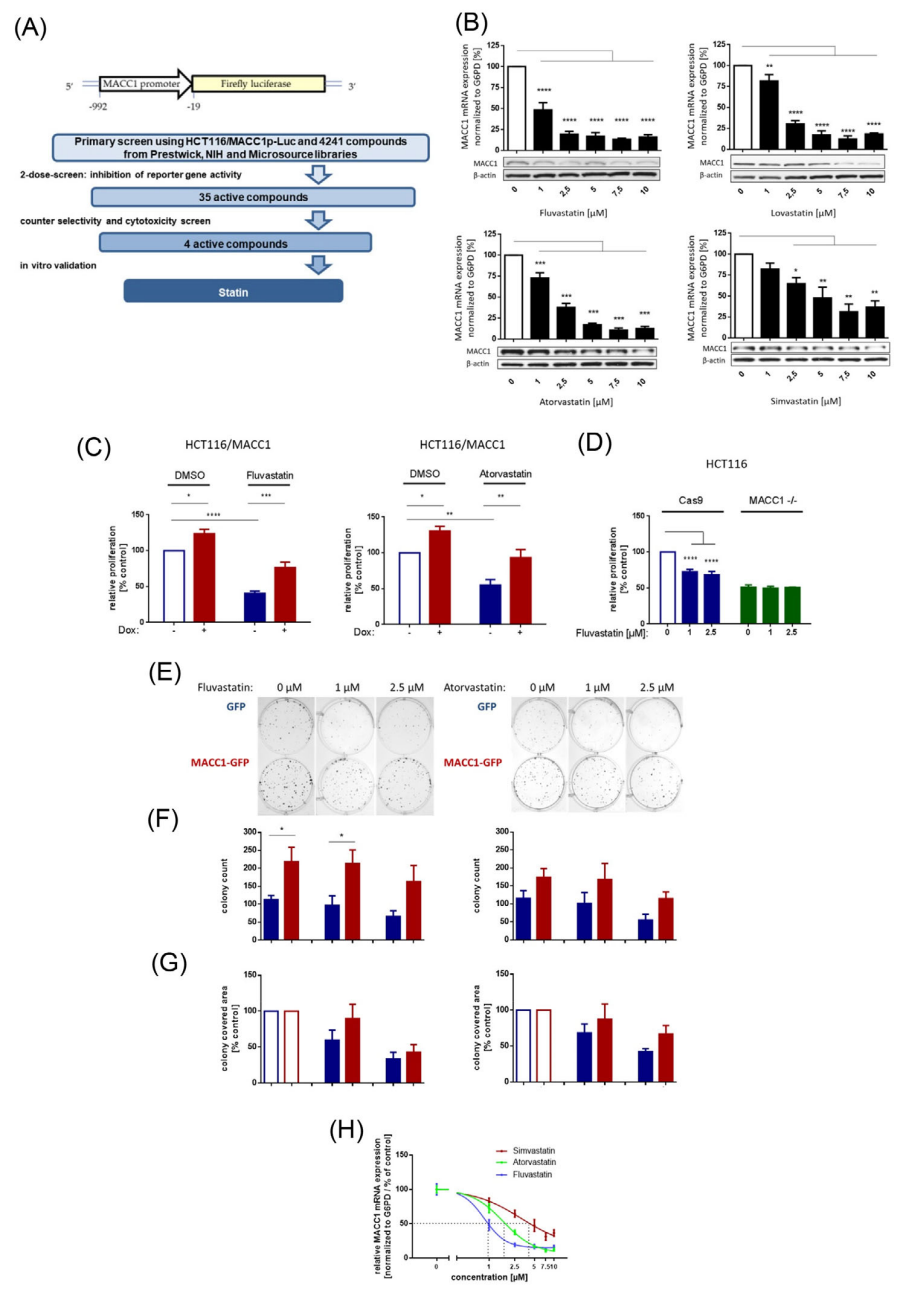
Statins reduce metastasis‐associated in colon cancer 1 (MACC1) expression and specifically inhibit MACC1‐mediated functions in vitro. This schematic presentation (A) illustrates the vector expressed in HCT116/ MACC1p‐Luc cells that were employed in our high‐throughput drug screen (HTS). The luciferase reporter gene is controlled by the MACC1 promoter (MACC1p). A tiered process revealed fluvastatin as a transcriptional inhibitor of MACC1, with a total of 4241 compounds tested. The initial two‐dose screening was followed by cytotoxicity and selectivity (with HCT116/CMVp‐Luc cells) assessment as well as in vitro validation. Dose‐dependent reduction of MACC1 messenger RNA (mRNA) and protein expression in HCT116 cells by different statins (fluvastatin, atorvastatin, lovastatin, simvastatin) is shown in (B). MACC1 mRNA levels were normalized to G6PD mRNA expression and respective treatment controls (dimethyl sulfoxide [DMSO], indicated with white bars). Results for mRNA represent the means ± standard error of the mean (SEM) of three independent experiments; for Western blot, one representative example of at least two independent experiments is shown. In the Western blot, β‐actin or vinculin served as loading controls. Significant results were determined by one‐way analysis of variance (ANOVA) and Dunnett's multiple comparison test with a 95% CI (* = *p* < .05, ** = *p* < .01, *** = *p* < .001, **** = *p* < .0001). Relative proliferation (C) was determined with the IncuCyte live imaging system for 72 h and calculated by the area under the curve (AUC), normalized to untreated controls (white bars). HCT116 cells with doxycycline‐induced MACC1 expression (+Dox) demonstrated a 30% increase in proliferation. MACC1‐induced proliferation in +Dox compared to ‐Dox cells was still observed under statin treatment, indicating a MACC1‐specific rescue of proliferative function (C); our previous findings were confirmed using HCT116/MACC1 ‐/‐. MACC1 knock‐out resulted in a >50% reduction in proliferation versus control cells (HCT116/Cas9). Control cell proliferation was decreased by fluvastatin treatment (1 and 2.5 μM), whereas HCT116/MACC1 ‐/‐ cells remained unaffected (D). Stable overexpression of MACC1‐GFP in HCT116 led to strongly augmented colony formation compared to HCT116/GFP cells. The same effect was seen under statin treatment, indicating a MACC1‐specific rescue of clonogenic function (E). Clonogenicity was quantified by the number of colonies (F) and colony covered area (G). Results represent means + SEM of at least three independent experiments, presented as total counts (F) or normalized to solvent treated controls (white bars, G). For the clonogenic assay, one representative of nine technical replicates of three independent experiments is shown. Significant results were determined by one‐way or two‐way ANOVA and Sidak's multiple comparison test with a confidence interval of 95% (* = *p* < .05, ** = *p* < .01, *** = *p* < .001, **** = *p* < .0001). Panel (H) shows the relative MACC1 mRNA expression presented as drug‐response curves for IC50 determination. Fluvastatin (IC50: 0.8457 μM) is able to reduce MACC1 mRNA expression at lower doses as atorvastatin (IC50: 1.647 μM) and simvastatin (IC50: 3.098 μM)

MACC1‐dependent proliferation and cell clone forming abilities were inhibited by statins and partly rescued by induced overexpression (Figure 1C–G). Knocking‐out MACC1 reduced proliferation (not further reducible by statin treatment). In vivo, we treated CRC xenografted SCID‐beige mice with a human equivalent statin dose, which reduced MACC1 expression, tumour burden and metastasis formation (day 24; control vs. statin treatment *p* < .0001, Figure S2 and Figure 2). Thus, at a molecular/mechanistic level, we provide experimental evidence that statins act, at least partly, by inhibiting transcription of the tumour‐promoting and metastasis‐inducing MACC1 gene.

**FIGURE 2:**
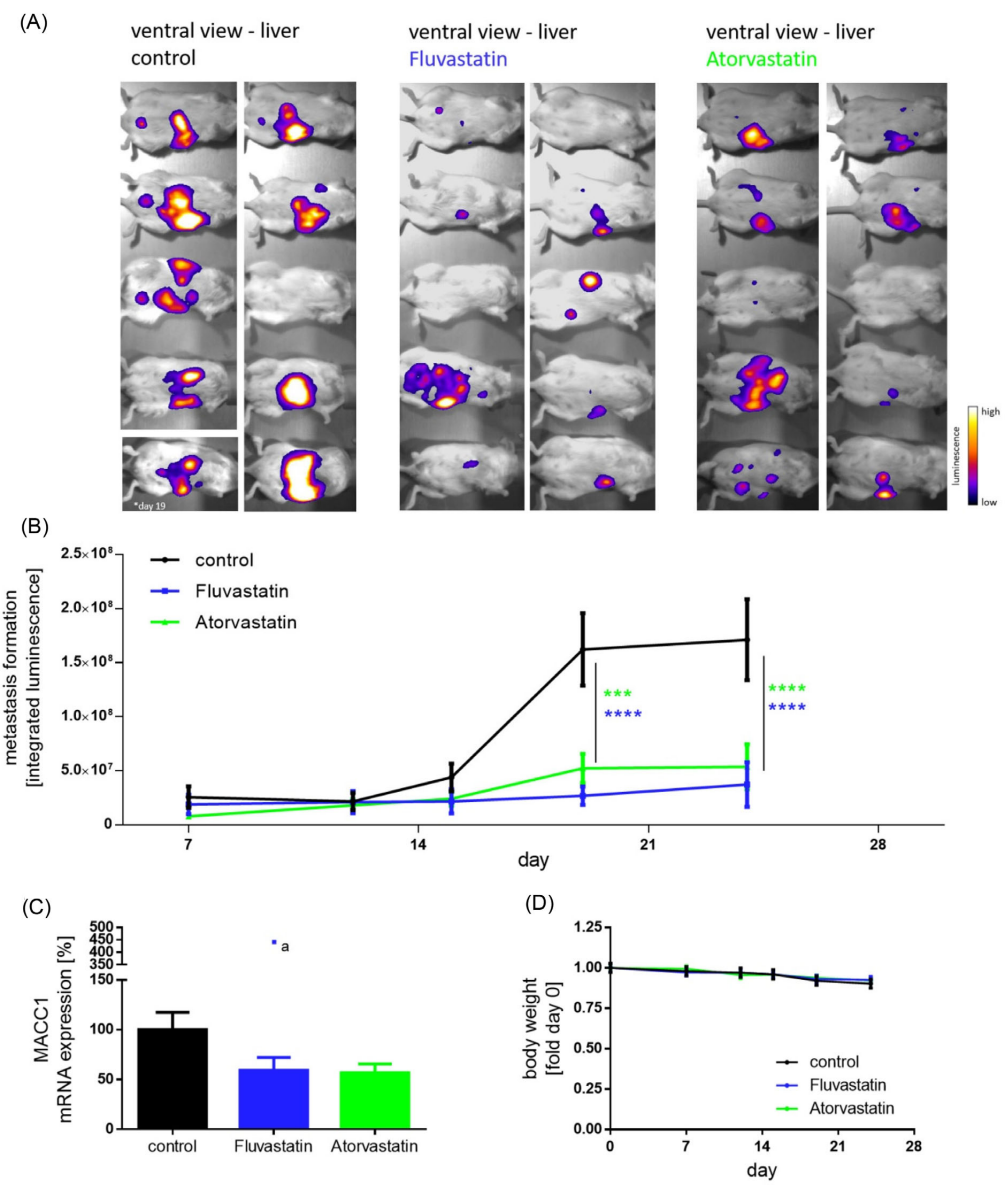
Statin treatment decreases tumour burden and metastasis formation in vivo. Intra‐splenically xenografted (HCT116/CMVp‐Luc cells) SCID‐beige mice were treated either with solvent or daily doses of 13 mg/kg body weight fluvastatin or atorvastatin. Bioluminescent imaging of animals from a ventral view (A), at day 24 of statin treatment, showed significantly weaker signals, indicating restricted metastasis formation in the liver. Bioluminescent signals from all ten animals over the time course of the experiment were quantified (B). Metastasis‐associated in colon cancer 1 (MACC1) transcripts in the liver were also found to be reduced in the fluvastatin and atorvastatin treated groups (C). The constant body weight of animals in each group indicates ethical conditions throughout the experiment (D). Results represent means ± SEM and significant results were determined by two‐way analysis of variance (ANOVA) and Dunnet's multiple comparison test with a 95% CI (* = *p* < 0.05, ** = *p* < 0.01, *** = *p* < 0.001, **** = *p* < 0.0001). One outlier, marked by an *a in the plot, was identified by the ROUT method (Q = 1%) and Grubbs test (α = 0.01); that date was not included in calculating the mean MACC1 mRNA expression levels in livers. Data were analyzed by ANOVA and Dunnett's multiple comparison post‐tests or two‐way ANOVA including Tukey's additivity test

Further, we used real‐world evidence (RWE) approach to examine associations between statin use and the prevalence of various cancers among patients with positive diagnoses. We conducted a retrospective, two‐centre (University Virginia, VA, USA and Charité Universitätsmedizin Berlin, Germany) observational, cohort‐based, nested case‐control study, and evaluated all statin‐taking patients relative to control groups.

We considered 308 990 patients enrolled between 2008 and 2018. After data pre‐processing (Figure 3, Figure S3, and Table S1), 277 980 patients were included. Simvastatin and atorvastatin (71% and 19% of the study populations), pravastatin (5%), fluvastatin (2%), rosuvastatin (2%) and lovastatin (1%) were considered.

**FIGURE 3:**
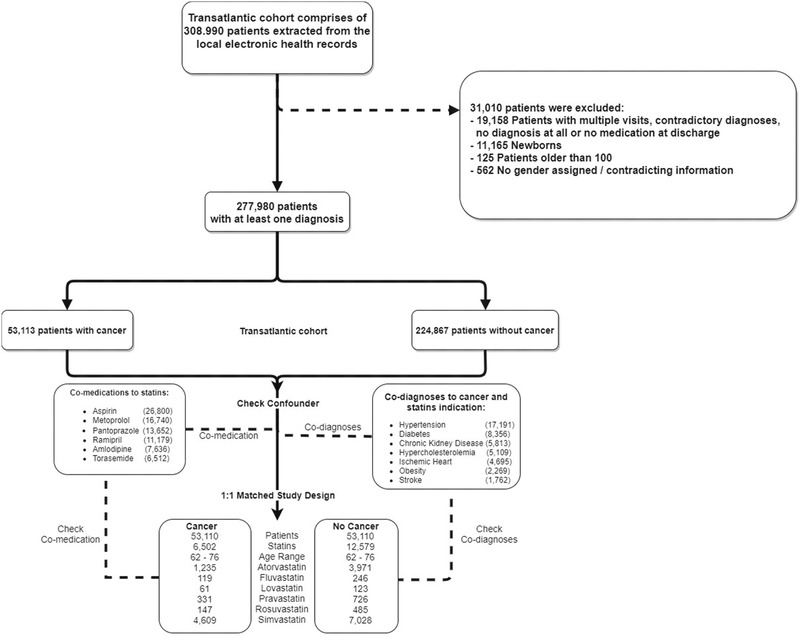
Data pre‐processing and cohort design. Patient records from databases at the Charité and UVA Health System were merged into a trans‐Atlantic cohort. From the resulting 432 333 records, 31 010 were excluded (upper‐right panel) due to incompleteness or contradictory information; this pre‐processing yielded a final trans‐Atlantic cohort of 277 980 patients. Of these, 53 113 were diagnosed with cancer, where ‘cancer’ is defined based on ICD10 codes in the range of ‘C00’ to ‘D48’ (excluding codes ‘D10’ to ‘D37’, which describe benign neoplasms). Co‐medications prescribed along with statins (lower‐left), as well as co‐diagnoses with cancer and statin indications (lower‐right), were included in the confounder analysis and calculated for both the full trans‐Atlantic and 1:1 matched cohorts

Notably, statin intake correlated with a significant reduction in cancer incidence (odds ratio [OR], .72; 95% confidence interval [CI], .70–.74). For statin‐taking patients, we calculated a higher cancer survival probability versus subjects not taking any statins (Cox proportional‐hazards model; hazards ratio [HR], .64; 95% CI, .48–.86). We found no difference for low‐dose (20 mg: HR, 1.1; 95% CI, 0.89–1.37) versus high‐dose (80 mg: HR, 1.11; 95% CI, .83–1.48) treatments; similarly, low‐dose (10–20 mg: HR, .80; 95% CI, .59–1.09) and high‐dose atorvastatin regimes (80 mg: HR, .72; 95% CI, .51–1.02) were comparable. When considering each statin separately, we found a strong cancer‐preventive effect for atorvastatin (OR, .41; 95% CI, .38–.43) and significant effects for fluvastatin (OR, .7; 95% CI, .57–.85), pravastatin (OR, .63; 95% CI, .56–.71) and rosuvastatin (OR, .43; 95% CI, .36–.51); simvastatin showed only a weak cancer‐preventive effect (OR, .9; 95% CI, .87–.94), and lovastatin effects were not readily assessed (relatively few patients; broad confidence intervals; Table S2).

We also considered the clinical data as a 1:1 matched‐study design, using propensity score‐matched sub‐cohorts to better control for confounding associations that might stem from different distributions of age and gender between the whole dataset and the subset of cancer patients. We discovered further evidence supporting the cancer‐preventive effect of statins (Figure 4 and Figure S4): (i) all statins considered together had an OR of .5 (95% CI, .48–.51), with (ii) atorvastatin .30 (95% CI, .28–.32), fluvastatin .65 (95% CI, .47–.88), lovastatin .51 (95% CI, .38–.7), pravastatin .47 (95% CI, .42–.54), rosuvastatin .32 (95% CI, .26–.38), and simvastatin .63 (95% CI, .61–.66).

**FIGURE 4:**
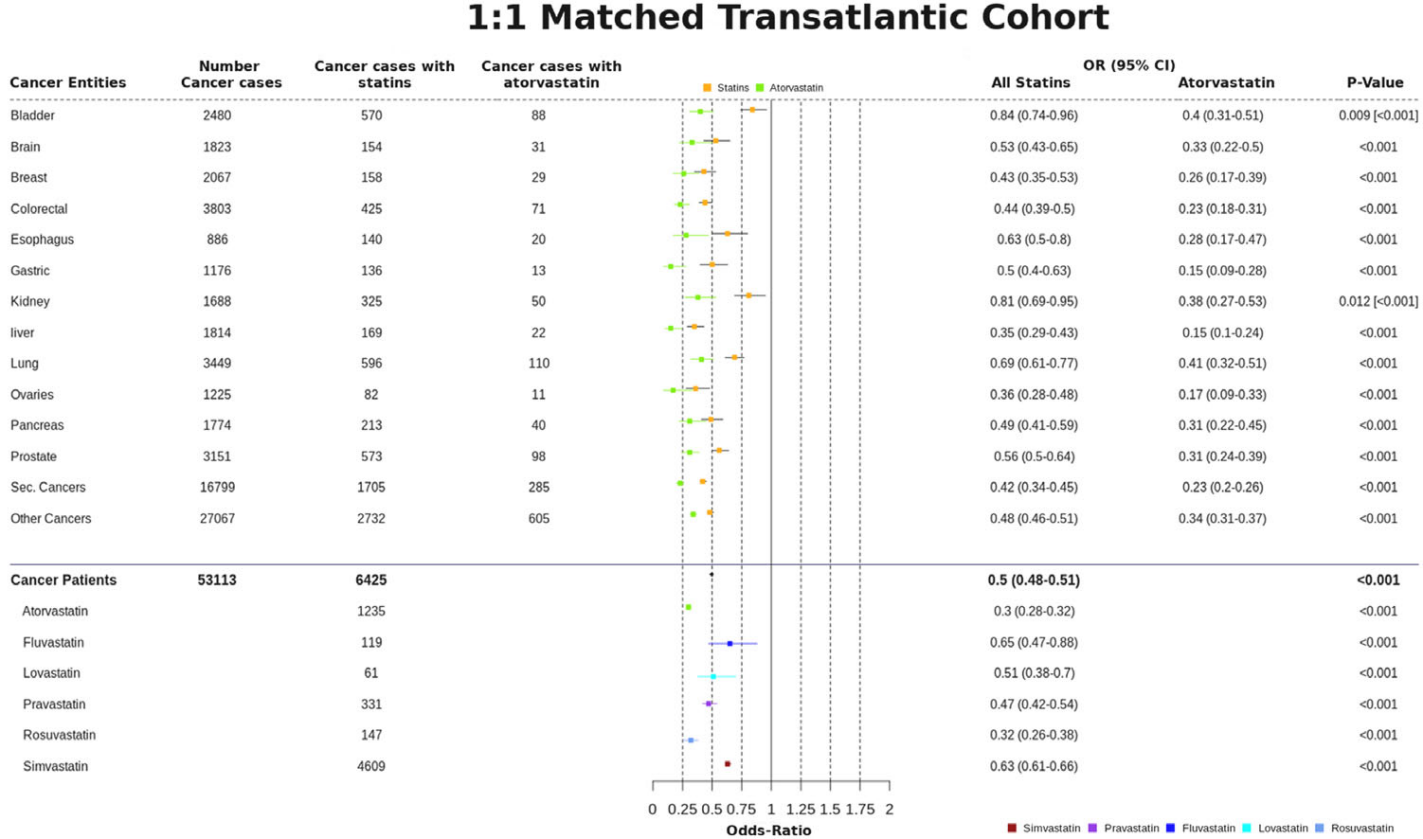
Cancer preventive effect of statins in the 1:1 matched trans‐Atlantic cohort for different cancer entities, and comparison with different statins separately. The cancer‐preventive effects of statins as a group, and for atorvastatin separately, were calculated for a number of different cancer types, as indicated in this summary overview of (i) cancer incidences, (ii) cancer diagnoses and (iii) prescribed statins. Cancer preventive effects are calculated as odds ratio (OR) for both statins and atorvastatin. The 95% CIs are supplied in addition to *p*‐values; *p*‐values are provided in square brackets for atorvastatin, for any values that differ from the full statin set. An overview of the different statins prescribed in the study population is provided at the bottom of the figure. Note the significant and systematic deviation—considered across different cancers—for atorvastatin in comparison to other statins

To account for confounders, we examined drugs prescribed alongside statins (Figure S5). Only aspirin and furosemide exhibit ORs < 1; all other co‐medications did not show any cancer‐preventive effect. After excluding aspirin and furosemide in our 1:1 matched study design, we still found a cancer‐preventive effect for all statins (OR of .756, 95% CI .72–.80) and atorvastatin alone (OR of .63; 95% CI, .57–.70) (Table S3). Statin‐specific co‐diagnoses with cancer were sorted in descending order of appearance/occurrence (Table S4). The overall outcome shows a slight OR increase, but the cancer‐preventive effect of statins remains unaffected. We conclude a real (significant) signal indicative of chemopreventive effect for statins, despite any influence of confounders.

RWE revealed a statistically significant link between statin usage and cancer development in patients. Statins exert cancer‐preventive effects (up to 50% risk reductions), relating different cancer types and different statins. Superior chemoprevention via statins exists for liver cancer (OR .35, 95% CI .29–.43; confirming the meta‐analysis^5^) and CRC (OR .44, 95% CI .39–.50^6^) or secondary neoplasms (OR .42, 95% CI .30–.45).

Finally, we extended the RWE results by utilizing a clinical research platform (TriNetX) to access a large, international cohort of anonymized electronic health record (EHR) data (aggregate statistics only). Our initial RWE was based on 53 000 cancer patients in our two independent trans‐Atlantic cohorts; the results from the TriNetX analysis, reflecting another 132 072 patients, are consistent with these single‐site findings of the cancer‐preventive effect of statins.

This study extends our previous work^2^ by corroborating, broadening (via EHR data) and deepening (via MACC1‐based experiments) prior knowledge about the cancer‐preventive effect of statins.^5^, ^6^, ^7^, ^8^, ^9^, ^10^ Many clinically employed statins diminish MACC1 expression in CRC, pancreatic and gastric cancer cells, confirming the cross‐entity–wide effects detected by our RWE study. The chemopreventive influence of simvastatin (OR .63, 95% CI .61–.66) and atorvastatin (OR .3, 95% CI .28–.32) correlates with their capacity to reduce MACC1 expression, with IC_50_ values of 3.1 and 1.6 μM, respectively (Figure 1H).

In conclusion, our study revealed strong evidence for cancer‐preventive effects of statins in a large trans‐Atlantic cohort, comprised of long‐term statin users. We link this beneficial effect to MACC1 transcriptional inhibition, yielding inhibited tumour growth and metastasis formation. These two lines of evidence suggest statin use in treating cancers, for which MACC1 serves as a predictive biomarker and interventional target necessitating prospective, randomized clinical trials.

## CONFLICT OF INTEREST

The authors declare that there is no conflict of interest.

## FUNDING INFORMATION

This work was supported by TRR295, KFO339 and DFG PR1562/1‐1 (RP). Portions of this work were also supported by the University of Virginia School of Data Science and by NSF CAREER award MCB‐1350957.

This work was also supported in part by the German Cancer Consortium (DKTK) and SPARK‐BIH Berlin (US).

## Supporting information

SUPPORTING INFORMATION
