# Supplementary material for: Real‐world evidence for preventive effects of statins on cancer incidence: A trans‐Atlantic analysis
Source: Clin Transl Med. 2022 Feb 20;12(2):e726. doi: 10.1002/ctm2.726 (PMC8858616; doi:10.1002/ctm2.726)
Supplement: Supplementary file 1 — SUPPORTING INFORMATION [file CTM2-12-e726-s001.pdf]

# A Trans-Atlantic Analysis of Real-world Evidences showing preventive Effects of Statins on Cancer Incidence

---

TABLE OF CONTENTS


| TABLE OF CONTENTS | Pages |
|---|---|
| Supplementary Methods | 2-9 |
| Supplementary Table 1 | 10-11 |
| Supplementary Table 2 | 12-13 |
| Supplementary Table 3 | 14-41 |
| Supplementary Table 4 | 42-44 |
| Supplementary Figure 1 | 45 |
| Supplementary Figure 2 | 46 |
| Supplementary Figure 3 | 47 |
| Supplementary Figure 4 | 48-49 |
| Supplementary Figure 5 | 50 |


---

## Supplementary Methods

### Study population

The total study population includes patients from the Charité and UVA hospitals. Therefore, in an initial data-processing stage, all diagnoses and discharge medications were extracted, merged, unified and transformed into a centralized database. The medications for the Charité datasets were available in the doctor letters at discharge. The medications were automatically extracted based on a complete list of German drug products, including an ingredient mapping. At the UVA hospital, an EHR was established in 2010, in which the discharge medication could be extracted. Cancer was defined by ICD10 diagnoses codes from 'C00' to 'D48' with an exclusion of ICD10 codes 'D10' to 'D37'. Newborns and patients older than 100 years were excluded from the study.

This process yielded in 277,980 distinct patients, of whom 53,113 patients were diagnosed with cancer (Age and gender distribution is visualized in Supplementary Figure 2).

All patient records were anonymized prior to study cohort selection in accordance with the Health Insurance Portability and Accountability Act (HIPAA) [1]. The Ethical Review Committee of the Charité – Universitätsmedizin Berlin granted formal approval for this study (EA1/171/15), as well as the Institutional Review Board (IRB) of the University of Virginia (UVA IRB-HSR Study Tracking # 22282). A Data Use Agreement (DUA) between UVA and Charite, as regards the Statins project, was finally executed.

### Trial design / Cohort design

The RWE for treatment of cancer with statins in a large scale, multicentric, randomized observational study was scrutinized. The Charité patient data, as well as the UVA patients, were separately analyzed, and also considered in a transatlantic cohort. A detailed overview of the dataset is shown in Supplementary Table 1. Patients with a cancer diagnosis were randomly matched 1:1 with non-cancer patients according to age and gender.

Given statistically significant large case counts, subtypes of cancer were analyzed. High frequentcy cancer entities, like breast, colorectal, liver, pancreas or prostate were separately considered. In this context (based on sufficient data), Atorvastatin, Fluvastatin, Lovastatin, Pravastatin, Rosuvastatin and Simvastatin were inspected individually.

The primary end-point of this study describes the status of patients at discharge considering the transatlantic cohort. Due to incomplete data, patient progression is not available for a significant number of cases. The second endpoint describes the 1:1 matched study including cancer subtypes by considering the different statins.

**Confounder / Co-medication**

Different confounding factors were considered during the planning phase. Because of the variably in prescription or diagnosis habits by doctors, the data could be biased. Here, the study design was set-up as a transatlantic cohort, including two completely independent datasets, to minimize such effects. In this regard, other confounding factors, such as family history, will be minimized.

Further, to mitigate the risk of describing false-positive cancer preventive effect of statins, co-medication for every patient was extensively studied. Therefore, the most commonly prescribed drugs along with statins were considered. Each drug was then evaluated independently of each other (Figure 3 - Co-Medication). Specifically, we also analyzed whether co-diagnoses (based on ICD10 codes) like hypercholesterolemia (E78 & Z83), ischemic heart (I20-I25), hypertension (I10), stroke (I63), diabetes (E10-E14), obesity (E65-E68) or chronical kidney diseases (N18) influence the conclusion reached in our study.

**Statistical analysis**

Our primary goal was to establish whether or not statins have a cancer preventive effect. RWE studies generally cannot prove a causal inference, given effect modifiers and confounding factors. A stratified analysis was performed in this study, considering an overall cancer preventive effect for the collection of all prescribed statins as well as analyzing different cancer subtypes and individual statins separately; moreover, it is also for this reason that the study was conducted across two distinct cohorts, one in Germany and the other in the U.S.

As common measures of the effect size in randomized controlled trials, Odds ratio (OR) and risk ratio (RR) were calculated for the respective case and control groups. To account for the study design, a 1:1 matched analysis was performed, taking into consideration the contingency-table for different statins and cancer subtypes. In order to overcome artifacts from the matching process, this procedure was performed 25 times and OR and RR were considered as mean and median values.

Both measurements are reported with the 95%-confidence intervals and p-values. All p-values are two-sided for which 0.025 were considered to indicate statistical significance. We statistically analyzed the mRNA expression data with a one-way analysis of variance (ANOVA) procedure, including Dunnett´s multiple comparison test and a confidence interval of 95%. The functional assays were analyzed using two-way ANOVA, including Turkey´s additivity test and a confidence interval of 95% (* = $p < 0.05$, ** = $p < 0.01$, *** = $p < 0.001$, **** = $p < 0.0001$).

**Cell lines and growth conditions**

Human cancer cell lines HCT116 (CRC), HT-29 (CRC), SW48 (CRC), BxPC3 (pancreatic cancer) were purchased from the American Type Culture Collection and MKN45 (gastric cancer) was kindly provided by Experimentelle Pharmakologie & Onkologie Berlin-Buch GmbH (EPO GmbH, Berlin, Germany). Cells were grown in DMEM or RPMI1640 medium (Thermo Fisher Scientific, Waltham, Massachusetts) supplemented with 10% fetal bovine serum (Bio & Sell, Feucht, Germany) in a humidified incubator at 37°C with 5% $CO_2$. Cell lines were regularly tested for mycoplasma using the MycoAlert Mycoplasma

detection kit (Lonza, Basel, Switzerland). We verified the authentication of all cell lines by short tandem repeat (STR) genotyping at the Leibniz-Institute DSMZ (Braunschweig, Germany): STR genotypes concurred with published genotypes.

Drug treatment was performed with statins purchased from Selleckchem (Houston, Texas) (atorvastatin) or Thermo Fisher Scientific (Waltham, Massachusetts) (fluvastatin, simvastatin, lovastatin). The 20 mM stock solutions were prepared freshly for every application in dimethylsulfoxide (DMSO). Control cells were treated with equal amounts of solvent to rule out adverse effects caused by DMSO.

**Transfection of human CRC cells**

Cell line generation for HTS was described previously [2]. For constitutive or inducible MACC1 overexpression, cells were transduced with lentiviruses produced in HEK293T cells using the plasmids RC224774L2 (MACC1-GFP, Origene, Rockville, Maryland) or pCW-GW-rtTA/MACC1-P2A-nLuc (kindly provided by Dr. Nikolas Gunkel, DKFZ, Heidelberg) and pMD2.G, psPAX2 for packaging following standard protocols. pMD2.G and psPAX2 were kind gifts from Didier Trono (Addgene plasmid # 12259, # 12260). Cells were selected either by FACS or blasticidin (Invivogen, San Diego, California) treatment. Knock-out of MACC1 in HCT116 cells (HCT116/MACC1-/-) was performed according to the protocol by Ran et al. with the vector pSpCas9(BB)-2A-GFP (Addgene, Watertown, Massachusetts) and the single guide RNAs: sgMACC1 fwd – GTT TGA AGA GTA CCC GGG TTT GG and sgMACC1 rev – ACA TGC CTT GCT CCG TAT GCA GG (Biotez, Berlin, Germany) [3].

**High throughput drug screening (HTS)**

We seeded HCT116-MACC1p-Luc CRC cells stably expressing the MACC1-promoter driven luciferase gene in 384-well plates (Perkin Elmer, Waltham, Massachusetts) at 4,000 cells/well. The plates already contained the 4241 compounds in two different concentrations: 5 µg/ml and 0.5 µg/ml of Prestwick library compounds, and 1 µM and 0.1 µM of NIH and Microsource library compounds, respectively.

Cells were incubated with compounds for 24 h at 37°C in a humidified incubator with 5% $CO_2$. To assess the luminescent signal, plates were treated with 25 µl BriteLite plus luminescent reagent for 5 min (Perkin Elmer, Waltham, Massachusetts) and measured in an Envision Reader with ultrasensitive luminescence detector (Perkin Elmer, Waltham, Massachusetts).

**In vivo validation of statins**

Animal experiments were conducted according to the United Kingdom Coordinating Committee of Cancer Research (UKCCCR) guidelines and granted by the State Office of Health and Social Affairs (Landesamt für Gesundheit und Soziales, LaGeSo, Berlin, Germany).

We transplanted $3x10^5$ of HCT116/CMVp-Luc cells into the spleens of 6-week-old female SCID-beige mice (Charles River, Wilmington, Massachusetts). Mice were randomly assigned to 3 groups of 10 animals for oral application with daily doses of either solvent (10% Kolliphor in 0.9% NaCl) or a human equivalent dose of approximately 1 mg/kg body weight (13 mg/kg body weight) of fluvastatin or atorvastatin (in 0.9% NaCl). This dose is widely used in blood lipid reduction therapy [4,5]. We continuously monitored tumor growth and metastasis formation via intraperitoneal application of 150 mg/kg D-luciferin (Biosynth, Staad, Switzerland) and the bioluminescence imaging system NightOWL LB 981 (Berthold Technologies, Bad Wildbad, Germany). For imaging and quantification, WinLight (Berthold Technologies) and ImageJ (version 1.51j8, National Institutes of Health, USA) were used. The experiment was terminated due to ethical reasons. Afterwards, spleens and livers were removed and shock frozen in liquid nitrogen for preparation of cryosections and further analysis. Extraction of RNA and genomic DNA with subsequent qRT-PCR and qPCR analysis was performed as described previously.

For immunohistochemistry (IHC) cryosections were fixed with 4% para-formaldehyde (PFA, Carl Roth GmbH & Co. KG, Karlsruhe, Germany) and quenched with 0.1 M glycine (Carl Roth GmbH & Co. KG, Karlsruhe, Germany). Blocking of endogenous peroxidases with 3% $H_2O_2$ (Carl Roth GmbH & Co. KG, Karlsruhe, Germany) was followed by permeabilization with 0.2% TritonX100 and blocking with 5%

bovine serum albumin (BSA). Primary antibody (1:200 in 2.5% BSA, anti-CK19; OriGene Technologies Inc., Rockville, Maryland) incubation was carried out for 2 h followed by 5 washing steps and 1 h secondary antibody incubation (1:500 in 2.5% BSA, anti-rabbit-HRP; Promega, Madison, Wisconsin) at room temperature. Final staining with DAB solution (DAKO DAB Liquid + chromogen substrate; Agilent Technologies, Santa Clara, California) and nuclear staining with hemalum (Carl Roth GmbH & Co. KG, Karlsruhe, Germany) was followed by washing under running tab water. Slices were mounted on microscope slides with Glycergel (Agilent Technologies, Santa Clara, California) and imaged with a Keyence BZ-X800 microscope (Keyence, Osaka, Japan).

**RNA extraction and qRT-PCR**

Drug treatment was performed in 12-well dishes with $1.25x10^5$ cells/well. To isolate total RNA, we used the Universal RNA Purification Kit (Roboklon, Berlin, Germany) according to manufacturer´s instructions. RNA was quantified (Nanodrop, Peqlab, Erlangen, Germany) and 50 ng RNA were applied to reverse transcription with random hexamers in a reaction mix (5 mM MgCl2, 1x RT-buffer, 4 mM pooled dNTPs, 1 U/µl RNAse inhibitor and 2.5 U/µL Moloney Murine Leukemia Virus reverse transcriptase; Thermo Fisher Scientific) at 23°C for 15 min, 42°C for 45 min, 99°C for 5 min with subsequent cooling at 4°C for 5 min. The LightCycler 480 (Roche Diagnostics, Mannheim, Germany) with GoTaq dye (Promega, Fitchburg, Wisconsin) chemistry was employed for cDNA amplification in quantitative polymerase chain reaction (qPCR) under following conditions: 95°C for 2 min followed by 45 cycles of 95°C for 7 s, 60°C for 10 s and 72°C for 5 s. Primers for MACC1 and G6PD were described previously [20]. Data was analyzed with the LightCycler 480 Software release 1.5.0SP3 (RocheDiagnostics). Duplicate qRT-PCR reaction values were averaged and each mean value of the expressed gene was normalized to the respective mean amount of the G6PD cDNA.

**Protein extraction and Western blotting**

Similar to RNA extraction, 1.25x$10^5$ cells were seeded in 12-well plates. After drug treatment, cells were scraped off in ice cold RIPA buffer (50 mM Tris, 150 mM NaCl and 1% Nonidet P-40; pH 7.5 supplemented with complete protease inhibitor tablets; Roche Diagnostics) and lysed for 30 min on ice. We used the Bicinchoninic Acid Protein Assays Reagent (Thermo Fisher Scientific) to determine protein concentration according to manufacturer´s instructions. Equal amounts of protein were separated by SDS-PAGE and transferred to PVDF membranes (Bio-Rad Laboratories Inc., Hercules, California). Membranes were blocked for 1 h at room temperature with 5% BSA in TBST buffer (10mM Tris-HCl, 0.1% Tween20 and 150 mM NaCl; pH 7.5). Incubation of membranes with rabbit anti-MACC1 antibody (Sigma-Aldrich, dilution 1:10,000) or mouse anti-β-actin (Sigma-Aldrich, dilution 1:25,000) and vinculin (Sigma-Aldrich, 1:2,000) at 4°C overnight was followed by incubation with HRP-conjugated anti-rabbit IgG (Promega, dilution 1:20,000) or anti-mouse IgG (Thermo Fisher Scientific, dilution 1:20,000) for 1 h at room temperature. Antibody-protein complexes were visualized with WesternBright ECL HRP substrate (Advansta, Menlo Park, California) and subsequent exposure to CL-Xposure Films (Thermo Fisher Scientific). Immunoblotting for β-actin and vinculin served as protein loading control.

**Proliferation assay**

Cell viability and proliferation was assessed using the IncuCyte® ZOOM System (Essen BioScience, Ann Arbor, Michigan). 5x$10^3$ cells were seeded in 96-well plates and allowed to accommodate for 24 h before drug treatment. After drug treatment, plates were placed into the IncuCyte for 72 h at 37°C in a humidified incubator with 5% $CO_2$. Four pictures of each well were taken every 2 h which allowed a detailed analysis of the area covered by proliferating cells over time. For data analysis and export the IncuCyte ZOOM software 2016B was used. Each cell proliferation experiment was performed in triplicate. Results are expressed as confluence percent area under the curve of cell confluence compared normalized to solvent-treated controls.

**Clonogenic assay**

We used the clonogenic assay to assess the reproductive viability and ability to form colonies. 400 cells/well of HCT116/GFP and HCT116/MACC1-GFP cells were seeded in 6-well dishes and allowed to attach for 24 h before drug treatment. After drug treatment, plates were placed into a humidified incubator at 37°C with 5% $CO_2$ for 7 days. Medium was removed and the colonies were fixed and stained with a solution of PBS containing 1% formaldehyde and 0.1% crystal violet. The colony covered area was determined with the colony area plug in of ImageJ (version 1.51j8, National Institutes of Health, USA). Results are expressed as area percent compared to solvent-treated.

References for Supplementary Methods


1. Caplan RM. HIPAA. Health Insurance Portability and Accountability Act of 1996. Dent Assist. 2003; 72: 6-8.
2. Juneja M, Kobelt D, Walther W, Voss C, Smith J, Specker E, et al. Statin and rottlerin small-molecule inhibitors restrict colon cancer progression and metastasis via MACC1. PLoS Biol. 2017; 15: e2000784.
3. Ran FA, Hsu PD, Wright J, Agarwala V, Scott DA, Zhang F. Genome engineering using the CRISPR-Cas9 system. Nat Protoc. 2013; 8: 2281-308.
4. Nair AB, Jacob S. A simple practice guide for dose conversion between animals and human. J Basic Clin Pharm. 2016; 7: 27-31.
5. Stone NJ, Robinson JG, Lichtenstein AH, Bairey Merz CN, Blum CB, Eckel RH, et al. 2013 ACC/AHA guideline on the treatment of blood cholesterol to reduce atherosclerotic cardiovascular risk in adults: a report of the American College of Cardiology/American Heart Association Task Force on Practice Guidelines. J Am Coll Cardiol. 2014; 63: 2889-934.

**Supplementary Table 1: Statistical characteristics of the sampled population and study design. [caption on next page]**

| Characteristics | Full Trans-Atlantic Cohort | | | 1:1 Matched Cohort | | |
|---|---|---|---|---|---|---|
| | Statin use? | | | Statin use? | | |
| | ✓ | ✗ | *p*-value | ✓ | ✗ | *p*-value |
| All Subjects (#) | 42,682 | 235,298 | | 17,941 | 88,279 | |
| Age (yrs) | | | | | | |
| Median | 69 | 54 | | 68 | 59 | |
| Interquartile range | 60–76 | 34–69 | | 62–76 | 51–72 | |
| Gender (# [%]) | | | < 0.001 | | | < 0.001 |
| ♀ | 16,903 [40] | 114,640 [49] | | 11,718 [65] | 46,669 [52] | |
| ♂ | 25,779 [60] | 120,658 [51] | | 6,223 [34] | 41,610 [47] | |
| Cancer patients by Age (median yrs) | | | | | | |
| ♀ | 70 | 59 | | 70 | 59 | |
| ♂ | 70 | 61 | | 70 | 60 | |
| Cancer patients by Gender (# [%]) | | | < 0.001 | | | < 0.001 |
| ♀ | 2,154 [33] | 21,791* [46] | | 2,154 [33] | 21,790 [46] | |
| ♂ | 4,349* [67] | 24,896 [54] | | 4,348 [67] | 24,896 [54] | |
| Censored Confounders (# subjects) | | | | | | |
| Hypercholesterolemia | | | < 0.001 | | | |
| ♀ | 987 | 13,366 | | | | |
| ♂ | 2,098 | 16,744 | | | | |
| Hypertension | | | < 0.001 | | | |
| ♀ | 623 | 9,240 | | | | |
| ♂ | 1,391 | 11,370 | | | | |
| Ischemic heart | | | < 0.001 | | | |
| ♀ | 1,369 | 13,269 | | | | |
| ♂ | 2,151 | 15,974 | | | | |
| Stroke | | | < 0.001 | | | |
| ♀ | 1,630 | 13,669 | | | | |
| ♂ | 3,514 | 17,390 | | | | |
| Obesity | | | < 0.001 | | | |
| ♀ | 1,589 | 13,231 | | | | |
| ♂ | 3,429 | 16,918 | | | | |
| Diabetes | | | < 0.001 | | | |
| ♀ | 1,178 | 11,914 | | | | |
| ♂ | 2,398 | 14,342 | | | | |
| Chronic kidney disease | | | < 0.001 | | | |
| ♀ | 1,451 | 12,758 | | | | |
| ♂ | 2,989 | 15,782 | | | | |

This Table summarizes characteristic features and properties of the population sampled in this work, for both of the trans-Atlantic cohorts (Charite, UVA) as well as the 1:1 matched cohort. Both cohorts are split into statin and non-statin patients. If determined, a p-value for the underlying condition is provided. The censored confounders are included as well as interquartile ranges for the age distribution for statin and non-statin users. Note that the columns in the bottom half of the table (i.e., below the Censored Confounders section) contain values that apply to both cohorts, "full trans-Atlantic" and "1:1 matched": this is intrinsic to the layout of the table (the "Statin use?" query applies in both cohorts), and thus the data are not replicated in the bottom-half columns. A similar pattern occurs in parts of the top-half of the table too, though the data there are explicitly provided because the pairs of numerical values are not perfectly identical (e.g., the "Cancer patients by Gender" row has a discrepancy of two patients between the two cohorts, for the parameters marked by '*' in the first pair of columns).

**Supplementary Table 2**

In this table, the co-diagnoses of cancer were analyzed, taking into consideration statin intake as well. In tab 1 “Counts”, co-diagnoses are calculated. The resulting diagnoses are presented via a short description as well as the ICD10 code. “Counts” consists of the number of patients, also diagnosed together with cancer in descending order. The second tab “Odds-Ratios”, shows the ORs. The first results row displays the OR for cancer given (statins, atorvastatin, simvastatin) without exclusion of any co-diagnoses. Results are displayed for the transatlantic as well as 1:1 matched cohort.

**Tab 1:**

| Co-diagnoses | | |
|---|---|---|
| ICD10-Code | Counts | Description |
| Z11 | 35.397 | Encounter for screening for infectious and parasitic diseases |
| I10 | 33.633 | Essential primary hypertension |
| E87 | 16.140 | Other disorders of fluid electrolyte and acid-base balance |
| Z45 | 15.226 | Encounter for adjustment and management of implanted device |
| Z92 | 13.022 | Personal history of medical treatment |
| E11 | 12.614 | Type 2 diabetes mellitus |
| N18 | 10.697 | Chronic kidney disease CKD |
| Z29 | 9.638 | Encounter for other prophylactic measures |
| Z95 | 9.472 | Presence of cardiac and vascular implants and grafts |
| I25 | 8.454 | Chronic ischemic heart disease |
| E78 | 8.361 | Disorders of lipoprotein metabolism and other lipidemias |
| E03 | 7.921 | Other hypothyroidism |
| Z90 | 7.354 | Acquired absence of organs not elsewhere classified |
| Z43 | 7.193 | Encounter for attention to artificial openings |

**Tab 2**

# Odds-Ratios

TRANSATLANTIC COHORT

| | Exclusion | Drug | #Drug / Diagnosis | OR | Conf-low | Conf-up | P-value |
|---|---|---|---|---|---|---|---|
| cancer | | atorvastatin | 1235 | 0,408 | 0,380 | 0,430 | 1,35005195E-155 |
| cancer | Diabetes | atorvastatin | 517 | 0,490 | 0,450 | 0,540 | 5,31000000E-48 |
| cancer | Hypercholesterolemia | atorvastatin | 407 | 0,699 | 0,630 | 0,780 | 1,41000000E-10 |
| cancer | Ischemic Heart | atorvastatin | 452 | 0,490 | 0,440 | 0,540 | 1,16000000E-38 |
| cancer | Stroke | atorvastatin | 642 | 0,583 | 0,540 | 0,630 | 5,18000000E-39 |
| cancer | Hypertension | atorvastatin | 268 | 0,615 | 0,540 | 0,700 | 9,36000000E-13 |
| cancer | CKD | atorvastatin | 601 | 0,467 | 0,430 | 0,510 | 1,85000000E-61 |
| cancer | Obesity | atorvastatin | 664 | 0,469 | 0,430 | 0,510 | 8,24000000E-61 |

1:1 MATCHED COHORT

| | | Drug | #Drug / Diagnosis | OR | Conf-low | Conf-up | P-value |
|---|---|---|---|---|---|---|---|
| cancer | | atorvastatin | 1235 | 0,299 | 0,280 | 0,32 | 5,347E-250 |
| cancer | Diabetes | atorvastatin | 517 | 0,335 | 0,302 | 0,37 | 8,180E-88 |
| cancer | Hypercholesterolemia | atorvastatin | 407 | 0,465 | 0,413 | 0,52 | 2,230E-33 |
| cancer | Ischemic Heart | atorvastatin | 452 | 0,340 | 0,305 | 0,38 | 2,910E-75 |
| cancer | Stroke | atorvastatin | 642 | 0,406 | 0,370 | 0,45 | 3,000E-71 |
| cancer | Hypertension | atorvastatin | 268 | 0,387 | 0,335 | 0,45 | 2,530E-35 |
| cancer | CKD | atorvastatin | 601 | 0,328 | 0,299 | 0,36 | 4,180E-105 |
| cancer | Obesity | atorvastatin | 664 | 0,335 | 0,306 | 0,37 | 1,480E-108 |

ALL STATINS

TRANSATLANTIC COHORT

| Exclusion | Drug | #Drug / Diagnosis | #Drug / Diagnosis | OR | Conf-low | Conf-up | P-value |
|---|---|---|---|---|---|---|---|
| | statins | 53113 | 6426 | 0,72 | 0,70 | 0,74 | 2,1139E-108 |
| Diabetes | statins | 29834 | 3576 | 0,81 | 0,77 | 0,84 | 5,0600E-21 |
| Hypercholesterolemia | statins | 33198 | 3085 | 1,01 | 0,96 | 1,05 | 8,3842E-01 |
| Ischemic Heart | statins | 32766 | 3520 | 0,88 | 0,85 | 0,92 | 2,2900E-09 |
| Stroke | statins | 36205 | 5144 | 0,88 | 0,85 | 0,91 | 5,8400E-13 |
| Hypertension | statins | 22627 | 2014 | 0,94 | 0,89 | 0,99 | 2,2559E-02 |
| CKD | statins | 32982 | 4440 | 0,81 | 0,78 | 0,84 | 1,6800E-27 |
| Obesity | statins | 35169 | 5018 | 0,80 | 0,77 | 0,83 | 6,5700E-29 |

1:1 MATCHED COHORT

| | Drug | #Diagnoses | #Drug / Diagnosis | OR | Conf-low | Conf-up | P-value |
|---|---|---|---|---|---|---|---|
| | statins | 53113 | 6425 | 0,496 | 0,480 | 0,51 | 0,000E+00 |
| Diabetes | statins | 29834 | 3576 | 0,521 | 0,498 | 0,55 | 1,180E-154 |
| Hypercholesterolemia | statins | 33198 | 3085 | 0,643 | 0,613 | 0,68 | 1,210E-64 |
| Ischemic Heart | statins | 32766 | 3520 | 0,580 | 0,555 | 0,61 | 7,500E-111 |
| Stroke | statins | 36205 | 5144 | 0,584 | 0,562 | 0,61 | 1,300E-144 |
| Hypertension | statins | 22627 | 2014 | 0,546 | 0,515 | 0,58 | 3,990E-81 |
| CKD | statins | 32982 | 4440 | 0,530 | 0,508 | 0,55 | 3,150E-172 |
| Obesity | statins | 35169 | 5018 | 0,536 | 0,516 | 0,56 | 2,270E-195 |

SIMVASTATIN

TRANSATLANTIC COHORT

| Exclusion | Drug | #Drug / Diagnosis | OR | Conf-low | Conf-up | P-value |
|---|---|---|---|---|---|---|
| | simvastatin | 4609 | 0,904 | 0,870 | 0,940 | 4,85394737E-07 |
| Diabetes | simvastatin | 2814 | 0,897 | 0,860 | 0,940 | 2,30000000E-06 |
| Hypercholesterolemia | simvastatin | 2452 | 1,081 | 1,030 | 1,130 | 1,02896600E-03 |
| Ischemic Heart | simvastatin | 2845 | 1,007 | 0,960 | 1,050 | 7,72965426E-01 |
| Stroke | simvastatin | 4179 | 0,946 | 0,910 | 0,980 | 3,37182700E-03 |
| Hypertension | simvastatin | 1600 | 1,031 | 0,970 | 1,090 | 3,09244571E-01 |
| CKD | simvastatin | 3575 | 0,915 | 0,880 | 0,950 | 6,98000000E-06 |
| Obesity | simvastatin | 4038 | 0,898 | 0,870 | 0,930 | 6,40000000E-10 |

1:1 MATCHED COHORT

| | Drug | #Drug / Diagnosis | OR | Conf-low | Conf-up | P-value |
|---|---|---|---|---|---|---|
| | simvastatin | 4608 | 0,634 | 0,610 | 0,66 | 1,115E-100 |
| Diabetes | simvastatin | 2814 | 0,590 | 0,561 | 0,62 | 6,360E-82 |
| Hypercholesterolemia | simvastatin | 2452 | 0,699 | 0,662 | 0,74 | 6,840E-35 |
| Ischemic Heart | simvastatin | 2845 | 0,666 | 0,633 | 0,70 | 5,710E-51 |
| Stroke | simvastatin | 4179 | 0,640 | 0,613 | 0,67 | 9,330E-85 |
| Hypertension | simvastatin | 1600 | 0,601 | 0,563 | 0,64 | 3,350E-47 |
| CKD | simvastatin | 3575 | 0,615 | 0,588 | 0,64 | 1,590E-86 |
| Obesity | simvastatin | 4038 | 0,615 | 0,589 | 0,64 | 5,480E-96 |

Supplementary Table 2
Odds-Ratios

**Supplementary Table 3**

This table consists of the raw-results from the RWE study. Odds-Ratios and Relative-Risks are calculated for different statins and different cancer entities. Also both cohorts Charité and UVA were considered together in the transatlantic cohort as well as separately.

| | | | | | | | | |
|---|---|---|---|---|---|---|---|---|
| | | | | Odds-Ratio Transatlatic-Cohort<br>No Exclusion | | | | |
| Diagnose | Drug | NbDrug | NbDiag | NbDiag/Diag | Odds Ratio | Conf-Low | Conf-Up | P-Value |
| bladder | atorvastatin_charite_Cohort | 6420 | 2100 | 75 | 0,936 | 0,740 | 1,180 | 0,59048164 |
| brain | atorvastatin_charite_Cohort | 6439 | 1296 | 20 | 0,393 | 0,250 | 0,610 | 0,00004770 |
| breast | atorvastatin_charite_Cohort | 6436 | 1373 | 19 | 0,351 | 0,220 | 0,550 | 0,00000957 |
| cancer | atorvastatin_charite_Cohort | 6302 | 36203 | 711 | 0,470 | 0,430 | 0,510 | 0,00000000 |
| colorectal | atorvastatin_charite_Cohort | 6424 | 3151 | 50 | 0,404 | 0,300 | 0,530 | 0,00000000 |
| esophagus | atorvastatin_charite_Cohort | 6431 | 678 | 7 | 0,262 | 0,120 | 0,550 | 0,00059876 |
| kidney | atorvastatin_charite_Cohort | 6431 | 1530 | 45 | 0,763 | 0,570 | 1,030 | 0,07273126 |
| liver | atorvastatin_charite_Cohort | 6432 | 1651 | 17 | 0,261 | 0,160 | 0,420 | 0,00000008 |
| lung | atorvastatin_charite_Cohort | 6421 | 2630 | 69 | 0,678 | 0,530 | 0,860 | 0,00170116 |
| ovaries | atorvastatin_charite_Cohort | 6438 | 828 | 6 | 0,183 | 0,080 | 0,410 | 0,00005408 |
| pancreas | atorvastatin_charite_Cohort | 6435 | 1464 | 28 | 0,489 | 0,340 | 0,710 | 0,00015610 |
| prostate | atorvastatin_charite_Cohort | 6428 | 2409 | 59 | 0,631 | 0,490 | 0,820 | 0,00048792 |
| seq77 | atorvastatin_charite_Cohort | 6417 | 5507 | 73 | 0,336 | 0,270 | 0,420 | 0,00000000 |
| seq78 | atorvastatin_charite_Cohort | 6411 | 7200 | 73 | 0,254 | 0,200 | 0,320 | 0,00000000 |
| seq79 | atorvastatin_charite_Cohort | 6426 | 6024 | 81 | 0,340 | 0,270 | 0,420 | 0,00000000 |
| stomach | atorvastatin_charite_Cohort | 6438 | 1057 | 12 | 0,288 | 0,160 | 0,510 | 0,00003077 |
| bladder | atorvastatin_transatlantic_Cohort | 13837 | 2489 | 88 | 0,668 | 0,540 | 0,830 | 0,00025616 |
| brain | atorvastatin_transatlantic_Cohort | 13861 | 1815 | 31 | 0,315 | 0,220 | 0,450 | 0,00000000 |
| breast | atorvastatin_transatlantic_Cohort | 13854 | 2081 | 29 | 0,256 | 0,180 | 0,370 | 0,00000000 |
| cancer | atorvastatin_transatlantic_Cohort | 13460 | 52882 | 1235 | 0,408 | 0,380 | 0,430 | 0,00000000 |
| colorectal | atorvastatin_transatlantic_Cohort | 13841 | 3827 | 71 | 0,343 | 0,270 | 0,430 | 0,00000000 |
| esophagus | atorvastatin_transatlantic_Cohort | 13855 | 870 | 20 | 0,428 | 0,270 | 0,670 | 0,00027528 |
| kidney | atorvastatin_transatlantic_Cohort | 13853 | 1708 | 50 | 0,548 | 0,410 | 0,730 | 0,00005121 |
| liver | atorvastatin_transatlantic_Cohort | 13860 | 1822 | 22 | 0,222 | 0,150 | 0,340 | 0,00000000 |
| lung | atorvastatin_transatlantic_Cohort | 13822 | 3443 | 110 | 0,601 | 0,500 | 0,730 | 0,00000021 |
| ovaries | atorvastatin_transatlantic_Cohort | 13861 | 1219 | 11 | 0,165 | 0,090 | 0,300 | 0,00000001 |
| pancreas | atorvastatin_transatlantic_Cohort | 13855 | 1766 | 40 | 0,421 | 0,310 | 0,580 | 0,00000010 |
| prostate | atorvastatin_transatlantic_Cohort | 13837 | 3178 | 98 | 0,579 | 0,470 | 0,710 | 0,00000032 |
| seq77 | atorvastatin_transatlantic_Cohort | 13815 | 6925 | 106 | 0,282 | 0,230 | 0,340 | 0,00000000 |
| seq78 | atorvastatin_transatlantic_Cohort | 13795 | 9688 | 130 | 0,245 | 0,210 | 0,290 | 0,00000000 |
| seq79 | atorvastatin_transatlantic_Cohort | 13816 | 8363 | 148 | 0,326 | 0,280 | 0,380 | 0,00000000 |
| stomach | atorvastatin_transatlantic_Cohort | 13863 | 1176 | 13 | 0,203 | 0,120 | 0,350 | 0,00000001 |
| bladder | atorvastatin_UVA_Cohort | 7417 | 389 | 13 | 0,421 | 0,240 | 0,730 | 0,00235283 |
| brain | atorvastatin_UVA_Cohort | 7422 | 519 | 11 | 0,263 | 0,140 | 0,480 | 0,00002599 |
| breast | atorvastatin_UVA_Cohort | 7418 | 708 | 10 | 0,174 | 0,090 | 0,320 | 0,00000011 |
| cancer | atorvastatin_UVA_Cohort | 7158 | 16679 | 524 | 0,379 | 0,350 | 0,420 | 0,00000000 |
| colorectal | atorvastatin_UVA_Cohort | 7417 | 676 | 21 | 0,390 | 0,250 | 0,600 | 0,00002990 |
| esophagus | atorvastatin_UVA_Cohort | 7424 | 192 | 13 | 0,885 | 0,500 | 1,550 | 0,68521530 |
| kidney | atorvastatin_UVA_Cohort | 7422 | 178 | 5 | 0,352 | 0,140 | 0,860 | 0,02395751 |
| liver | atorvastatin_UVA_Cohort | 7428 | 171 | 5 | 0,367 | 0,150 | 0,890 | 0,02710168 |
| lung | atorvastatin_UVA_Cohort | 7401 | 813 | 41 | 0,649 | 0,470 | 0,890 | 0,00795611 |
| ovaries | atorvastatin_UVA_Cohort | 7423 | 391 | 5 | 0,157 | 0,070 | 0,380 | 0,00002180 |
| pancreas | atorvastatin_UVA_Cohort | 7420 | 302 | 12 | 0,504 | 0,280 | 0,900 | 0,02126562 |
| prostate | atorvastatin_UVA_Cohort | 7409 | 769 | 39 | 0,651 | 0,470 | 0,900 | 0,00958381 |
| seq77 | atorvastatin_UVA_Cohort | 7398 | 1418 | 33 | 0,290 | 0,210 | 0,410 | 0,00000000 |
| seq78 | atorvastatin_UVA_Cohort | 7384 | 2488 | 57 | 0,285 | 0,220 | 0,370 | 0,00000000 |
| seq79 | atorvastatin_UVA_Cohort | 7390 | 2339 | 67 | 0,359 | 0,280 | 0,460 | 0,00000000 |
| stomach | atorvastatin_UVA_Cohort | 7425 | 119 | 1 | 0,103 | 0,010 | 0,740 | 0,03811341 |
| bladder | fluvastatin_charite_Cohort | 860 | 2119 | 12 | 1,105 | 0,620 | 1,960 | 0,74682672 |
| brain | fluvastatin_charite_Cohort | 864 | 1295 | 0 | 0,000 | 0,000 | nan | nan |
| breast | fluvastatin_charite_Cohort | 861 | 1370 | 2 | 0,281 | 0,070 | 1,130 | 0,07323226 |
| cancer | fluvastatin_charite_Cohort | 810 | 36199 | 118 | 0,639 | 0,530 | 0,780 | 0,00000718 |
| colorectal | fluvastatin_charite_Cohort | 863 | 3162 | 8 | 0,486 | 0,240 | 0,980 | 0,04403310 |
| esophagus | fluvastatin_charite_Cohort | 862 | 683 | 5 | 1,425 | 0,590 | 3,440 | 0,43930927 |
| kidney | fluvastatin_charite_Cohort | 857 | 1535 | 14 | 1,800 | 1,060 | 3,060 | 0,02949958 |
| liver | fluvastatin_charite_Cohort | 863 | 1656 | 4 | 0,465 | 0,170 | 1,240 | 0,13103053 |
| lung | fluvastatin_charite_Cohort | 861 | 2633 | 8 | 0,586 | 0,290 | 1,180 | 0,13568962 |
| ovaries | fluvastatin_charite_Cohort | 864 | 825 | 1 | 0,233 | 0,030 | 1,650 | 0,15467825 |
| pancreas | fluvastatin_charite_Cohort | 864 | 1462 | 2 | 0,262 | 0,070 | 1,050 | 0,05213356 |
| prostate | fluvastatin_charite_Cohort | 860 | 2420 | 12 | 0,965 | 0,550 | 1,710 | 0,90975237 |
| seq77 | fluvastatin_charite_Cohort | 854 | 5530 | 10 | 0,350 | 0,190 | 0,650 | 0,00086204 |
| seq78 | fluvastatin_charite_Cohort | 856 | 7220 | 18 | 0,480 | 0,300 | 0,770 | 0,00232437 |
| seq79 | fluvastatin_charite_Cohort | 855 | 6028 | 18 | 0,581 | 0,360 | 0,930 | 0,02470929 |
| stomach | fluvastatin_charite_Cohort | 862 | 1050 | 1 | 0,183 | 0,030 | 1,300 | 0,07696706 |
| bladder | fluvastatin_transatlantic_Cohort | 870 | 2517 | 12 | 1,446 | 0,820 | 2,560 | 0,20570011 |
| brain | fluvastatin_transatlantic_Cohort | 874 | 1813 | 0 | 0,000 | 0,000 | nan | nan |
| breast | fluvastatin_transatlantic_Cohort | 871 | 2087 | 2 | 0,286 | 0,070 | 1,150 | 0,07922611 |
| cancer | fluvastatin_transatlantic_Cohort | 820 | 53012 | 119 | 0,697 | 0,570 | 0,850 | 0,00042853 |
| colorectal | fluvastatin_transatlantic_Cohort | 873 | 3844 | 9 | 0,701 | 0,360 | 1,350 | 0,29606307 |
| esophagus | fluvastatin_transatlantic_Cohort | 872 | 878 | 5 | 1,719 | 0,710 | 4,150 | 0,23125908 |
| kidney | fluvastatin_transatlantic_Cohort | 867 | 1719 | 14 | 2,498 | 1,470 | 4,250 | 0,00076389 |
| liver | fluvastatin_transatlantic_Cohort | 873 | 1825 | 4 | 0,656 | 0,250 | 1,750 | 0,40292487 |

Supplementary Table 3
Odds-Ratio Transatlantic Cohort

| | | | | | | | | |
|---|---|---|---|---|---|---|---|---|
| lung | fluvastatin_transatlantic_Cohort | 871 | 3469 | 8 | 0,692 | 0,340 | 1,390 | 0,30978342 |
| ovaries | fluvastatin_transatlantic_Cohort | 874 | 1219 | 1 | 0,244 | 0,030 | 1,740 | 0,17414313 |
| pancreas | fluvastatin_transatlantic_Cohort | 874 | 1769 | 2 | 0,337 | 0,080 | 1,350 | 0,13148467 |
| prostate | fluvastatin_transatlantic_Cohort | 870 | 3208 | 12 | 1,131 | 0,640 | 2,000 | 0,68504579 |
| seq77 | fluvastatin_transatlantic_Cohort | 864 | 6974 | 10 | 0,432 | 0,230 | 0,810 | 0,00895920 |
| seq78 | fluvastatin_transatlantic_Cohort | 866 | 9744 | 18 | 0,555 | 0,350 | 0,890 | 0,01333511 |
| seq79 | fluvastatin_transatlantic_Cohort | 865 | 8395 | 18 | 0,649 | 0,410 | 1,030 | 0,06540593 |
| stomach | fluvastatin_transatlantic_Cohort | 872 | 1173 | 1 | 0,255 | 0,040 | 1,810 | 0,16060165 |
| bladder | fluvastatin_UVA_Cohort | 10 | 398 | 0 | 0,000 | 0,000 | nan | nan |
| brain | fluvastatin_UVA_Cohort | 10 | 518 | 0 | 0,000 | 0,000 | nan | nan |
| breast | fluvastatin_UVA_Cohort | 10 | 717 | 0 | 0,000 | 0,000 | nan | nan |
| cancer | fluvastatin_UVA_Cohort | 10 | 16813 | 1 | 0,542 | 0,070 | 4,280 | 0,57105569 |
| colorectal | fluvastatin_UVA_Cohort | 10 | 682 | 1 | 15,394 | 1,950 | 121,670 | 0,00950846 |
| esophagus | fluvastatin_UVA_Cohort | 10 | 195 | 0 | 0,000 | 0,000 | nan | nan |
| kidney | fluvastatin_UVA_Cohort | 10 | 184 | 0 | 0,000 | 0,000 | nan | nan |
| liver | fluvastatin_UVA_Cohort | 10 | 169 | 0 | 0,000 | 0,000 | nan | nan |
| lung | fluvastatin_UVA_Cohort | 10 | 836 | 0 | 0,000 | 0,000 | nan | nan |
| ovaries | fluvastatin_UVA_Cohort | 10 | 394 | 0 | 0,000 | 0,000 | nan | nan |
| pancreas | fluvastatin_UVA_Cohort | 10 | 307 | 0 | 0,000 | 0,000 | nan | nan |
| prostate | fluvastatin_UVA_Cohort | 10 | 788 | 0 | 0,000 | 0,000 | nan | nan |
| seq77 | fluvastatin_UVA_Cohort | 10 | 1444 | 0 | 0,000 | 0,000 | nan | nan |
| seq78 | fluvastatin_UVA_Cohort | 10 | 2524 | 0 | 0,000 | 0,000 | nan | nan |
| seq79 | fluvastatin_UVA_Cohort | 10 | 2367 | 0 | 0,000 | 0,000 | nan | nan |
| stomach | fluvastatin_UVA_Cohort | 10 | 123 | 0 | 0,000 | 0,000 | nan | nan |
| bladder | pravastatin_charite_Cohort | 947 | 2121 | 18 | 1,514 | 0,950 | 2,420 | 0,08172758 |
| brain | pravastatin_charite_Cohort | 950 | 1294 | 2 | 0,269 | 0,070 | 1,080 | 0,05955814 |
| breast | pravastatin_charite_Cohort | 951 | 1373 | 5 | 0,636 | 0,260 | 1,540 | 0,32342309 |
| cancer | pravastatin_charite_Cohort | 914 | 36213 | 178 | 0,907 | 0,770 | 1,070 | 0,24746687 |
| colorectal | pravastatin_charite_Cohort | 948 | 3157 | 6 | 0,331 | 0,150 | 0,740 | 0,00663901 |
| esophagus | pravastatin_charite_Cohort | 950 | 684 | 3 | 0,769 | 0,250 | 2,400 | 0,66191383 |
| kidney | pravastatin_charite_Cohort | 946 | 1535 | 11 | 1,271 | 0,700 | 2,310 | 0,43937018 |
| liver | pravastatin_charite_Cohort | 949 | 1652 | 1 | 0,105 | 0,010 | 0,750 | 0,04039165 |
| lung | pravastatin_charite_Cohort | 948 | 2631 | 12 | 0,802 | 0,450 | 1,420 | 0,46055382 |
| ovaries | pravastatin_charite_Cohort | 950 | 826 | 6 | 1,280 | 0,570 | 2,870 | 0,56083404 |
| pancreas | pravastatin_charite_Cohort | 949 | 1462 | 5 | 0,599 | 0,250 | 1,440 | 0,25402749 |
| prostate | pravastatin_charite_Cohort | 949 | 2423 | 23 | 1,697 | 1,120 | 2,570 | 0,01250808 |
| seq77 | pravastatin_charite_Cohort | 938 | 5526 | 24 | 0,777 | 0,520 | 1,170 | 0,22461704 |
| seq78 | pravastatin_charite_Cohort | 943 | 7215 | 22 | 0,534 | 0,350 | 0,820 | 0,00391808 |
| seq79 | pravastatin_charite_Cohort | 938 | 6023 | 18 | 0,528 | 0,330 | 0,840 | 0,00738607 |
| stomach | pravastatin_charite_Cohort | 952 | 1053 | 4 | 0,664 | 0,250 | 1,780 | 0,42126692 |
| bladder | pravastatin_transatlantic_Cohort | 2590 | 2516 | 18 | 0,723 | 0,450 | 1,150 | 0,17631538 |
| brain | pravastatin_transatlantic_Cohort | 2596 | 1812 | 6 | 0,331 | 0,150 | 0,740 | 0,00663901 |
| breast | pravastatin_transatlantic_Cohort | 2597 | 2092 | 9 | 0,431 | 0,220 | 0,830 | 0,01290727 |
| cancer | pravastatin_transatlantic_Cohort | 2485 | 52982 | 331 | 0,631 | 0,560 | 0,710 | 0,00000000 |
| colorectal | pravastatin_transatlantic_Cohort | 2591 | 3836 | 12 | 0,313 | 0,180 | 0,550 | 0,00005350 |
| esophagus | pravastatin_transatlantic_Cohort | 2596 | 879 | 4 | 0,457 | 0,170 | 1,220 | 0,11931669 |
| kidney | pravastatin_transatlantic_Cohort | 2590 | 1717 | 13 | 0,765 | 0,440 | 1,320 | 0,34459225 |
| liver | pravastatin_transatlantic_Cohort | 2595 | 1821 | 2 | 0,110 | 0,030 | 0,440 | 0,00132245 |
| lung | pravastatin_transatlantic_Cohort | 2584 | 3459 | 25 | 0,732 | 0,490 | 1,090 | 0,12618731 |
| ovaries | pravastatin_transatlantic_Cohort | 2595 | 1219 | 9 | 0,744 | 0,390 | 1,440 | 0,38140528 |
| pancreas | pravastatin_transatlantic_Cohort | 2593 | 1768 | 8 | 0,454 | 0,230 | 0,910 | 0,02421165 |
| prostate | pravastatin_transatlantic_Cohort | 2590 | 3206 | 41 | 1,308 | 0,960 | 1,780 | 0,08794177 |
| seq77 | pravastatin_transatlantic_Cohort | 2579 | 6965 | 27 | 0,390 | 0,270 | 0,570 | 0,00000113 |
| seq78 | pravastatin_transatlantic_Cohort | 2570 | 9722 | 40 | 0,414 | 0,300 | 0,570 | 0,00000012 |
| seq79 | pravastatin_transatlantic_Cohort | 2572 | 8379 | 35 | 0,421 | 0,300 | 0,590 | 0,00000079 |
| stomach | pravastatin_transatlantic_Cohort | 2597 | 1175 | 4 | 0,341 | 0,130 | 0,910 | 0,02995527 |
| bladder | pravastatin_UVA_Cohort | 1643 | 395 | 0 | 0,000 | 0,000 | nan | nan |
| brain | pravastatin_UVA_Cohort | 1646 | 518 | 4 | 0,443 | 0,170 | 1,190 | 0,10075232 |
| breast | pravastatin_UVA_Cohort | 1646 | 719 | 4 | 0,318 | 0,120 | 0,850 | 0,02162087 |
| cancer | pravastatin_UVA_Cohort | 1571 | 16769 | 153 | 0,527 | 0,450 | 0,620 | 0,00000000 |
| colorectal | pravastatin_UVA_Cohort | 1643 | 679 | 6 | 0,509 | 0,230 | 1,140 | 0,09792977 |
| esophagus | pravastatin_UVA_Cohort | 1646 | 195 | 1 | 0,294 | 0,040 | 2,100 | 0,22779074 |
| kidney | pravastatin_UVA_Cohort | 1644 | 182 | 2 | 0,634 | 0,160 | 2,560 | 0,53012127 |
| liver | pravastatin_UVA_Cohort | 1646 | 169 | 1 | 0,339 | 0,050 | 2,420 | 0,27783347 |
| lung | pravastatin_UVA_Cohort | 1636 | 828 | 13 | 0,918 | 0,530 | 1,590 | 0,77285988 |
| ovaries | pravastatin_UVA_Cohort | 1645 | 393 | 3 | 0,438 | 0,140 | 1,370 | 0,15651161 |
| pancreas | pravastatin_UVA_Cohort | 1644 | 306 | 3 | 0,565 | 0,180 | 1,760 | 0,33135588 |
| prostate | pravastatin_UVA_Cohort | 1641 | 783 | 18 | 1,352 | 0,850 | 2,160 | 0,20652570 |
| seq77 | pravastatin_UVA_Cohort | 1641 | 1439 | 3 | 0,119 | 0,040 | 0,370 | 0,00019509 |
| seq78 | pravastatin_UVA_Cohort | 1627 | 2507 | 18 | 0,415 | 0,260 | 0,660 | 0,00023613 |
| seq79 | pravastatin_UVA_Cohort | 1634 | 2356 | 17 | 0,416 | 0,260 | 0,670 | 0,00030595 |
| stomach | pravastatin_UVA_Cohort | 1645 | 122 | 0 | 0,000 | 0,000 | nan | nan |
| bladder | simvastatin_charite_Cohort | 22402 | 2105 | 431 | 1,733 | 1,560 | 1,930 | 0,00000000 |
| brain | simvastatin_charite_Cohort | 22371 | 1300 | 104 | 0,579 | 0,470 | 0,710 | 0,00000032 |
| breast | simvastatin_charite_Cohort | 22372 | 1366 | 102 | 0,537 | 0,440 | 0,660 | 0,00000000 |

| | | | | | | | | |
|---|---|---|---|---|---|---|---|---|
| cancer | simvastatin_charite_Cohort | 22271 | 36506 | 4257 | 0,883 | 0,850 | 0,920 | 0,00000000 |
| colorectal | simvastatin_charite_Cohort | 22364 | 3139 | 319 | 0,755 | 0,670 | 0,850 | 0,00000485 |
| esophagus | simvastatin_charite_Cohort | 22370 | 704 | 98 | 1,080 | 0,870 | 1,340 | 0,49473117 |
| kidney | simvastatin_charite_Cohort | 22368 | 1523 | 243 | 1,272 | 1,110 | 1,460 | 0,00061550 |
| liver | simvastatin_charite_Cohort | 22358 | 1646 | 140 | 0,620 | 0,520 | 0,740 | 0,00000018 |
| lung | simvastatin_charite_Cohort | 22366 | 2646 | 415 | 1,249 | 1,120 | 1,390 | 0,00006325 |
| ovaries | simvastatin_charite_Cohort | 22375 | 833 | 53 | 0,452 | 0,340 | 0,600 | 0,00000007 |
| pancreas | simvastatin_charite_Cohort | 22372 | 1473 | 155 | 0,784 | 0,660 | 0,930 | 0,00544550 |
| prostate | simvastatin_charite_Cohort | 22393 | 2401 | 389 | 1,297 | 1,160 | 1,450 | 0,00000641 |
| seq77 | simvastatin_charite_Cohort | 22319 | 5374 | 510 | 0,703 | 0,640 | 0,770 | 0,00000000 |
| seq78 | simvastatin_charite_Cohort | 22300 | 7141 | 576 | 0,583 | 0,530 | 0,640 | 0,00000000 |
| seq79 | simvastatin_charite_Cohort | 22348 | 5961 | 540 | 0,665 | 0,610 | 0,730 | 0,00000000 |
| stomach | simvastatin_charite_Cohort | 22363 | 1059 | 116 | 0,821 | 0,680 | 1,000 | 0,04463320 |
| bladder | simvastatin_transatlantic_Cohort | 25863 | 2502 | 437 | 1,999 | 1,800 | 2,220 | 0,00000000 |
| brain | simvastatin_transatlantic_Cohort | 25830 | 1818 | 113 | 0,620 | 0,510 | 0,750 | 0,00000167 |
| breast | simvastatin_transatlantic_Cohort | 25827 | 2077 | 116 | 0,553 | 0,460 | 0,670 | 0,00000000 |
| cancer | simvastatin_transatlantic_Cohort | 25608 | 53267 | 4609 | 0,904 | 0,870 | 0,940 | 0,00000049 |
| colorectal | simvastatin_transatlantic_Cohort | 25818 | 3816 | 329 | 0,886 | 0,790 | 0,990 | 0,03521205 |
| esophagus | simvastatin_transatlantic_Cohort | 25830 | 897 | 104 | 1,230 | 1,000 | 1,510 | 0,04856346 |
| kidney | simvastatin_transatlantic_Cohort | 25828 | 1705 | 243 | 1,564 | 1,360 | 1,790 | 0,00000000 |
| liver | simvastatin_transatlantic_Cohort | 25819 | 1814 | 141 | 0,790 | 0,660 | 0,940 | 0,00897216 |
| lung | simvastatin_transatlantic_Cohort | 25816 | 3472 | 442 | 1,377 | 1,250 | 1,520 | 0,00000000 |
| ovaries | simvastatin_transatlantic_Cohort | 25834 | 1224 | 59 | 0,473 | 0,360 | 0,620 | 0,00000011 |
| pancreas | simvastatin_transatlantic_Cohort | 25831 | 1778 | 161 | 0,933 | 0,790 | 1,100 | 0,41921404 |
| prostate | simvastatin_transatlantic_Cohort | 25848 | 3183 | 408 | 1,385 | 1,250 | 1,540 | 0,00000000 |
| seq77 | simvastatin_transatlantic_Cohort | 25766 | 6807 | 534 | 0,803 | 0,730 | 0,880 | 0,00000550 |
| seq78 | simvastatin_transatlantic_Cohort | 25735 | 9644 | 616 | 0,640 | 0,590 | 0,690 | 0,00000000 |
| seq79 | simvastatin_transatlantic_Cohort | 25791 | 8314 | 579 | 0,703 | 0,650 | 0,770 | 0,00000000 |
| stomach | simvastatin_transatlantic_Cohort | 25824 | 1182 | 119 | 1,050 | 0,870 | 1,270 | 0,62571728 |
| bladder | simvastatin_UVA_Cohort | 3461 | 397 | 6 | 0,410 | 0,180 | 0,920 | 0,03189318 |
| brain | simvastatin_UVA_Cohort | 3459 | 518 | 9 | 0,473 | 0,240 | 0,910 | 0,02743977 |
| breast | simvastatin_UVA_Cohort | 3455 | 711 | 14 | 0,538 | 0,320 | 0,910 | 0,01992112 |
| cancer | simvastatin_UVA_Cohort | 3337 | 16761 | 352 | 0,575 | 0,510 | 0,640 | 0,00000000 |
| colorectal | simvastatin_UVA_Cohort | 3454 | 677 | 10 | 0,401 | 0,210 | 0,750 | 0,00493380 |
| esophagus | simvastatin_UVA_Cohort | 3460 | 193 | 6 | 0,859 | 0,380 | 1,940 | 0,72790244 |
| kidney | simvastatin_UVA_Cohort | 3460 | 182 | 0 | 0,000 | 0,000 | nan | nan |
| liver | simvastatin_UVA_Cohort | 3461 | 168 | 1 | 0,160 | 0,020 | 1,140 | 0,07522293 |
| lung | simvastatin_UVA_Cohort | 3450 | 826 | 27 | 0,910 | 0,620 | 1,340 | 0,64425689 |
| ovaries | simvastatin_UVA_Cohort | 3459 | 391 | 6 | 0,417 | 0,190 | 0,930 | 0,03059616 |
| pancreas | simvastatin_UVA_Cohort | 3459 | 305 | 6 | 0,537 | 0,240 | 1,210 | 0,13206059 |
| prostate | simvastatin_UVA_Cohort | 3455 | 782 | 19 | 0,667 | 0,420 | 1,050 | 0,08284148 |
| seq77 | simvastatin_UVA_Cohort | 3447 | 1433 | 24 | 0,457 | 0,310 | 0,690 | 0,00014003 |
| seq78 | simvastatin_UVA_Cohort | 3435 | 2503 | 40 | 0,436 | 0,320 | 0,600 | 0,00000035 |
| seq79 | simvastatin_UVA_Cohort | 3443 | 2353 | 39 | 0,452 | 0,330 | 0,620 | 0,00000115 |
| stomach | simvastatin_UVA_Cohort | 3461 | 123 | 3 | 0,669 | 0,210 | 2,100 | 0,50384829 |
| bladder | statins_charite_Cohort | 30087 | 2094 | 548 | 1,699 | 1,540 | 1,870 | 0,00000000 |
| brain | statins_charite_Cohort | 30054 | 1302 | 127 | 0,512 | 0,430 | 0,620 | 0,00000000 |
| breast | statins_charite_Cohort | 30048 | 1363 | 128 | 0,491 | 0,410 | 0,590 | 0,00000000 |
| cancer | statins_charite_Cohort | 29926 | 36497 | 5295 | 0,790 | 0,760 | 0,820 | 0,00000000 |
| cancersubentities | statins_charite_Cohort | 30019 | 21890 | 3344 | 0,858 | 0,830 | 0,890 | 0,00000000 |
| colorectal | statins_charite_Cohort | 30046 | 3133 | 386 | 0,667 | 0,600 | 0,740 | 0,00000000 |
| esophagus | statins_charite_Cohort | 30045 | 699 | 115 | 0,937 | 0,770 | 1,140 | 0,52629374 |
| kidney | statins_charite_Cohort | 30047 | 1513 | 318 | 1,270 | 1,120 | 1,440 | 0,00021278 |
| liver | statins_charite_Cohort | 30037 | 1645 | 162 | 0,518 | 0,440 | 0,610 | 0,00000000 |
| lung | statins_charite_Cohort | 30038 | 2651 | 507 | 1,129 | 1,020 | 1,250 | 0,01919563 |
| ovaries | statins_charite_Cohort | 30054 | 837 | 66 | 0,406 | 0,320 | 0,520 | 0,00000000 |
| pancreas | statins_charite_Cohort | 30053 | 1476 | 191 | 0,706 | 0,610 | 0,820 | 0,00000524 |
| prostate | statins_charite_Cohort | 30077 | 2395 | 492 | 1,235 | 1,120 | 1,360 | 0,00002466 |
| seq77 | statins_charite_Cohort | 29994 | 5354 | 628 | 0,633 | 0,580 | 0,690 | 0,00000000 |
| seq78 | statins_charite_Cohort | 29966 | 7125 | 699 | 0,513 | 0,470 | 0,560 | 0,00000000 |
| seq79 | statins_charite_Cohort | 30040 | 5955 | 671 | 0,602 | 0,550 | 0,650 | 0,00000000 |
| stomach | statins_charite_Cohort | 30041 | 1058 | 132 | 0,677 | 0,560 | 0,810 | 0,00004065 |
| bladder | statins_transatlantic_Cohort | 43286 | 2480 | 570 | 1,593 | 1,450 | 1,750 | 0,00000000 |
| brain | statins_transatlantic_Cohort | 43261 | 1823 | 154 | 0,488 | 0,410 | 0,580 | 0,00000000 |
| breast | statins_transatlantic_Cohort | 43242 | 2067 | 158 | 0,438 | 0,370 | 0,520 | 0,00000000 |
| cancer | statins_transatlantic_Cohort | 42682 | 53113 | 6426 | 0,716 | 0,700 | 0,740 | 0,00000000 |
| colorectal | statins_transatlantic_Cohort | 43238 | 3803 | 425 | 0,667 | 0,600 | 0,740 | 0,00000000 |
| esophagus | statins_transatlantic_Cohort | 43249 | 886 | 140 | 0,997 | 0,830 | 1,190 | 0,97639961 |
| kidney | statins_transatlantic_Cohort | 43251 | 1688 | 325 | 1,269 | 1,120 | 1,430 | 0,00014824 |
| liver | statins_transatlantic_Cohort | 43248 | 1814 | 169 | 0,544 | 0,460 | 0,640 | 0,00000000 |
| lung | statins_transatlantic_Cohort | 43207 | 3449 | 596 | 1,114 | 1,020 | 1,220 | 0,01797753 |
| ovaries | statins_transatlantic_Cohort | 43257 | 1225 | 82 | 0,380 | 0,300 | 0,480 | 0,00000000 |
| pancreas | statins_transatlantic_Cohort | 43252 | 1774 | 213 | 0,724 | 0,630 | 0,840 | 0,00001351 |
| prostate | statins_transatlantic_Cohort | 43258 | 3151 | 573 | 1,184 | 1,080 | 1,300 | 0,00038379 |
| seq77 | statins_transatlantic_Cohort | 43154 | 6753 | 693 | 0,608 | 0,560 | 0,660 | 0,00000000 |

Supplementary Table 3
Odds-Ratio Transatlantic Cohort

| | | | | | | | | |
|---|---|---|---|---|---|---|---|---|
| seq78 | statins_transatlantic_Cohort | 43085 | 9574 | 827 | 0,499 | 0,460 | 0,540 | 0,00000000 |
| seq79 | statins_transatlantic_Cohort | 43178 | 8269 | 808 | 0,574 | 0,530 | 0,620 | 0,00000000 |
| stomach | statins_transatlantic_Cohort | 43249 | 1176 | 136 | 0,694 | 0,580 | 0,830 | 0,00007447 |
| bladder | statins_UVA_Cohort | 13199 | 386 | 22 | 0,396 | 0,260 | 0,610 | 0,00002502 |
| brain | statins_UVA_Cohort | 13207 | 521 | 27 | 0,358 | 0,240 | 0,530 | 0,00000056 |
| breast | statins_UVA_Cohort | 13194 | 704 | 30 | 0,291 | 0,200 | 0,420 | 0,00000000 |
| cancer | statins_UVA_Cohort | 12756 | 16616 | 1131 | 0,465 | 0,440 | 0,500 | 0,00000000 |
| cancersubentities | statins_UVA_Cohort | 12987 | 7514 | 501 | 0,465 | 0,420 | 0,510 | 0,00000000 |
| colorectal | statins_UVA_Cohort | 13192 | 670 | 39 | 0,405 | 0,290 | 0,560 | 0,00000012 |
| esophagus | statins_UVA_Cohort | 13204 | 187 | 25 | 1,013 | 0,660 | 1,540 | 0,95663733 |
| kidney | statins_UVA_Cohort | 13204 | 175 | 7 | 0,273 | 0,130 | 0,580 | 0,00070476 |
| liver | statins_UVA_Cohort | 13211 | 169 | 7 | 0,283 | 0,130 | 0,600 | 0,00126272 |
| lung | statins_UVA_Cohort | 13169 | 798 | 89 | 0,827 | 0,660 | 1,030 | 0,09405443 |
| ovaries | statins_UVA_Cohort | 13203 | 388 | 16 | 0,282 | 0,170 | 0,460 | 0,00000091 |
| pancreas | statins_UVA_Cohort | 13199 | 298 | 22 | 0,523 | 0,340 | 0,810 | 0,00347392 |
| prostate | statins_UVA_Cohort | 13181 | 756 | 81 | 0,789 | 0,630 | 0,990 | 0,03951355 |
| seq77 | statins_UVA_Cohort | 13160 | 1399 | 65 | 0,320 | 0,250 | 0,410 | 0,00000000 |
| seq78 | statins_UVA_Cohort | 13119 | 2449 | 128 | 0,361 | 0,300 | 0,430 | 0,00000000 |
| seq79 | statins_UVA_Cohort | 13138 | 2314 | 137 | 0,413 | 0,350 | 0,490 | 0,00000000 |
| stomach | statins_UVA_Cohort | 13208 | 118 | 4 | 0,230 | 0,080 | 0,620 | 0,00494112 |

| | | Relative Risk Transatlatic-Cohort No Exclusion | | | | |
|---|---|---|---|---|---|---|
| Diagnose | Drug | RelativRisk | Conf-Upper | Conf-Lower | P-Value | NNT-ALL |
| bladder | atorvastatin_charite_Cohort | 1,214 | 1,483 | 0,994 | 0,05703431 | -370,59 |
| brain | atorvastatin_charite_Cohort | 0,413 | 0,636 | 0,269 | 0,00006509 | 216,13 |
| breast | atorvastatin_charite_Cohort | 0,371 | 0,576 | 0,239 | 0,00001249 | 189,85 |
| cancer | atorvastatin_charite_Cohort | 0,584 | 0,623 | 0,548 | 0,00000000 | 11,27 |
| colorectal | atorvastatin_charite_Cohort | 0,510 | 0,654 | 0,398 | 0,00000017 | 106,70 |
| esophagus | atorvastatin_charite_Cohort | 0,373 | 0,695 | 0,200 | 0,00196494 | 382,57 |
| kidney | atorvastatin_charite_Cohort | 0,858 | 1,134 | 0,650 | 0,28435302 | 766,64 |
| liver | atorvastatin_charite_Cohort | 0,353 | 0,533 | 0,234 | 0,00000103 | 153,13 |
| lung | atorvastatin_charite_Cohort | 0,791 | 0,986 | 0,635 | 0,03643041 | 301,53 |
| ovaries | atorvastatin_charite_Cohort | 0,184 | 0,410 | 0,082 | 0,00004414 | 241,38 |
| pancreas | atorvastatin_charite_Cohort | 0,559 | 0,792 | 0,394 | 0,00113989 | 254,64 |
| prostate | atorvastatin_charite_Cohort | 0,765 | 0,966 | 0,606 | 0,02412469 | 291,94 |
| seq77 | atorvastatin_charite_Cohort | 0,469 | 0,570 | 0,386 | 0,00000000 | 56,66 |
| seq78 | atorvastatin_charite_Cohort | 0,348 | 0,424 | 0,285 | 0,00000000 | 35,15 |
| seq79 | atorvastatin_charite_Cohort | 0,417 | 0,508 | 0,342 | 0,00000000 | 47,15 |
| stomach | atorvastatin_charite_Cohort | 0,336 | 0,570 | 0,199 | 0,00005661 | 233,27 |
| bladder | atorvastatin_transatlantic_Cohort | 0,860 | 1,036 | 0,714 | 0,11211379 | 740,91 |
| brain | atorvastatin_transatlantic_Cohort | 0,356 | 0,497 | 0,255 | 0,00000000 | 218,77 |
| breast | atorvastatin_transatlantic_Cohort | 0,291 | 0,411 | 0,207 | 0,00000000 | 172,77 |
| cancer | atorvastatin_transatlantic_Cohort | 0,512 | 0,539 | 0,488 | 0,00000000 | 10,30 |
| colorectal | atorvastatin_transatlantic_Cohort | 0,411 | 0,509 | 0,331 | 0,00000000 | 113,77 |
| esophagus | atorvastatin_transatlantic_Cohort | 0,552 | 0,815 | 0,374 | 0,00283963 | 656,42 |
| kidney | atorvastatin_transatlantic_Cohort | 0,620 | 0,807 | 0,477 | 0,00039409 | 397,41 |
| liver | atorvastatin_transatlantic_Cohort | 0,283 | 0,410 | 0,195 | 0,00000000 | 195,24 |
| lung | atorvastatin_transatlantic_Cohort | 0,702 | 0,835 | 0,589 | 0,00008113 | 251,18 |
| ovaries | atorvastatin_transatlantic_Cohort | 0,210 | 0,356 | 0,124 | 0,00000001 | 263,58 |
| pancreas | atorvastatin_transatlantic_Cohort | 0,471 | 0,633 | 0,350 | 0,00000093 | 274,26 |
| prostate | atorvastatin_transatlantic_Cohort | 0,666 | 0,802 | 0,553 | 0,00002221 | 243,18 |
| seq77 | atorvastatin_transatlantic_Cohort | 0,374 | 0,442 | 0,317 | 0,00000000 | 59,35 |
| seq78 | atorvastatin_transatlantic_Cohort | 0,315 | 0,367 | 0,270 | 0,00000000 | 38,74 |
| seq79 | atorvastatin_transatlantic_Cohort | 0,395 | 0,458 | 0,341 | 0,00000000 | 51,07 |
| stomach | atorvastatin_transatlantic_Cohort | 0,234 | 0,389 | 0,140 | 0,00000005 | 281,88 |
| bladder | atorvastatin_UVA_Cohort | 0,504 | 0,831 | 0,306 | 0,00719458 | 471,88 |
| brain | atorvastatin_UVA_Cohort | 0,332 | 0,564 | 0,195 | 0,00005501 | 263,73 |
| breast | atorvastatin_UVA_Cohort | 0,224 | 0,388 | 0,130 | 0,00000014 | 165,29 |
| cancer | atorvastatin_UVA_Cohort | 0,473 | 0,510 | 0,438 | 0,00000000 | 10,89 |
| colorectal | atorvastatin_UVA_Cohort | 0,403 | 0,616 | 0,264 | 0,00003139 | 228,21 |
| esophagus | atorvastatin_UVA_Cohort | 1,065 | 1,774 | 0,639 | 0,82064966 | -7606,49 |
| kidney | atorvastatin_UVA_Cohort | 0,408 | 0,921 | 0,181 | 0,03051310 | 854,36 |
| liver | atorvastatin_UVA_Cohort | 0,367 | 0,893 | 0,151 | 0,02682121 | 861,19 |
| lung | atorvastatin_UVA_Cohort | 0,748 | 0,997 | 0,561 | 0,04740669 | 449,94 |
| ovaries | atorvastatin_UVA_Cohort | 0,249 | 0,502 | 0,124 | 0,00011013 | 308,51 |
| pancreas | atorvastatin_UVA_Cohort | 0,530 | 0,923 | 0,304 | 0,02483085 | 644,38 |
| prostate | atorvastatin_UVA_Cohort | 0,682 | 0,930 | 0,500 | 0,01553347 | 379,75 |
| seq77 | atorvastatin_UVA_Cohort | 0,339 | 0,465 | 0,246 | 0,00000000 | 97,52 |
| seq78 | atorvastatin_UVA_Cohort | 0,333 | 0,424 | 0,262 | 0,00000000 | 55,45 |
| seq79 | atorvastatin_UVA_Cohort | 0,426 | 0,532 | 0,341 | 0,00000000 | 68,96 |
| stomach | atorvastatin_UVA_Cohort | 0,100 | 0,714 | 0,014 | 0,02152952 | 823,82 |
| bladder | fluvastatin_charite_Cohort | 1,278 | 2,153 | 0,759 | 0,36236861 | -283,95 |
| brain | fluvastatin_charite_Cohort | 0,000 | nan | 0,000 | nan | 128,39 |
| breast | fluvastatin_charite_Cohort | 0,421 | 1,303 | 0,136 | 0,13359885 | 209,36 |
| cancer | fluvastatin_charite_Cohort | 0,768 | 0,892 | 0,661 | 0,00059087 | 20,41 |
| colorectal | fluvastatin_charite_Cohort | 0,611 | 1,132 | 0,329 | 0,11804412 | 135,82 |
| esophagus | fluvastatin_charite_Cohort | 1,698 | 3,781 | 0,762 | 0,19643705 | -350,44 |
| kidney | fluvastatin_charite_Cohort | 2,253 | 3,569 | 1,423 | 0,00056940 | -86,80 |
| liver | fluvastatin_charite_Cohort | 0,583 | 1,399 | 0,243 | 0,22906666 | 241,57 |
| lung | fluvastatin_charite_Cohort | 0,661 | 1,267 | 0,344 | 0,21501041 | 186,81 |
| ovaries | fluvastatin_charite_Cohort | 0,233 | 1,657 | 0,033 | 0,14516057 | 263,10 |
| pancreas | fluvastatin_charite_Cohort | 0,263 | 1,052 | 0,066 | 0,05823774 | 154,52 |
| prostate | fluvastatin_charite_Cohort | 1,194 | 1,975 | 0,722 | 0,49972728 | -356,47 |
| seq77 | fluvastatin_charite_Cohort | 0,597 | 0,957 | 0,373 | 0,03159736 | 75,69 |
| seq78 | fluvastatin_charite_Cohort | 0,619 | 0,927 | 0,413 | 0,01989581 | 61,27 |
| seq79 | fluvastatin_charite_Cohort | 0,832 | 1,216 | 0,569 | 0,34797885 | 166,13 |
| stomach | fluvastatin_charite_Cohort | 0,365 | 1,461 | 0,091 | 0,15520242 | 249,09 |
| bladder | fluvastatin_transatlantic_Cohort | 1,668 | 2,809 | 0,991 | 0,05384184 | -156,01 |
| brain | fluvastatin_transatlantic_Cohort | 0,000 | nan | 0,000 | nan | 144,04 |
| breast | fluvastatin_transatlantic_Cohort | 0,429 | 1,329 | 0,139 | 0,14202220 | 219,14 |

| | | | | | | |
|---|---|---|---|---|---|---|
| cancer | fluvastatin_transatlantic_Cohort | 0,822 | 0,955 | 0,708 | 0,01022126 | 28,73 |
| colorectal | fluvastatin_transatlantic_Cohort | 0,857 | 1,543 | 0,476 | 0,61949024 | 477,96 |
| esophagus | fluvastatin_transatlantic_Cohort | 2,048 | 4,559 | 0,920 | 0,07876138 | -284,63 |
| kidney | fluvastatin_transatlantic_Cohort | 3,121 | 4,943 | 1,971 | 0,00000172 | -71,85 |
| liver | fluvastatin_transatlantic_Cohort | 0,821 | 1,969 | 0,342 | 0,67176963 | 799,50 |
| lung | fluvastatin_transatlantic_Cohort | 0,778 | 1,492 | 0,406 | 0,45845290 | 340,63 |
| ovaries | fluvastatin_transatlantic_Cohort | 0,245 | 1,740 | 0,035 | 0,15870180 | 283,97 |
| pancreas | fluvastatin_transatlantic_Cohort | 0,338 | 1,349 | 0,085 | 0,12405946 | 223,08 |
| prostate | fluvastatin_transatlantic_Cohort | 1,397 | 2,309 | 0,845 | 0,19361733 | -206,09 |
| seq77 | fluvastatin_transatlantic_Cohort | 0,733 | 1,175 | 0,458 | 0,19762215 | 141,96 |
| seq78 | fluvastatin_transatlantic_Cohort | 0,711 | 1,065 | 0,475 | 0,09748340 | 94,03 |
| seq79 | fluvastatin_transatlantic_Cohort | 0,926 | 1,353 | 0,634 | 0,70411316 | 425,08 |
| stomach | fluvastatin_transatlantic_Cohort | 0,508 | 2,030 | 0,127 | 0,34351616 | 451,50 |
| bladder | fluvastatin_UVA_Cohort | 0,000 | nan | 0,000 | nan | 238,43 |
| brain | fluvastatin_UVA_Cohort | 0,000 | nan | 0,000 | nan | 183,16 |
| breast | fluvastatin_UVA_Cohort | 0,000 | nan | 0,000 | nan | 132,46 |
| cancer | fluvastatin_UVA_Cohort | 0,588 | 3,775 | 0,092 | 0,58714551 | 14,27 |
| colorectal | fluvastatin_UVA_Cohort | 13,954 | 89,721 | 2,170 | 0,00553781 | -10,77 |
| esophagus | fluvastatin_UVA_Cohort | 0,000 | nan | 0,000 | nan | 486,35 |
| kidney | fluvastatin_UVA_Cohort | 0,000 | nan | 0,000 | nan | 515,34 |
| liver | fluvastatin_UVA_Cohort | 0,000 | nan | 0,000 | nan | 561,09 |
| lung | fluvastatin_UVA_Cohort | 0,000 | nan | 0,000 | nan | 113,78 |
| ovaries | fluvastatin_UVA_Cohort | 0,000 | nan | 0,000 | nan | 240,78 |
| pancreas | fluvastatin_UVA_Cohort | 0,000 | nan | 0,000 | nan | 309,08 |
| prostate | fluvastatin_UVA_Cohort | 0,000 | nan | 0,000 | nan | 120,54 |
| seq77 | fluvastatin_UVA_Cohort | 0,000 | nan | 0,000 | nan | 66,13 |
| seq78 | fluvastatin_UVA_Cohort | 0,000 | nan | 0,000 | nan | 37,96 |
| seq79 | fluvastatin_UVA_Cohort | 0,000 | nan | 0,000 | nan | 40,44 |
| stomach | fluvastatin_UVA_Cohort | 0,000 | nan | 0,000 | nan | 770,74 |
| bladder | pravastatin_charite_Cohort | 1,820 | 2,757 | 1,202 | 0,00473027 | -96,32 |
| brain | pravastatin_charite_Cohort | 0,405 | 1,256 | 0,131 | 0,11696336 | 215,91 |
| breast | pravastatin_charite_Cohort | 0,637 | 1,530 | 0,265 | 0,31793719 | 334,39 |
| cancer | pravastatin_charite_Cohort | 1,003 | 1,132 | 0,889 | 0,96481003 | -1609,80 |
| colorectal | pravastatin_charite_Cohort | 0,499 | 0,957 | 0,260 | 0,03621028 | 105,48 |
| esophagus | pravastatin_charite_Cohort | 1,023 | 2,727 | 0,384 | 0,96709036 | -10659,15 |
| kidney | pravastatin_charite_Cohort | 1,484 | 2,552 | 0,863 | 0,15402595 | -224,75 |
| liver | pravastatin_charite_Cohort | 0,212 | 0,846 | 0,053 | 0,02793005 | 127,62 |
| lung | pravastatin_charite_Cohort | 1,000 | 1,655 | 0,605 | 1,00000000 | -261485,81 |
| ovaries | pravastatin_charite_Cohort | 1,487 | 3,120 | 0,708 | 0,29839227 | -415,50 |
| pancreas | pravastatin_charite_Cohort | 0,837 | 1,754 | 0,399 | 0,65047280 | 698,67 |
| prostate | pravastatin_charite_Cohort | 1,740 | 2,588 | 1,170 | 0,00626592 | -93,58 |
| seq77 | pravastatin_charite_Cohort | 1,078 | 1,501 | 0,774 | 0,66969261 | -392,78 |
| seq78 | pravastatin_charite_Cohort | 0,752 | 1,064 | 0,531 | 0,10778773 | 94,09 |
| seq79 | pravastatin_charite_Cohort | 0,871 | 1,239 | 0,612 | 0,45136894 | 216,04 |
| stomach | pravastatin_charite_Cohort | 0,665 | 1,772 | 0,250 | 0,42193321 | 472,88 |
| bladder | pravastatin_transatlantic_Cohort | 0,880 | 1,336 | 0,579 | 0,56038745 | 863,95 |
| brain | pravastatin_transatlantic_Cohort | 0,388 | 0,814 | 0,185 | 0,01220153 | 235,07 |
| breast | pravastatin_transatlantic_Cohort | 0,432 | 0,831 | 0,225 | 0,01175244 | 219,94 |
| cancer | pravastatin_transatlantic_Cohort | 0,738 | 0,810 | 0,672 | 0,00000000 | 19,46 |
| colorectal | pravastatin_transatlantic_Cohort | 0,419 | 0,684 | 0,257 | 0,00052834 | 117,27 |
| esophagus | pravastatin_transatlantic_Cohort | 0,571 | 1,374 | 0,237 | 0,21307558 | 691,86 |
| kidney | pravastatin_transatlantic_Cohort | 0,878 | 1,457 | 0,529 | 0,62727756 | 1247,07 |
| liver | pravastatin_transatlantic_Cohort | 0,165 | 0,512 | 0,053 | 0,00189729 | 171,16 |
| lung | pravastatin_transatlantic_Cohort | 0,844 | 1,214 | 0,587 | 0,36631804 | 484,91 |
| ovaries | pravastatin_transatlantic_Cohort | 0,908 | 1,642 | 0,502 | 0,76239373 | 2333,24 |
| pancreas | pravastatin_transatlantic_Cohort | 0,624 | 1,128 | 0,346 | 0,11769346 | 392,79 |
| prostate | pravastatin_transatlantic_Cohort | 1,357 | 1,829 | 1,007 | 0,04458478 | -230,04 |
| seq77 | pravastatin_transatlantic_Cohort | 0,550 | 0,755 | 0,401 | 0,00023361 | 84,04 |
| seq78 | pravastatin_transatlantic_Cohort | 0,561 | 0,730 | 0,430 | 0,00002267 | 61,62 |
| seq79 | pravastatin_transatlantic_Cohort | 0,614 | 0,806 | 0,467 | 0,00049152 | 81,38 |
| stomach | pravastatin_transatlantic_Cohort | 0,342 | 0,911 | 0,128 | 0,03183007 | 336,85 |
| bladder | pravastatin_UVA_Cohort | 0,000 | nan | 0,000 | nan | 236,13 |
| brain | pravastatin_UVA_Cohort | 0,444 | 1,187 | 0,166 | 0,10550477 | 328,90 |
| breast | pravastatin_UVA_Cohort | 0,320 | 0,853 | 0,120 | 0,02258455 | 193,37 |
| cancer | pravastatin_UVA_Cohort | 0,614 | 0,708 | 0,533 | 0,00000000 | 15,21 |
| colorectal | pravastatin_UVA_Cohort | 0,592 | 1,245 | 0,282 | 0,16713509 | 341,28 |
| esophagus | pravastatin_UVA_Cohort | 0,294 | 2,097 | 0,041 | 0,22464588 | 685,88 |
| kidney | pravastatin_UVA_Cohort | 0,627 | 2,524 | 0,156 | 0,52148463 | 1383,09 |
| liver | pravastatin_UVA_Cohort | 0,340 | 2,424 | 0,048 | 0,28456782 | 846,44 |
| lung | pravastatin_UVA_Cohort | 0,971 | 1,643 | 0,574 | 0,91974648 | 3959,03 |

| | | | | | | |
|---|---|---|---|---|---|---|
| ovaries | pravastatin_UVA_Cohort | 0,584 | 1,562 | 0,218 | 0,28807270 | 577,54 |
| pancreas | pravastatin_UVA_Cohort | 0,749 | 2,005 | 0,280 | 0,57670855 | 1226,12 |
| prostate | pravastatin_UVA_Cohort | 1,410 | 2,217 | 0,897 | 0,13683920 | -297,87 |
| seq77 | pravastatin_UVA_Cohort | 0,159 | 0,425 | 0,060 | 0,00025366 | 78,07 |
| seq78 | pravastatin_UVA_Cohort | 0,528 | 0,794 | 0,351 | 0,00221617 | 80,22 |
| seq79 | pravastatin_UVA_Cohort | 0,514 | 0,787 | 0,335 | 0,00230768 | 82,95 |
| stomach | pravastatin_UVA_Cohort | 0,000 | nan | 0,000 | nan | 763,31 |
| bladder | simvastatin_charite_Cohort | 1,867 | 2,063 | 1,690 | 0,00000000 | -99,74 |
| brain | simvastatin_charite_Cohort | 0,610 | 0,742 | 0,502 | 0,00000103 | 318,82 |
| breast | simvastatin_charite_Cohort | 0,586 | 0,710 | 0,483 | 0,00000009 | 282,78 |
| cancer | simvastatin_charite_Cohort | 0,945 | 0,972 | 0,920 | 0,00006386 | 86,05 |
| colorectal | simvastatin_charite_Cohort | 0,847 | 0,944 | 0,760 | 0,00273241 | 341,15 |
| esophagus | simvastatin_charite_Cohort | 1,152 | 1,413 | 0,938 | 0,17673512 | -1589,86 |
| kidney | simvastatin_charite_Cohort | 1,371 | 1,563 | 1,203 | 0,00000313 | -309,11 |
| liver | simvastatin_charite_Cohort | 0,714 | 0,839 | 0,609 | 0,00004441 | 341,90 |
| lung | simvastatin_charite_Cohort | 1,371 | 1,513 | 1,243 | 0,00000000 | -176,63 |
| ovaries | simvastatin_charite_Cohort | 0,494 | 0,644 | 0,378 | 0,00000033 | 376,29 |
| pancreas | simvastatin_charite_Cohort | 0,854 | 1,001 | 0,729 | 0,05065770 | 766,44 |
| prostate | simvastatin_charite_Cohort | 1,391 | 1,543 | 1,255 | 0,00000000 | -186,72 |
| seq77 | simvastatin_charite_Cohort | 0,879 | 0,952 | 0,812 | 0,00153184 | 249,95 |
| seq78 | simvastatin_charite_Cohort | 0,704 | 0,760 | 0,652 | 0,00000000 | 76,19 |
| seq79 | simvastatin_charite_Cohort | 0,785 | 0,851 | 0,725 | 0,00000001 | 127,11 |
| stomach | simvastatin_charite_Cohort | 0,891 | 1,071 | 0,741 | 0,22131364 | 1428,70 |
| bladder | simvastatin_transatlantic_Cohort | 2,163 | 2,383 | 1,962 | 0,00000000 | -98,22 |
| brain | simvastatin_transatlantic_Cohort | 0,656 | 0,788 | 0,545 | 0,00000943 | 409,97 |
| breast | simvastatin_transatlantic_Cohort | 0,610 | 0,729 | 0,511 | 0,00000008 | 314,16 |
| cancer | simvastatin_transatlantic_Cohort | 0,964 | 0,989 | 0,939 | 0,00563314 | 141,33 |
| colorectal | simvastatin_transatlantic_Cohort | 0,994 | 1,104 | 0,894 | 0,91817358 | 10726,16 |
| esophagus | simvastatin_transatlantic_Cohort | 1,308 | 1,592 | 1,075 | 0,00736776 | -971,41 |
| kidney | simvastatin_transatlantic_Cohort | 1,686 | 1,919 | 1,481 | 0,00000000 | -237,41 |
| liver | simvastatin_transatlantic_Cohort | 0,909 | 1,065 | 0,775 | 0,24182110 | 1558,71 |
| lung | simvastatin_transatlantic_Cohort | 1,509 | 1,658 | 1,374 | 0,00000000 | -154,04 |
| ovaries | simvastatin_transatlantic_Cohort | 0,519 | 0,667 | 0,405 | 0,00000040 | 429,90 |
| pancreas | simvastatin_transatlantic_Cohort | 1,018 | 1,188 | 0,873 | 0,83178373 | -8103,83 |
| prostate | simvastatin_transatlantic_Cohort | 1,493 | 1,648 | 1,353 | 0,00000000 | -174,64 |
| seq77 | simvastatin_transatlantic_Cohort | 1,001 | 1,082 | 0,926 | 0,98185959 | -42610,79 |
| seq78 | simvastatin_transatlantic_Cohort | 0,769 | 0,828 | 0,715 | 0,00000000 | 115,64 |
| seq79 | simvastatin_transatlantic_Cohort | 0,829 | 0,895 | 0,768 | 0,00000216 | 181,96 |
| stomach | simvastatin_transatlantic_Cohort | 1,136 | 1,361 | 0,948 | 0,16764596 | -1648,44 |
| bladder | simvastatin_UVA_Cohort | 0,409 | 0,915 | 0,183 | 0,02919174 | 399,30 |
| brain | simvastatin_UVA_Cohort | 0,523 | 0,977 | 0,280 | 0,04169286 | 379,88 |
| breast | simvastatin_UVA_Cohort | 0,647 | 1,046 | 0,401 | 0,07466324 | 373,91 |
| cancer | simvastatin_UVA_Cohort | 0,661 | 0,726 | 0,602 | 0,00000000 | 17,25 |
| colorectal | simvastatin_UVA_Cohort | 0,477 | 0,843 | 0,270 | 0,01078588 | 263,11 |
| esophagus | simvastatin_UVA_Cohort | 0,850 | 1,915 | 0,377 | 0,70823554 | 3270,60 |
| kidney | simvastatin_UVA_Cohort | 0,000 | nan | 0,000 | nan | 503,72 |
| liver | simvastatin_UVA_Cohort | 0,159 | 1,138 | 0,022 | 0,06734275 | 656,30 |
| lung | simvastatin_UVA_Cohort | 0,961 | 1,390 | 0,665 | 0,84344230 | 2953,97 |
| ovaries | simvastatin_UVA_Cohort | 0,483 | 1,020 | 0,229 | 0,05578103 | 462,48 |
| pancreas | simvastatin_UVA_Cohort | 0,623 | 1,317 | 0,295 | 0,21687114 | 816,98 |
| prostate | simvastatin_UVA_Cohort | 0,766 | 1,169 | 0,502 | 0,21825597 | 515,81 |
| seq77 | simvastatin_UVA_Cohort | 0,530 | 0,769 | 0,365 | 0,00088099 | 139,39 |
| seq78 | simvastatin_UVA_Cohort | 0,521 | 0,692 | 0,393 | 0,00000806 | 78,65 |
| seq79 | simvastatin_UVA_Cohort | 0,521 | 0,698 | 0,388 | 0,00001667 | 83,65 |
| stomach | simvastatin_UVA_Cohort | 0,664 | 2,085 | 0,211 | 0,49327588 | 2276,65 |
| bladder | statins_charite_Cohort | 1,831 | 2,007 | 1,671 | 0,00000000 | -106,95 |
| brain | statins_charite_Cohort | 0,542 | 0,648 | 0,454 | 0,00000000 | 264,09 |
| breast | statins_charite_Cohort | 0,531 | 0,632 | 0,446 | 0,00000000 | 243,35 |
| cancer | statins_charite_Cohort | 0,865 | 0,887 | 0,844 | 0,00000000 | 34,35 |
| cancersubentities | statins_charite_Cohort | 0,935 | 0,967 | 0,905 | 0,00008053 | 119,31 |
| colorectal | statins_charite_Cohort | 0,753 | 0,832 | 0,682 | 0,00000004 | 207,62 |
| esophagus | statins_charite_Cohort | 1,019 | 1,232 | 0,843 | 0,85628348 | -12446,38 |
| kidney | statins_charite_Cohort | 1,343 | 1,512 | 1,194 | 0,00000140 | -337,59 |
| liver | statins_charite_Cohort | 0,601 | 0,698 | 0,517 | 0,00000000 | 237,39 |
| lung | statins_charite_Cohort | 1,235 | 1,352 | 1,127 | 0,00000711 | -276,08 |
| ovaries | statins_charite_Cohort | 0,441 | 0,562 | 0,347 | 0,00000000 | 329,88 |
| pancreas | statins_charite_Cohort | 0,764 | 0,883 | 0,660 | 0,00031403 | 462,77 |
| prostate | statins_charite_Cohort | 1,329 | 1,460 | 1,210 | 0,00000001 | -223,03 |
| seq77 | statins_charite_Cohort | 0,787 | 0,847 | 0,731 | 0,00000000 | 138,90 |
| seq78 | statins_charite_Cohort | 0,622 | 0,667 | 0,580 | 0,00000000 | 58,12 |

| | | | | | | |
|---|---|---|---|---|---|---|
| seq79 | statins_charite_Cohort | 0,708 | 0,762 | 0,658 | 0,00000000 | 91,47 |
| stomach | statins_charite_Cohort | 0,743 | 0,884 | 0,625 | 0,00082432 | 588,99 |
| bladder | statins_transatlantic_Cohort | 1,723 | 1,882 | 1,578 | 0,00000000 | -159,82 |
| brain | statins_transatlantic_Cohort | 0,517 | 0,607 | 0,441 | 0,00000000 | 282,62 |
| breast | statins_transatlantic_Cohort | 0,480 | 0,560 | 0,412 | 0,00000000 | 227,40 |
| cancer | statins_transatlantic_Cohort | 0,797 | 0,815 | 0,779 | 0,00000000 | 24,53 |
| colorectal | statins_transatlantic_Cohort | 0,748 | 0,822 | 0,681 | 0,00000000 | 264,99 |
| esophagus | statins_transatlantic_Cohort | 1,085 | 1,287 | 0,914 | 0,35586601 | -3505,14 |
| kidney | statins_transatlantic_Cohort | 1,346 | 1,511 | 1,199 | 0,00000071 | -470,86 |
| liver | statins_transatlantic_Cohort | 0,627 | 0,726 | 0,541 | 0,00000000 | 367,74 |
| lung | statins_transatlantic_Cohort | 1,206 | 1,310 | 1,109 | 0,00001308 | -377,70 |
| ovaries | statins_transatlantic_Cohort | 0,426 | 0,527 | 0,345 | 0,00000000 | 345,91 |
| pancreas | statins_transatlantic_Cohort | 0,781 | 0,895 | 0,681 | 0,00042104 | 655,76 |
| prostate | statins_transatlantic_Cohort | 1,274 | 1,388 | 1,169 | 0,00000006 | -316,16 |
| seq77 | statins_transatlantic_Cohort | 0,751 | 0,805 | 0,700 | 0,00000000 | 148,13 |
| seq78 | statins_transatlantic_Cohort | 0,601 | 0,641 | 0,564 | 0,00000000 | 64,71 |
| seq79 | statins_transatlantic_Cohort | 0,672 | 0,718 | 0,629 | 0,00000000 | 92,31 |
| stomach | statins_transatlantic_Cohort | 0,761 | 0,901 | 0,643 | 0,00155665 | 901,38 |
| bladder | statins_UVA_Cohort | 0,433 | 0,649 | 0,289 | 0,00005825 | 403,96 |
| brain | statins_UVA_Cohort | 0,381 | 0,554 | 0,262 | 0,00000066 | 280,26 |
| breast | statins_UVA_Cohort | 0,342 | 0,478 | 0,245 | 0,00000000 | 190,74 |
| cancer | statins_UVA_Cohort | 0,554 | 0,585 | 0,526 | 0,00000000 | 12,72 |
| cancersubentities | statins_UVA_Cohort | 0,528 | 0,574 | 0,486 | 0,00000000 | 25,96 |
| colorectal | statins_UVA_Cohort | 0,434 | 0,590 | 0,319 | 0,00000017 | 235,43 |
| esophagus | statins_UVA_Cohort | 1,113 | 1,649 | 0,751 | 0,60591437 | -4490,73 |
| kidney | statins_UVA_Cohort | 0,336 | 0,655 | 0,172 | 0,00143681 | 741,44 |
| liver | statins_UVA_Cohort | 0,280 | 0,597 | 0,132 | 0,00098864 | 734,53 |
| lung | statins_UVA_Cohort | 0,863 | 1,063 | 0,700 | 0,16756369 | 837,78 |
| ovaries | statins_UVA_Cohort | 0,361 | 0,560 | 0,233 | 0,00000682 | 355,42 |
| pancreas | statins_UVA_Cohort | 0,566 | 0,851 | 0,376 | 0,00633191 | 689,46 |
| prostate | statins_UVA_Cohort | 0,856 | 1,063 | 0,689 | 0,16044605 | 854,23 |
| seq77 | statins_UVA_Cohort | 0,367 | 0,462 | 0,292 | 0,00000000 | 99,68 |
| seq78 | statins_UVA_Cohort | 0,426 | 0,501 | 0,363 | 0,00000000 | 63,37 |
| seq79 | statins_UVA_Cohort | 0,477 | 0,559 | 0,407 | 0,00000000 | 74,40 |
| stomach | statins_UVA_Cohort | 0,273 | 0,669 | 0,112 | 0,00445089 | 994,24 |

| | | | | 1:1 matched Cohort MEAN Odds-Ratio | | | | |
|---|---|---|---|---|---|---|---|---|
| Diagnose | Drug | NbDrug | NbDiag | NbDiag/Drug | Odds Ratio | Conf-Low | Conf-Up | P-Value |
| bladder | atorvastatin_charite_Cohort | 199 | 2100 | 75 | 0,574 | 0,428 | 0,77 | 0,000 |
| brain | atorvastatin_charite_Cohort | 66 | 1296 | 20 | 0,424 | 0,249 | 0,72 | 0,002 |
| breast | atorvastatin_charite_Cohort | 70 | 1373 | 19 | 0,374 | 0,219 | 0,64 | 0,000 |
| cancer | atorvastatin_charite_Cohort | 2727 | 36203 | 711 | 0,341 | 0,312 | 0,37 | 0,000 |
| colorectal | atorvastatin_charite_Cohort | 216 | 3151 | 50 | 0,286 | 0,208 | 0,39 | 0,000 |
| esophagus | atorvastatin_charite_Cohort | 50 | 678 | 7 | 0,170 | 0,075 | 0,38 | 0,000 |
| kidney | atorvastatin_charite_Cohort | 141 | 1530 | 45 | 0,512 | 0,354 | 0,74 | 0,000 |
| liver | atorvastatin_charite_Cohort | 110 | 1651 | 17 | 0,179 | 0,106 | 0,30 | 0,000 |
| lung | atorvastatin_charite_Cohort | 211 | 2630 | 69 | 0,457 | 0,341 | 0,61 | 0,000 |
| ovaries | atorvastatin_charite_Cohort | 35 | 828 | 6 | 0,203 | 0,083 | 0,49 | 0,000 |
| pancreas | atorvastatin_charite_Cohort | 115 | 1464 | 28 | 0,371 | 0,238 | 0,58 | 0,000 |
| prostate | atorvastatin_charite_Cohort | 239 | 2409 | 59 | 0,328 | 0,242 | 0,44 | 0,000 |
| seq77 | atorvastatin_charite_Cohort | 317 | 5507 | 73 | 0,261 | 0,201 | 0,34 | 0,000 |
| seq78 | atorvastatin_charite_Cohort | 430 | 7200 | 73 | 0,203 | 0,158 | 0,26 | 0,000 |
| seq79 | atorvastatin_charite_Cohort | 376 | 6024 | 81 | 0,257 | 0,200 | 0,33 | 0,000 |
| stomach | atorvastatin_charite_Cohort | 66 | 1057 | 12 | 0,212 | 0,113 | 0,40 | 0,000 |
| bladder | atorvastatin_transatlantic_Cohort | 288 | 2489 | 88 | 0,397 | 0,308 | 0,51 | 0,000 |
| brain | atorvastatin_transatlantic_Cohort | 133 | 1815 | 31 | 0,332 | 0,220 | 0,50 | 0,000 |
| breast | atorvastatin_transatlantic_Cohort | 139 | 2081 | 29 | 0,257 | 0,170 | 0,39 | 0,000 |
| cancer | atorvastatin_transatlantic_Cohort | 5125 | 52882 | 1235 | 0,299 | 0,280 | 0,32 | 0,000 |
| colorectal | atorvastatin_transatlantic_Cohort | 344 | 3827 | 71 | 0,235 | 0,181 | 0,31 | 0,000 |
| esophagus | atorvastatin_transatlantic_Cohort | 86 | 870 | 20 | 0,281 | 0,169 | 0,47 | 0,000 |
| kidney | atorvastatin_transatlantic_Cohort | 178 | 1708 | 50 | 0,380 | 0,272 | 0,53 | 0,000 |
| liver | atorvastatin_transatlantic_Cohort | 157 | 1822 | 22 | 0,150 | 0,095 | 0,24 | 0,000 |
| lung | atorvastatin_transatlantic_Cohort | 359 | 3443 | 110 | 0,406 | 0,323 | 0,51 | 0,000 |
| ovaries | atorvastatin_transatlantic_Cohort | 82 | 1219 | 11 | 0,170 | 0,089 | 0,33 | 0,000 |
| pancreas | atorvastatin_transatlantic_Cohort | 168 | 1766 | 40 | 0,311 | 0,216 | 0,45 | 0,000 |
| prostate | atorvastatin_transatlantic_Cohort | 390 | 3178 | 98 | 0,310 | 0,245 | 0,39 | 0,000 |
| seq77 | atorvastatin_transatlantic_Cohort | 557 | 6925 | 106 | 0,218 | 0,176 | 0,27 | 0,000 |
| seq78 | atorvastatin_transatlantic_Cohort | 747 | 9688 | 130 | 0,188 | 0,156 | 0,23 | 0,000 |
| seq79 | atorvastatin_transatlantic_Cohort | 719 | 8363 | 148 | 0,244 | 0,203 | 0,29 | 0,000 |
| stomach | atorvastatin_transatlantic_Cohort | 77 | 1176 | 13 | 0,153 | 0,085 | 0,28 | 0,000 |
| bladder | atorvastatin_UVA_Cohort | 67 | 389 | 13 | 0,250 | 0,133 | 0,47 | 0,000 |
| brain | atorvastatin_UVA_Cohort | 51 | 519 | 11 | 0,263 | 0,133 | 0,52 | 0,000 |
| breast | atorvastatin_UVA_Cohort | 63 | 708 | 10 | 0,187 | 0,094 | 0,37 | 0,000 |
| cancer | atorvastatin_UVA_Cohort | 2328 | 16679 | 524 | 0,281 | 0,254 | 0,31 | 0,000 |
| colorectal | atorvastatin_UVA_Cohort | 100 | 676 | 21 | 0,264 | 0,160 | 0,44 | 0,000 |
| esophagus | atorvastatin_UVA_Cohort | 34 | 192 | 13 | 0,585 | 0,282 | 1,22 | 0,151 |
| kidney | atorvastatin_UVA_Cohort | 20 | 178 | 5 | 0,274 | 0,098 | 0,77 | 0,014 |
| liver | atorvastatin_UVA_Cohort | 26 | 171 | 5 | 0,247 | 0,089 | 0,68 | 0,007 |
| lung | atorvastatin_UVA_Cohort | 135 | 813 | 41 | 0,409 | 0,279 | 0,60 | 0,000 |
| ovaries | atorvastatin_UVA_Cohort | 34 | 391 | 5 | 0,175 | 0,066 | 0,46 | 0,000 |
| pancreas | atorvastatin_UVA_Cohort | 47 | 302 | 12 | 0,345 | 0,174 | 0,68 | 0,002 |
| prostate | atorvastatin_UVA_Cohort | 136 | 769 | 39 | 0,340 | 0,232 | 0,50 | 0,000 |
| seq77 | atorvastatin_UVA_Cohort | 166 | 1418 | 33 | 0,227 | 0,154 | 0,33 | 0,000 |
| seq78 | atorvastatin_UVA_Cohort | 266 | 2488 | 57 | 0,210 | 0,157 | 0,28 | 0,000 |
| seq79 | atorvastatin_UVA_Cohort | 296 | 2339 | 67 | 0,256 | 0,194 | 0,34 | 0,000 |
| stomach | atorvastatin_UVA_Cohort | 10 | 119 | 1 | 0,080 | 0,010 | 0,63 | 0,017 |
| bladder | fluvastatin_charite_Cohort | 31 | 2119 | 12 | 0,810 | 0,373 | 1,76 | 0,607 |
| brain | fluvastatin_charite_Cohort | 4 | 1295 | 0 | 0,000 | 0,000 | nan | nan |
| breast | fluvastatin_charite_Cohort | 8 | 1370 | 2 | 0,366 | 0,070 | 1,95 | 0,239 |
| cancer | fluvastatin_charite_Cohort | 354 | 36199 | 118 | 0,483 | 0,387 | 0,60 | 0,000 |
| colorectal | fluvastatin_charite_Cohort | 28 | 3162 | 8 | 0,383 | 0,168 | 0,87 | 0,022 |
| esophagus | fluvastatin_charite_Cohort | 7 | 683 | 5 | 1,143 | 0,299 | 4,53 | 0,858 |
| kidney | fluvastatin_charite_Cohort | 19 | 1535 | 14 | 1,392 | 0,609 | 3,21 | 0,443 |
| liver | fluvastatin_charite_Cohort | 16 | 1656 | 4 | 0,416 | 0,123 | 1,43 | 0,162 |
| lung | fluvastatin_charite_Cohort | 27 | 2633 | 8 | 0,446 | 0,192 | 1,04 | 0,060 |
| ovaries | fluvastatin_charite_Cohort | 4 | 825 | 1 | inf | 0,030 | inf | nan |
| pancreas | fluvastatin_charite_Cohort | 12 | 1462 | 2 | 0,233 | 0,048 | 1,14 | 0,071 |
| prostate | fluvastatin_charite_Cohort | 31 | 2420 | 12 | 0,606 | 0,292 | 1,26 | 0,180 |
| seq77 | fluvastatin_charite_Cohort | 48 | 5530 | 10 | 0,280 | 0,138 | 0,57 | 0,000 |
| seq78 | fluvastatin_charite_Cohort | 73 | 7220 | 18 | 0,370 | 0,215 | 0,64 | 0,000 |
| seq79 | fluvastatin_charite_Cohort | 57 | 6028 | 18 | 0,446 | 0,255 | 0,78 | 0,005 |
| stomach | fluvastatin_charite_Cohort | 11 | 1050 | 1 | 0,183 | 0,021 | 1,61 | 0,125 |
| bladder | fluvastatin_transatlantic_Cohort | 28 | 2517 | 12 | 0,855 | 0,392 | 1,87 | 0,707 |
| brain | fluvastatin_transatlantic_Cohort | 9 | 1813 | 0 | 0,000 | 0,000 | nan | nan |
| breast | fluvastatin_transatlantic_Cohort | 8 | 2087 | 2 | 0,329 | 0,065 | 1,67 | 0,180 |
| cancer | fluvastatin_transatlantic_Cohort | 361 | 53012 | 119 | 0,514 | 0,412 | 0,64 | 0,000 |
| colorectal | fluvastatin_transatlantic_Cohort | 25 | 3844 | 9 | 0,492 | 0,220 | 1,10 | 0,084 |
| esophagus | fluvastatin_transatlantic_Cohort | 11 | 878 | 5 | inf | 0,303 | inf | nan |
| kidney | fluvastatin_transatlantic_Cohort | 23 | 1719 | 14 | 1,934 | 0,749 | 5,25 | 0,185 |
| liver | fluvastatin_transatlantic_Cohort | 17 | 1825 | 4 | 0,445 | 0,136 | 1,46 | 0,183 |
| lung | fluvastatin_transatlantic_Cohort | 24 | 3469 | 8 | 0,513 | 0,216 | 1,22 | 0,132 |
| ovaries | fluvastatin_transatlantic_Cohort | 6 | 1219 | 1 | inf | 0,032 | inf | nan |

Supplementary Table 3
1:1 matched Cohort
Mean Odds-Ratio

| | | | | | | | | |
|---|---|---|---|---|---|---|---|---|
| pancreas | fluvastatin_transatlantic_Cohort | 11 | 1769 | 2 | 0,287 | 0,058 | 1,42 | 0,126 |
| prostate | fluvastatin_transatlantic_Cohort | 31 | 3208 | 12 | 0,557 | 0,273 | 1,14 | 0,107 |
| seq77 | fluvastatin_transatlantic_Cohort | 36 | 6974 | 10 | 0,321 | 0,157 | 0,66 | 0,002 |
| seq78 | fluvastatin_transatlantic_Cohort | 54 | 9744 | 18 | 0,443 | 0,254 | 0,77 | 0,004 |
| seq79 | fluvastatin_transatlantic_Cohort | 53 | 8395 | 18 | 0,475 | 0,270 | 0,84 | 0,010 |
| stomach | fluvastatin_transatlantic_Cohort | 12 | 1173 | 1 | 0,206 | 0,023 | 1,89 | 0,161 |
| bladder | fluvastatin_UVA_Cohort | 0 | 398 | 0 | nan | nan | nan | nan |
| brain | fluvastatin_UVA_Cohort | 0 | 518 | 0 | 0,000 | 0,000 | nan | nan |
| breast | fluvastatin_UVA_Cohort | 0 | 717 | 0 | 0,000 | 0,000 | nan | nan |
| cancer | fluvastatin_UVA_Cohort | 4 | 16813 | 1 | 0,476 | 0,041 | 5,92 | 0,570 |
| colorectal | fluvastatin_UVA_Cohort | 1 | 682 | 1 | inf | 0,062 | inf | nan |
| esophagus | fluvastatin_UVA_Cohort | 0 | 195 | 0 | nan | nan | nan | nan |
| kidney | fluvastatin_UVA_Cohort | 0 | 184 | 0 | 0,000 | 0,000 | nan | nan |
| liver | fluvastatin_UVA_Cohort | 0 | 169 | 0 | 0,000 | 0,000 | nan | nan |
| lung | fluvastatin_UVA_Cohort | 0 | 836 | 0 | 0,000 | 0,000 | nan | nan |
| ovaries | fluvastatin_UVA_Cohort | 0 | 394 | 0 | 0,000 | 0,000 | nan | nan |
| pancreas | fluvastatin_UVA_Cohort | 0 | 307 | 0 | 0,000 | 0,000 | nan | nan |
| prostate | fluvastatin_UVA_Cohort | 0 | 788 | 0 | 0,000 | 0,000 | nan | nan |
| seq77 | fluvastatin_UVA_Cohort | 0 | 1444 | 0 | 0,000 | 0,000 | nan | nan |
| seq78 | fluvastatin_UVA_Cohort | 0 | 2524 | 0 | 0,000 | 0,000 | nan | nan |
| seq79 | fluvastatin_UVA_Cohort | 0 | 2367 | 0 | 0,000 | 0,000 | nan | nan |
| stomach | fluvastatin_UVA_Cohort | 0 | 123 | 0 | 0,000 | 0,000 | nan | nan |
| bladder | pravastatin_charite_Cohort | 37 | 2121 | 18 | 1,000 | 0,516 | 1,94 | 1,000 |
| brain | pravastatin_charite_Cohort | 7 | 1294 | 2 | 0,335 | 0,065 | 1,73 | 0,193 |
| breast | pravastatin_charite_Cohort | 9 | 1373 | 5 | 0,699 | 0,217 | 2,26 | 0,561 |
| cancer | pravastatin_charite_Cohort | 461 | 36213 | 178 | 0,662 | 0,547 | 0,80 | 0,000 |
| colorectal | pravastatin_charite_Cohort | 34 | 3157 | 6 | 0,264 | 0,106 | 0,66 | 0,004 |
| esophagus | pravastatin_charite_Cohort | 8 | 684 | 3 | 0,652 | 0,138 | 3,43 | 0,614 |
| kidney | pravastatin_charite_Cohort | 18 | 1535 | 11 | 1,051 | 0,440 | 2,53 | 0,918 |
| liver | pravastatin_charite_Cohort | 15 | 1652 | 1 | 0,069 | 0,009 | 0,53 | 0,010 |
| lung | pravastatin_charite_Cohort | 43 | 2631 | 12 | 0,577 | 0,281 | 1,19 | 0,134 |
| ovaries | pravastatin_charite_Cohort | 12 | 826 | 6 | 1,282 | 0,367 | 4,58 | 0,713 |
| pancreas | pravastatin_charite_Cohort | 13 | 1462 | 5 | 0,481 | 0,162 | 1,43 | 0,189 |
| prostate | pravastatin_charite_Cohort | 53 | 2423 | 23 | 0,934 | 0,526 | 1,66 | 0,827 |
| seq77 | pravastatin_charite_Cohort | 68 | 5526 | 24 | 0,594 | 0,357 | 0,99 | 0,045 |
| seq78 | pravastatin_charite_Cohort | 80 | 7215 | 22 | 0,432 | 0,261 | 0,71 | 0,001 |
| seq79 | pravastatin_charite_Cohort | 57 | 6023 | 18 | 0,417 | 0,240 | 0,73 | 0,002 |
| stomach | pravastatin_charite_Cohort | 12 | 1053 | 4 | 0,589 | 0,165 | 2,12 | 0,424 |
| bladder | pravastatin_transatlantic_Cohort | 52 | 2516 | 18 | 0,459 | 0,261 | 0,81 | 0,007 |
| brain | pravastatin_transatlantic_Cohort | 29 | 1812 | 6 | 0,363 | 0,140 | 0,94 | 0,037 |
| breast | pravastatin_transatlantic_Cohort | 25 | 2092 | 9 | 0,438 | 0,198 | 0,97 | 0,041 |
| cancer | pravastatin_transatlantic_Cohort | 1032 | 52982 | 331 | 0,475 | 0,416 | 0,54 | 0,000 |
| colorectal | pravastatin_transatlantic_Cohort | 60 | 3836 | 12 | 0,231 | 0,123 | 0,44 | 0,000 |
| esophagus | pravastatin_transatlantic_Cohort | 19 | 879 | 4 | 0,382 | 0,117 | 1,25 | 0,111 |
| kidney | pravastatin_transatlantic_Cohort | 37 | 1717 | 13 | 0,585 | 0,292 | 1,17 | 0,130 |
| liver | pravastatin_transatlantic_Cohort | 23 | 1821 | 2 | 0,080 | 0,019 | 0,34 | 0,001 |
| lung | pravastatin_transatlantic_Cohort | 70 | 3459 | 25 | 0,544 | 0,333 | 0,89 | 0,015 |
| ovaries | pravastatin_transatlantic_Cohort | 18 | 1219 | 9 | 0,796 | 0,326 | 1,95 | 0,630 |
| pancreas | pravastatin_transatlantic_Cohort | 32 | 1768 | 8 | 0,330 | 0,147 | 0,74 | 0,007 |
| prostate | pravastatin_transatlantic_Cohort | 88 | 3206 | 41 | 0,827 | 0,545 | 1,26 | 0,379 |
| seq77 | pravastatin_transatlantic_Cohort | 113 | 6965 | 27 | 0,322 | 0,208 | 0,50 | 0,000 |
| seq78 | pravastatin_transatlantic_Cohort | 132 | 9722 | 40 | 0,325 | 0,227 | 0,47 | 0,000 |
| seq79 | pravastatin_transatlantic_Cohort | 144 | 8379 | 35 | 0,316 | 0,215 | 0,46 | 0,000 |
| stomach | pravastatin_transatlantic_Cohort | 12 | 1175 | 4 | 0,294 | 0,095 | 0,91 | 0,033 |
| bladder | pravastatin_UVA_Cohort | 14 | 395 | 0 | 0,000 | 0,000 | nan | nan |
| brain | pravastatin_UVA_Cohort | 12 | 518 | 4 | 0,435 | 0,131 | 1,46 | 0,176 |
| breast | pravastatin_UVA_Cohort | 18 | 719 | 4 | 0,359 | 0,112 | 1,15 | 0,085 |
| cancer | pravastatin_UVA_Cohort | 550 | 16769 | 153 | 0,390 | 0,323 | 0,47 | 0,000 |
| colorectal | pravastatin_UVA_Cohort | 16 | 679 | 6 | 0,415 | 0,158 | 1,10 | 0,075 |
| esophagus | pravastatin_UVA_Cohort | 7 | 195 | 1 | 0,242 | 0,025 | 2,47 | 0,228 |
| kidney | pravastatin_UVA_Cohort | 7 | 182 | 2 | 0,528 | 0,087 | 3,40 | 0,505 |
| liver | pravastatin_UVA_Cohort | 5 | 169 | 1 | 0,279 | 0,029 | 2,73 | 0,274 |
| lung | pravastatin_UVA_Cohort | 34 | 828 | 13 | 0,653 | 0,320 | 1,33 | 0,244 |
| ovaries | pravastatin_UVA_Cohort | 14 | 393 | 3 | 0,423 | 0,103 | 1,78 | 0,239 |
| pancreas | pravastatin_UVA_Cohort | 15 | 306 | 3 | 0,412 | 0,104 | 1,66 | 0,211 |
| prostate | pravastatin_UVA_Cohort | 40 | 783 | 18 | 0,862 | 0,452 | 1,64 | 0,665 |
| seq77 | pravastatin_UVA_Cohort | 34 | 1439 | 3 | 0,087 | 0,027 | 0,29 | 0,000 |
| seq78 | pravastatin_UVA_Cohort | 81 | 2507 | 18 | 0,321 | 0,188 | 0,55 | 0,000 |
| seq79 | pravastatin_UVA_Cohort | 71 | 2356 | 17 | 0,309 | 0,178 | 0,53 | 0,000 |
| stomach | pravastatin_UVA_Cohort | 4 | 122 | 0 | 0,000 | 0,000 | nan | nan |
| bladder | simvastatin_charite_Cohort | 899 | 2105 | 431 | 0,930 | 0,802 | 1,08 | 0,341 |
| brain | simvastatin_charite_Cohort | 248 | 1300 | 104 | 0,665 | 0,511 | 0,87 | 0,002 |
| breast | simvastatin_charite_Cohort | 282 | 1366 | 102 | 0,515 | 0,399 | 0,66 | 0,000 |
| cancer | simvastatin_charite_Cohort | 10668 | 36506 | 4257 | 0,610 | 0,585 | 0,64 | 0,000 |
| colorectal | simvastatin_charite_Cohort | 862 | 3139 | 319 | 0,509 | 0,439 | 0,59 | 0,000 |
| esophagus | simvastatin_charite_Cohort | 210 | 704 | 98 | 0,734 | 0,551 | 0,98 | 0,035 |
| kidney | simvastatin_charite_Cohort | 541 | 1523 | 243 | 0,828 | 0,686 | 1,00 | 0,049 |

Supplementary Table 3
1:1 matched Cohort
Mean Odds-Ratio

| | | | | | | | | |
|---|---|---|---|---|---|---|---|---|
| liver | simvastatin_charite_Cohort | 466 | 1646 | 140 | 0,409 | 0,331 | 0,51 | 0,000 |
| lung | simvastatin_charite_Cohort | 897 | 2646 | 415 | 0,807 | 0,700 | 0,93 | 0,003 |
| ovaries | simvastatin_charite_Cohort | 165 | 833 | 53 | 0,461 | 0,327 | 0,65 | 0,000 |
| pancreas | simvastatin_charite_Cohort | 396 | 1473 | 155 | 0,563 | 0,454 | 0,70 | 0,000 |
| prostate | simvastatin_charite_Cohort | 936 | 2401 | 389 | 0,627 | 0,543 | 0,72 | 0,000 |
| seq77 | simvastatin_charite_Cohort | 1385 | 5374 | 510 | 0,541 | 0,481 | 0,61 | 0,000 |
| seq78 | simvastatin_charite_Cohort | 1782 | 7141 | 576 | 0,444 | 0,400 | 0,49 | 0,000 |
| seq79 | simvastatin_charite_Cohort | 1586 | 5961 | 540 | 0,486 | 0,435 | 0,54 | 0,000 |
| stomach | simvastatin_charite_Cohort | 293 | 1059 | 116 | 0,611 | 0,475 | 0,79 | 0,000 |
| bladder | simvastatin_transatlantic_Cohort | 866 | 2502 | 437 | 1,063 | 0,917 | 1,23 | 0,424 |
| brain | simvastatin_transatlantic_Cohort | 274 | 1818 | 113 | 0,665 | 0,519 | 0,85 | 0,001 |
| breast | simvastatin_transatlantic_Cohort | 331 | 2077 | 116 | 0,540 | 0,427 | 0,69 | 0,000 |
| cancer | simvastatin_transatlantic_Cohort | 11555 | 53267 | 4608 | 0,634 | 0,610 | 0,66 | 0,000 |
| colorectal | simvastatin_transatlantic_Cohort | 849 | 3816 | 329 | 0,597 | 0,516 | 0,69 | 0,000 |
| esophagus | simvastatin_transatlantic_Cohort | 231 | 897 | 104 | 0,782 | 0,593 | 1,03 | 0,082 |
| kidney | simvastatin_transatlantic_Cohort | 475 | 1705 | 243 | 1,020 | 0,841 | 1,24 | 0,851 |
| liver | simvastatin_transatlantic_Cohort | 396 | 1814 | 141 | 0,501 | 0,404 | 0,62 | 0,000 |
| lung | simvastatin_transatlantic_Cohort | 929 | 3472 | 442 | 0,896 | 0,780 | 1,03 | 0,119 |
| ovaries | simvastatin_transatlantic_Cohort | 168 | 1224 | 59 | 0,462 | 0,335 | 0,64 | 0,000 |
| pancreas | simvastatin_transatlantic_Cohort | 382 | 1778 | 161 | 0,646 | 0,523 | 0,80 | 0,000 |
| prostate | simvastatin_transatlantic_Cohort | 1010 | 3183 | 407 | 0,662 | 0,577 | 0,76 | 0,000 |
| seq77 | simvastatin_transatlantic_Cohort | 1402 | 6807 | 534 | 0,601 | 0,536 | 0,67 | 0,000 |
| seq78 | simvastatin_transatlantic_Cohort | 1843 | 9644 | 616 | 0,476 | 0,430 | 0,53 | 0,000 |
| seq79 | simvastatin_transatlantic_Cohort | 1644 | 8314 | 579 | 0,502 | 0,451 | 0,56 | 0,000 |
| stomach | simvastatin_transatlantic_Cohort | 261 | 1182 | 119 | 0,743 | 0,576 | 0,96 | 0,022 |
| bladder | simvastatin_UVA_Cohort | 32 | 397 | 6 | 0,234 | 0,095 | 0,58 | 0,002 |
| brain | simvastatin_UVA_Cohort | 29 | 518 | 9 | 0,525 | 0,231 | 1,19 | 0,124 |
| breast | simvastatin_UVA_Cohort | 39 | 711 | 14 | 0,521 | 0,269 | 1,01 | 0,052 |
| cancer | simvastatin_UVA_Cohort | 1152 | 16761 | 351 | 0,431 | 0,379 | 0,49 | 0,000 |
| colorectal | simvastatin_UVA_Cohort | 39 | 677 | 10 | 0,269 | 0,132 | 0,55 | 0,000 |
| esophagus | simvastatin_UVA_Cohort | 16 | 193 | 6 | 0,587 | 0,208 | 1,66 | 0,320 |
| kidney | simvastatin_UVA_Cohort | 11 | 182 | 0 | 0,000 | 0,000 | nan | nan |
| liver | simvastatin_UVA_Cohort | 6 | 168 | 1 | 0,142 | 0,017 | 1,20 | 0,072 |
| lung | simvastatin_UVA_Cohort | 70 | 826 | 27 | 0,597 | 0,366 | 0,97 | 0,039 |
| ovaries | simvastatin_UVA_Cohort | 27 | 391 | 6 | 0,383 | 0,146 | 1,01 | 0,051 |
| pancreas | simvastatin_UVA_Cohort | 15 | 305 | 6 | 0,391 | 0,148 | 1,03 | 0,058 |
| prostate | simvastatin_UVA_Cohort | 60 | 782 | 18 | 0,377 | 0,217 | 0,66 | 0,001 |
| seq77 | simvastatin_UVA_Cohort | 82 | 1433 | 24 | 0,363 | 0,226 | 0,58 | 0,000 |
| seq78 | simvastatin_UVA_Cohort | 159 | 2503 | 40 | 0,331 | 0,230 | 0,48 | 0,000 |
| seq79 | simvastatin_UVA_Cohort | 147 | 2353 | 39 | 0,325 | 0,225 | 0,47 | 0,000 |
| stomach | simvastatin_UVA_Cohort | 9 | 123 | 3 | 0,584 | 0,133 | 2,59 | 0,487 |
| bladder | statins_charite_Cohort | 1115 | 2094 | 548 | 0,891 | 0,778 | 1,02 | 0,096 |
| brain | statins_charite_Cohort | 303 | 1302 | 127 | 0,583 | 0,460 | 0,74 | 0,000 |
| breast | statins_charite_Cohort | 372 | 1363 | 128 | 0,465 | 0,371 | 0,59 | 0,000 |
| cancer | statins_charite_Cohort | 14079 | 36497 | 5295 | 0,535 | 0,515 | 0,56 | 0,000 |
| cancersubentities | statins_charite_Cohort | 8604 | 21890 | 3344 | 0,560 | 0,533 | 0,59 | 0,000 |
| colorectal | statins_charite_Cohort | 1145 | 3133 | 386 | 0,442 | 0,387 | 0,51 | 0,000 |
| esophagus | statins_charite_Cohort | 279 | 699 | 115 | 0,578 | 0,445 | 0,75 | 0,000 |
| kidney | statins_charite_Cohort | 705 | 1513 | 318 | 0,816 | 0,688 | 0,97 | 0,019 |
| liver | statins_charite_Cohort | 557 | 1645 | 162 | 0,343 | 0,281 | 0,42 | 0,000 |
| lung | statins_charite_Cohort | 1164 | 2651 | 507 | 0,720 | 0,631 | 0,82 | 0,000 |
| ovaries | statins_charite_Cohort | 206 | 837 | 66 | 0,428 | 0,314 | 0,58 | 0,000 |
| pancreas | statins_charite_Cohort | 535 | 1476 | 191 | 0,490 | 0,403 | 0,60 | 0,000 |
| prostate | statins_charite_Cohort | 1253 | 2395 | 492 | 0,564 | 0,495 | 0,64 | 0,000 |
| seq77 | statins_charite_Cohort | 1791 | 5354 | 628 | 0,480 | 0,432 | 0,53 | 0,000 |
| seq78 | statins_charite_Cohort | 2252 | 7125 | 699 | 0,389 | 0,353 | 0,43 | 0,000 |
| seq79 | statins_charite_Cohort | 1985 | 5955 | 671 | 0,433 | 0,392 | 0,48 | 0,000 |
| stomach | statins_charite_Cohort | 341 | 1058 | 132 | 0,496 | 0,393 | 0,63 | 0,000 |
| bladder | statins_transatlantic_Cohort | 1231 | 2480 | 570 | 0,842 | 0,739 | 0,96 | 0,009 |
| brain | statins_transatlantic_Cohort | 434 | 1823 | 154 | 0,525 | 0,426 | 0,65 | 0,000 |
| breast | statins_transatlantic_Cohort | 506 | 2067 | 158 | 0,431 | 0,352 | 0,53 | 0,000 |
| cancer | statins_transatlantic_Cohort | 18016 | 53113 | 6425 | 0,496 | 0,480 | 0,51 | 0,000 |
| colorectal | statins_transatlantic_Cohort | 1285 | 3803 | 425 | 0,439 | 0,386 | 0,50 | 0,000 |
| esophagus | statins_transatlantic_Cohort | 333 | 886 | 140 | 0,630 | 0,496 | 0,80 | 0,000 |
| kidney | statins_transatlantic_Cohort | 734 | 1688 | 325 | 0,808 | 0,685 | 0,95 | 0,012 |
| liver | statins_transatlantic_Cohort | 634 | 1814 | 169 | 0,351 | 0,289 | 0,43 | 0,000 |
| lung | statins_transatlantic_Cohort | 1358 | 3449 | 596 | 0,688 | 0,611 | 0,77 | 0,000 |
| ovaries | statins_transatlantic_Cohort | 295 | 1225 | 82 | 0,365 | 0,279 | 0,48 | 0,000 |
| pancreas | statins_transatlantic_Cohort | 597 | 1774 | 213 | 0,490 | 0,408 | 0,59 | 0,000 |
| prostate | statins_transatlantic_Cohort | 1467 | 3151 | 572 | 0,564 | 0,501 | 0,64 | 0,000 |
| seq77 | statins_transatlantic_Cohort | 2040 | 6753 | 693 | 0,452 | 0,410 | 0,50 | 0,000 |
| seq78 | statins_transatlantic_Cohort | 2782 | 9574 | 827 | 0,370 | 0,339 | 0,40 | 0,000 |
| seq79 | statins_transatlantic_Cohort | 2553 | 8269 | 808 | 0,412 | 0,376 | 0,45 | 0,000 |
| stomach | statins_transatlantic_Cohort | 380 | 1176 | 136 | 0,497 | 0,396 | 0,63 | 0,000 |
| bladder | statins_UVA_Cohort | 96 | 386 | 22 | 0,215 | 0,131 | 0,35 | 0,000 |
| brain | statins_UVA_Cohort | 91 | 521 | 27 | 0,366 | 0,230 | 0,58 | 0,000 |
| breast | statins_UVA_Cohort | 138 | 704 | 30 | 0,298 | 0,194 | 0,46 | 0,000 |

| | | | | | | | | |
|---|---|---|---|---|---|---|---|---|
| cancer | statins_UVA_Cohort | 4086 | 16616 | 1130 | 0,338 | 0,314 | 0,36 | 0,000 |
| cancersubentities | statins_UVA_Cohort | 1901 | 7514 | 500 | 0,319 | 0,286 | 0,36 | 0,000 |
| colorectal | statins_UVA_Cohort | 172 | 670 | 39 | 0,277 | 0,190 | 0,40 | 0,000 |
| esophagus | statins_UVA_Cohort | 52 | 187 | 25 | 0,608 | 0,349 | 1,06 | 0,078 |
| kidney | statins_UVA_Cohort | 43 | 175 | 7 | 0,196 | 0,083 | 0,46 | 0,000 |
| liver | statins_UVA_Cohort | 36 | 169 | 7 | 0,183 | 0,078 | 0,43 | 0,000 |
| lung | statins_UVA_Cohort | 244 | 798 | 89 | 0,506 | 0,382 | 0,67 | 0,000 |
| ovaries | statins_UVA_Cohort | 87 | 388 | 16 | 0,269 | 0,151 | 0,48 | 0,000 |
| pancreas | statins_UVA_Cohort | 86 | 298 | 22 | 0,330 | 0,196 | 0,56 | 0,000 |
| prostate | statins_UVA_Cohort | 245 | 756 | 80 | 0,411 | 0,308 | 0,55 | 0,000 |
| seq77 | statins_UVA_Cohort | 312 | 1399 | 65 | 0,248 | 0,186 | 0,33 | 0,000 |
| seq78 | statins_UVA_Cohort | 562 | 2449 | 128 | 0,263 | 0,214 | 0,32 | 0,000 |
| seq79 | statins_UVA_Cohort | 534 | 2314 | 137 | 0,295 | 0,241 | 0,36 | 0,000 |
| stomach | statins_UVA_Cohort | 26 | 118 | 4 | 0,167 | 0,055 | 0,51 | 0,002 |

| | | 1:1 matched Cohort MEDIAN Odds-Ratio | | | |
|---|---|---|---|---|---|
| Diagnose | Drug | Odds Ratio | Conf-Low | Conf-Up | P-Value |
| bladder | atorvastatin_charite_Cohort | 0,569 | 0,424 | 0,76 | 0,000 |
| brain | atorvastatin_charite_Cohort | 0,426 | 0,251 | 0,72 | 0,002 |
| breast | atorvastatin_charite_Cohort | 0,367 | 0,216 | 0,63 | 0,000 |
| cancer | atorvastatin_charite_Cohort | 0,340 | 0,311 | 0,37 | 0,000 |
| colorectal | atorvastatin_charite_Cohort | 0,286 | 0,208 | 0,40 | 0,000 |
| esophagus | atorvastatin_charite_Cohort | 0,176 | 0,078 | 0,40 | 0,000 |
| kidney | atorvastatin_charite_Cohort | 0,503 | 0,348 | 0,73 | 0,000 |
| liver | atorvastatin_charite_Cohort | 0,177 | 0,105 | 0,30 | 0,000 |
| lung | atorvastatin_charite_Cohort | 0,444 | 0,332 | 0,59 | 0,000 |
| ovaries | atorvastatin_charite_Cohort | 0,198 | 0,082 | 0,48 | 0,000 |
| pancreas | atorvastatin_charite_Cohort | 0,366 | 0,236 | 0,57 | 0,000 |
| prostate | atorvastatin_charite_Cohort | 0,320 | 0,236 | 0,43 | 0,000 |
| seq77 | atorvastatin_charite_Cohort | 0,262 | 0,201 | 0,34 | 0,000 |
| seq78 | atorvastatin_charite_Cohort | 0,200 | 0,155 | 0,26 | 0,000 |
| seq79 | atorvastatin_charite_Cohort | 0,251 | 0,196 | 0,32 | 0,000 |
| stomach | atorvastatin_charite_Cohort | 0,213 | 0,113 | 0,40 | 0,000 |
| bladder | atorvastatin_transatlantic_Cohort | 0,397 | 0,307 | 0,51 | 0,000 |
| brain | atorvastatin_transatlantic_Cohort | 0,335 | 0,222 | 0,51 | 0,000 |
| breast | atorvastatin_transatlantic_Cohort | 0,255 | 0,168 | 0,39 | 0,000 |
| cancer | atorvastatin_transatlantic_Cohort | 0,300 | 0,281 | 0,32 | 0,000 |
| colorectal | atorvastatin_transatlantic_Cohort | 0,236 | 0,181 | 0,31 | 0,000 |
| esophagus | atorvastatin_transatlantic_Cohort | 0,273 | 0,165 | 0,45 | 0,000 |
| kidney | atorvastatin_transatlantic_Cohort | 0,370 | 0,266 | 0,52 | 0,000 |
| liver | atorvastatin_transatlantic_Cohort | 0,153 | 0,098 | 0,24 | 0,000 |
| lung | atorvastatin_transatlantic_Cohort | 0,409 | 0,326 | 0,51 | 0,000 |
| ovaries | atorvastatin_transatlantic_Cohort | 0,164 | 0,086 | 0,31 | 0,000 |
| pancreas | atorvastatin_transatlantic_Cohort | 0,305 | 0,213 | 0,44 | 0,000 |
| prostate | atorvastatin_transatlantic_Cohort | 0,307 | 0,243 | 0,39 | 0,000 |
| seq77 | atorvastatin_transatlantic_Cohort | 0,219 | 0,177 | 0,27 | 0,000 |
| seq78 | atorvastatin_transatlantic_Cohort | 0,189 | 0,155 | 0,23 | 0,000 |
| seq79 | atorvastatin_transatlantic_Cohort | 0,245 | 0,204 | 0,30 | 0,000 |
| stomach | atorvastatin_transatlantic_Cohort | 0,153 | 0,085 | 0,28 | 0,000 |
| bladder | atorvastatin_UVA_Cohort | 0,249 | 0,133 | 0,47 | 0,000 |
| brain | atorvastatin_UVA_Cohort | 0,256 | 0,130 | 0,50 | 0,000 |
| breast | atorvastatin_UVA_Cohort | 0,195 | 0,098 | 0,39 | 0,000 |
| cancer | atorvastatin_UVA_Cohort | 0,282 | 0,255 | 0,31 | 0,000 |
| colorectal | atorvastatin_UVA_Cohort | 0,251 | 0,153 | 0,41 | 0,000 |
| esophagus | atorvastatin_UVA_Cohort | 0,561 | 0,274 | 1,15 | 0,114 |
| kidney | atorvastatin_UVA_Cohort | 0,266 | 0,096 | 0,73 | 0,011 |
| liver | atorvastatin_UVA_Cohort | 0,241 | 0,088 | 0,66 | 0,006 |
| lung | atorvastatin_UVA_Cohort | 0,413 | 0,282 | 0,61 | 0,000 |
| ovaries | atorvastatin_UVA_Cohort | 0,168 | 0,064 | 0,44 | 0,000 |
| pancreas | atorvastatin_UVA_Cohort | 0,327 | 0,166 | 0,64 | 0,001 |
| prostate | atorvastatin_UVA_Cohort | 0,342 | 0,233 | 0,50 | 0,000 |
| seq77 | atorvastatin_UVA_Cohort | 0,224 | 0,151 | 0,33 | 0,000 |
| seq78 | atorvastatin_UVA_Cohort | 0,210 | 0,157 | 0,28 | 0,000 |
| seq79 | atorvastatin_UVA_Cohort | 0,256 | 0,194 | 0,34 | 0,000 |
| stomach | atorvastatin_UVA_Cohort | 0,080 | 0,010 | 0,62 | 0,016 |
| bladder | fluvastatin_charite_Cohort | 0,749 | 0,353 | 1,59 | 0,460 |
| brain | fluvastatin_charite_Cohort | 0,000 | 0,000 | nan | nan |
| breast | fluvastatin_charite_Cohort | 0,308 | 0,063 | 1,51 | 0,147 |
| cancer | fluvastatin_charite_Cohort | 0,480 | 0,385 | 0,60 | 0,000 |
| colorectal | fluvastatin_charite_Cohort | 0,398 | 0,175 | 0,91 | 0,028 |
| esophagus | fluvastatin_charite_Cohort | 1,000 | 0,288 | 3,47 | 1,000 |

Supplementary Table 3
1:1 matched Cohort
Median Odds-Ratio

| | | | | | |
|---|---|---|---|---|---|
| kidney | fluvastatin_charite_Cohort | 1,339 | 0,599 | 2,99 | 0,487 |
| liver | fluvastatin_charite_Cohort | 0,319 | 0,104 | 0,99 | 0,046 |
| lung | fluvastatin_charite_Cohort | 0,419 | 0,183 | 0,96 | 0,039 |
| ovaries | fluvastatin_charite_Cohort | 0,249 | 0,028 | 2,23 | 0,215 |
| pancreas | fluvastatin_charite_Cohort | 0,199 | 0,044 | 0,91 | 0,036 |
| prostate | fluvastatin_charite_Cohort | 0,569 | 0,279 | 1,16 | 0,121 |
| seq77 | fluvastatin_charite_Cohort | 0,269 | 0,134 | 0,54 | 0,000 |
| seq78 | fluvastatin_charite_Cohort | 0,366 | 0,213 | 0,63 | 0,000 |
| seq79 | fluvastatin_charite_Cohort | 0,454 | 0,260 | 0,79 | 0,006 |
| stomach | fluvastatin_charite_Cohort | 0,166 | 0,020 | 1,38 | 0,096 |
| bladder | fluvastatin_transatlantic_Cohort | 0,774 | 0,363 | 1,65 | 0,517 |
| brain | fluvastatin_transatlantic_Cohort | 0,000 | 0,000 | nan | nan |
| breast | fluvastatin_transatlantic_Cohort | 0,333 | 0,067 | 1,65 | 0,179 |
| cancer | fluvastatin_transatlantic_Cohort | 0,514 | 0,412 | 0,64 | 0,000 |
| colorectal | fluvastatin_transatlantic_Cohort | 0,499 | 0,224 | 1,11 | 0,089 |
| esophagus | fluvastatin_transatlantic_Cohort | 1,000 | 0,288 | 3,47 | 1,000 |
| kidney | fluvastatin_transatlantic_Cohort | 1,658 | 0,704 | 3,91 | 0,250 |
| liver | fluvastatin_transatlantic_Cohort | 0,443 | 0,136 | 1,44 | 0,177 |
| lung | fluvastatin_transatlantic_Cohort | 0,469 | 0,202 | 1,09 | 0,078 |
| ovaries | fluvastatin_transatlantic_Cohort | 0,333 | 0,035 | 3,20 | 0,345 |
| pancreas | fluvastatin_transatlantic_Cohort | 0,249 | 0,053 | 1,18 | 0,078 |
| prostate | fluvastatin_transatlantic_Cohort | 0,544 | 0,269 | 1,10 | 0,090 |
| seq77 | fluvastatin_transatlantic_Cohort | 0,312 | 0,153 | 0,64 | 0,001 |
| seq78 | fluvastatin_transatlantic_Cohort | 0,423 | 0,243 | 0,73 | 0,002 |
| seq79 | fluvastatin_transatlantic_Cohort | 0,455 | 0,260 | 0,79 | 0,006 |
| stomach | fluvastatin_transatlantic_Cohort | 0,166 | 0,020 | 1,38 | 0,096 |
| bladder | fluvastatin_UVA_Cohort | nan | nan | nan | nan |
| brain | fluvastatin_UVA_Cohort | 0,000 | 0,000 | nan | nan |
| breast | fluvastatin_UVA_Cohort | 0,000 | 0,000 | nan | nan |
| cancer | fluvastatin_UVA_Cohort | 0,333 | 0,035 | 3,21 | 0,345 |
| colorectal | fluvastatin_UVA_Cohort | inf | 0,062 | inf | nan |
| esophagus | fluvastatin_UVA_Cohort | nan | nan | nan | nan |
| kidney | fluvastatin_UVA_Cohort | 0,000 | 0,000 | nan | nan |
| liver | fluvastatin_UVA_Cohort | 0,000 | 0,000 | nan | nan |
| lung | fluvastatin_UVA_Cohort | 0,000 | 0,000 | nan | nan |
| ovaries | fluvastatin_UVA_Cohort | 0,000 | 0,000 | nan | nan |
| pancreas | fluvastatin_UVA_Cohort | 0,000 | 0,000 | nan | nan |
| prostate | fluvastatin_UVA_Cohort | 0,000 | 0,000 | nan | nan |
| seq77 | fluvastatin_UVA_Cohort | 0,000 | 0,000 | nan | nan |
| seq78 | fluvastatin_UVA_Cohort | 0,000 | 0,000 | nan | nan |
| seq79 | fluvastatin_UVA_Cohort | 0,000 | 0,000 | nan | nan |
| stomach | fluvastatin_UVA_Cohort | 0,000 | 0,000 | nan | nan |
| bladder | pravastatin_charite_Cohort | 1,000 | 0,519 | 1,93 | 1,000 |
| brain | pravastatin_charite_Cohort | 0,285 | 0,059 | 1,37 | 0,118 |
| breast | pravastatin_charite_Cohort | 0,713 | 0,226 | 2,25 | 0,576 |
| cancer | pravastatin_charite_Cohort | 0,659 | 0,545 | 0,80 | 0,000 |
| colorectal | pravastatin_charite_Cohort | 0,244 | 0,100 | 0,60 | 0,002 |
| esophagus | pravastatin_charite_Cohort | 0,462 | 0,117 | 1,83 | 0,274 |
| kidney | pravastatin_charite_Cohort | 0,881 | 0,390 | 1,99 | 0,773 |
| liver | pravastatin_charite_Cohort | 0,066 | 0,009 | 0,50 | 0,008 |
| lung | pravastatin_charite_Cohort | 0,569 | 0,280 | 1,16 | 0,120 |

| | | | | | |
|---|---|---|---|---|---|
| ovaries | pravastatin_charite_Cohort | 1,101 | 0,343 | 3,53 | 0,881 |
| pancreas | pravastatin_charite_Cohort | 0,453 | 0,157 | 1,31 | 0,143 |
| prostate | pravastatin_charite_Cohort | 0,939 | 0,530 | 1,66 | 0,840 |
| seq77 | pravastatin_charite_Cohort | 0,570 | 0,344 | 0,94 | 0,028 |
| seq78 | pravastatin_charite_Cohort | 0,430 | 0,260 | 0,71 | 0,001 |
| seq79 | pravastatin_charite_Cohort | 0,407 | 0,235 | 0,71 | 0,001 |
| stomach | pravastatin_charite_Cohort | 0,534 | 0,158 | 1,81 | 0,317 |
| bladder | pravastatin_transatlantic_Cohort | 0,446 | 0,255 | 0,78 | 0,005 |
| brain | pravastatin_transatlantic_Cohort | 0,341 | 0,135 | 0,86 | 0,023 |
| breast | pravastatin_transatlantic_Cohort | 0,407 | 0,187 | 0,89 | 0,023 |
| cancer | pravastatin_transatlantic_Cohort | 0,475 | 0,416 | 0,54 | 0,000 |
| colorectal | pravastatin_transatlantic_Cohort | 0,228 | 0,122 | 0,43 | 0,000 |
| esophagus | pravastatin_transatlantic_Cohort | 0,318 | 0,103 | 0,98 | 0,046 |
| kidney | pravastatin_transatlantic_Cohort | 0,562 | 0,284 | 1,11 | 0,098 |
| liver | pravastatin_transatlantic_Cohort | 0,073 | 0,017 | 0,31 | 0,000 |
| lung | pravastatin_transatlantic_Cohort | 0,534 | 0,328 | 0,87 | 0,012 |
| ovaries | pravastatin_transatlantic_Cohort | 0,748 | 0,314 | 1,78 | 0,523 |
| pancreas | pravastatin_transatlantic_Cohort | 0,330 | 0,148 | 0,74 | 0,007 |
| prostate | pravastatin_transatlantic_Cohort | 0,818 | 0,539 | 1,24 | 0,350 |
| seq77 | pravastatin_transatlantic_Cohort | 0,325 | 0,210 | 0,50 | 0,000 |
| seq78 | pravastatin_transatlantic_Cohort | 0,321 | 0,225 | 0,46 | 0,000 |
| seq79 | pravastatin_transatlantic_Cohort | 0,311 | 0,212 | 0,46 | 0,000 |
| stomach | pravastatin_transatlantic_Cohort | 0,283 | 0,093 | 0,86 | 0,026 |
| bladder | pravastatin_UVA_Cohort | 0,000 | 0,000 | nan | nan |
| brain | pravastatin_UVA_Cohort | 0,395 | 0,123 | 1,27 | 0,119 |
| breast | pravastatin_UVA_Cohort | 0,330 | 0,106 | 1,03 | 0,055 |
| cancer | pravastatin_UVA_Cohort | 0,386 | 0,320 | 0,47 | 0,000 |
| colorectal | pravastatin_UVA_Cohort | 0,382 | 0,148 | 0,99 | 0,046 |
| esophagus | pravastatin_UVA_Cohort | 0,196 | 0,023 | 1,69 | 0,137 |
| kidney | pravastatin_UVA_Cohort | 0,444 | 0,082 | 2,39 | 0,351 |
| liver | pravastatin_UVA_Cohort | 0,246 | 0,027 | 2,22 | 0,214 |
| lung | pravastatin_UVA_Cohort | 0,644 | 0,318 | 1,30 | 0,224 |
| ovaries | pravastatin_UVA_Cohort | 0,349 | 0,092 | 1,31 | 0,121 |
| pancreas | pravastatin_UVA_Cohort | 0,369 | 0,097 | 1,40 | 0,144 |
| prostate | pravastatin_UVA_Cohort | 0,795 | 0,424 | 1,49 | 0,484 |
| seq77 | pravastatin_UVA_Cohort | 0,089 | 0,027 | 0,29 | 0,000 |
| seq78 | pravastatin_UVA_Cohort | 0,320 | 0,188 | 0,55 | 0,000 |
| seq79 | pravastatin_UVA_Cohort | 0,290 | 0,169 | 0,50 | 0,000 |
| stomach | pravastatin_UVA_Cohort | 0,000 | 0,000 | nan | nan |
| bladder | simvastatin_charite_Cohort | 0,926 | 0,798 | 1,07 | 0,315 |
| brain | simvastatin_charite_Cohort | 0,672 | 0,516 | 0,87 | 0,003 |
| breast | simvastatin_charite_Cohort | 0,512 | 0,397 | 0,66 | 0,000 |
| cancer | simvastatin_charite_Cohort | 0,612 | 0,587 | 0,64 | 0,000 |
| colorectal | simvastatin_charite_Cohort | 0,506 | 0,436 | 0,59 | 0,000 |
| esophagus | simvastatin_charite_Cohort | 0,710 | 0,534 | 0,95 | 0,019 |
| kidney | simvastatin_charite_Cohort | 0,830 | 0,688 | 1,00 | 0,052 |
| liver | simvastatin_charite_Cohort | 0,406 | 0,329 | 0,50 | 0,000 |
| lung | simvastatin_charite_Cohort | 0,794 | 0,689 | 0,92 | 0,002 |
| ovaries | simvastatin_charite_Cohort | 0,452 | 0,320 | 0,64 | 0,000 |
| pancreas | simvastatin_charite_Cohort | 0,563 | 0,455 | 0,70 | 0,000 |
| prostate | simvastatin_charite_Cohort | 0,622 | 0,539 | 0,72 | 0,000 |
| seq77 | simvastatin_charite_Cohort | 0,538 | 0,479 | 0,61 | 0,000 |
| seq78 | simvastatin_charite_Cohort | 0,445 | 0,401 | 0,50 | 0,000 |
| seq79 | simvastatin_charite_Cohort | 0,482 | 0,431 | 0,54 | 0,000 |

| | | | | | |
|---|---|---|---|---|---|
| stomach | simvastatin_charite_Cohort | 0,603 | 0,469 | 0,77 | 0,000 |
| bladder | simvastatin_transatlantic_Cohort | 1,055 | 0,911 | 1,22 | 0,486 |
| brain | simvastatin_transatlantic_Cohort | 0,660 | 0,514 | 0,85 | 0,001 |
| breast | simvastatin_transatlantic_Cohort | 0,538 | 0,425 | 0,68 | 0,000 |
| cancer | simvastatin_transatlantic_Cohort | 0,633 | 0,608 | 0,66 | 0,000 |
| colorectal | simvastatin_transatlantic_Cohort | 0,597 | 0,516 | 0,69 | 0,000 |
| esophagus | simvastatin_transatlantic_Cohort | 0,788 | 0,597 | 1,04 | 0,092 |
| kidney | simvastatin_transatlantic_Cohort | 1,030 | 0,849 | 1,25 | 0,777 |
| liver | simvastatin_transatlantic_Cohort | 0,508 | 0,409 | 0,63 | 0,000 |
| lung | simvastatin_transatlantic_Cohort | 0,891 | 0,776 | 1,02 | 0,101 |
| ovaries | simvastatin_transatlantic_Cohort | 0,457 | 0,332 | 0,63 | 0,000 |
| pancreas | simvastatin_transatlantic_Cohort | 0,651 | 0,526 | 0,80 | 0,000 |
| prostate | simvastatin_transatlantic_Cohort | 0,661 | 0,576 | 0,76 | 0,000 |
| seq77 | simvastatin_transatlantic_Cohort | 0,604 | 0,539 | 0,68 | 0,000 |
| seq78 | simvastatin_transatlantic_Cohort | 0,476 | 0,431 | 0,53 | 0,000 |
| seq79 | simvastatin_transatlantic_Cohort | 0,505 | 0,454 | 0,56 | 0,000 |
| stomach | simvastatin_transatlantic_Cohort | 0,718 | 0,557 | 0,92 | 0,010 |
| bladder | simvastatin_UVA_Cohort | 0,219 | 0,089 | 0,54 | 0,001 |
| brain | simvastatin_UVA_Cohort | 0,555 | 0,243 | 1,27 | 0,163 |
| breast | simvastatin_UVA_Cohort | 0,509 | 0,265 | 0,98 | 0,042 |
| cancer | simvastatin_UVA_Cohort | 0,428 | 0,377 | 0,49 | 0,000 |
| colorectal | simvastatin_UVA_Cohort | 0,267 | 0,131 | 0,54 | 0,000 |
| esophagus | simvastatin_UVA_Cohort | 0,559 | 0,201 | 1,56 | 0,269 |
| kidney | simvastatin_UVA_Cohort | 0,000 | 0,000 | nan | nan |
| liver | simvastatin_UVA_Cohort | 0,129 | 0,016 | 1,05 | 0,055 |
| lung | simvastatin_UVA_Cohort | 0,601 | 0,368 | 0,98 | 0,041 |
| ovaries | simvastatin_UVA_Cohort | 0,354 | 0,138 | 0,91 | 0,031 |
| pancreas | simvastatin_UVA_Cohort | 0,375 | 0,144 | 0,98 | 0,044 |
| prostate | simvastatin_UVA_Cohort | 0,364 | 0,210 | 0,63 | 0,000 |
| seq77 | simvastatin_UVA_Cohort | 0,364 | 0,227 | 0,59 | 0,000 |
| seq78 | simvastatin_UVA_Cohort | 0,328 | 0,228 | 0,47 | 0,000 |
| seq79 | simvastatin_UVA_Cohort | 0,333 | 0,230 | 0,48 | 0,000 |
| stomach | simvastatin_UVA_Cohort | 0,487 | 0,119 | 2,00 | 0,322 |
| bladder | statins_charite_Cohort | 0,893 | 0,779 | 1,02 | 0,103 |
| brain | statins_charite_Cohort | 0,580 | 0,458 | 0,73 | 0,000 |
| breast | statins_charite_Cohort | 0,468 | 0,373 | 0,59 | 0,000 |
| cancer | statins_charite_Cohort | 0,535 | 0,515 | 0,56 | 0,000 |
| cancersubentities | statins_charite_Cohort | 0,558 | 0,532 | 0,59 | 0,000 |
| colorectal | statins_charite_Cohort | 0,442 | 0,386 | 0,50 | 0,000 |
| esophagus | statins_charite_Cohort | 0,576 | 0,443 | 0,75 | 0,000 |
| kidney | statins_charite_Cohort | 0,816 | 0,689 | 0,97 | 0,019 |
| liver | statins_charite_Cohort | 0,342 | 0,280 | 0,42 | 0,000 |
| lung | statins_charite_Cohort | 0,712 | 0,625 | 0,81 | 0,000 |
| ovaries | statins_charite_Cohort | 0,423 | 0,310 | 0,58 | 0,000 |
| pancreas | statins_charite_Cohort | 0,494 | 0,406 | 0,60 | 0,000 |
| prostate | statins_charite_Cohort | 0,565 | 0,495 | 0,64 | 0,000 |
| seq77 | statins_charite_Cohort | 0,480 | 0,432 | 0,53 | 0,000 |
| seq78 | statins_charite_Cohort | 0,389 | 0,353 | 0,43 | 0,000 |
| seq79 | statins_charite_Cohort | 0,430 | 0,389 | 0,48 | 0,000 |
| stomach | statins_charite_Cohort | 0,491 | 0,389 | 0,62 | 0,000 |
| bladder | statins_transatlantic_Cohort | 0,831 | 0,730 | 0,95 | 0,005 |
| brain | statins_transatlantic_Cohort | 0,527 | 0,428 | 0,65 | 0,000 |
| breast | statins_transatlantic_Cohort | 0,426 | 0,349 | 0,52 | 0,000 |
| cancer | statins_transatlantic_Cohort | 0,494 | 0,478 | 0,51 | 1.97626258336E-323 |

Supplementary Table 3
1:1 matched Cohort
Median Odds-Ratio

| | | | | | |
|---|---|---|---|---|---|
| colorectal | statins_transatlantic_Cohort | 0,434 | 0,382 | 0,49 | 0,000 |
| esophagus | statins_transatlantic_Cohort | 0,637 | 0,502 | 0,81 | 0,000 |
| kidney | statins_transatlantic_Cohort | 0,803 | 0,681 | 0,95 | 0,009 |
| liver | statins_transatlantic_Cohort | 0,350 | 0,289 | 0,43 | 0,000 |
| lung | statins_transatlantic_Cohort | 0,682 | 0,606 | 0,77 | 0,000 |
| ovaries | statins_transatlantic_Cohort | 0,363 | 0,278 | 0,48 | 0,000 |
| pancreas | statins_transatlantic_Cohort | 0,486 | 0,405 | 0,58 | 0,000 |
| prostate | statins_transatlantic_Cohort | 0,558 | 0,495 | 0,63 | 0,000 |
| seq77 | statins_transatlantic_Cohort | 0,450 | 0,407 | 0,50 | 0,000 |
| seq78 | statins_transatlantic_Cohort | 0,369 | 0,339 | 0,40 | 0,000 |
| seq79 | statins_transatlantic_Cohort | 0,411 | 0,376 | 0,45 | 0,000 |
| stomach | statins_transatlantic_Cohort | 0,497 | 0,396 | 0,62 | 0,000 |
| bladder | statins_UVA_Cohort | 0,214 | 0,131 | 0,35 | 0,000 |
| brain | statins_UVA_Cohort | 0,352 | 0,222 | 0,56 | 0,000 |
| breast | statins_UVA_Cohort | 0,300 | 0,196 | 0,46 | 0,000 |
| cancer | statins_UVA_Cohort | 0,338 | 0,315 | 0,36 | 0,000 |
| cancersubentities | statins_UVA_Cohort | 0,317 | 0,284 | 0,35 | 0,000 |
| colorectal | statins_UVA_Cohort | 0,274 | 0,188 | 0,40 | 0,000 |
| esophagus | statins_UVA_Cohort | 0,605 | 0,349 | 1,05 | 0,073 |
| kidney | statins_UVA_Cohort | 0,186 | 0,080 | 0,44 | 0,000 |
| liver | statins_UVA_Cohort | 0,168 | 0,072 | 0,39 | 0,000 |
| lung | statins_UVA_Cohort | 0,501 | 0,378 | 0,66 | 0,000 |
| ovaries | statins_UVA_Cohort | 0,260 | 0,146 | 0,46 | 0,000 |
| pancreas | statins_UVA_Cohort | 0,323 | 0,192 | 0,54 | 0,000 |
| prostate | statins_UVA_Cohort | 0,422 | 0,316 | 0,56 | 0,000 |
| seq77 | statins_UVA_Cohort | 0,244 | 0,183 | 0,33 | 0,000 |
| seq78 | statins_UVA_Cohort | 0,263 | 0,213 | 0,32 | 0,000 |
| seq79 | statins_UVA_Cohort | 0,298 | 0,243 | 0,37 | 0,000 |
| stomach | statins_UVA_Cohort | 0,149 | 0,050 | 0,45 | 0,001 |

Supplementary Table 3
1:1 matched Cohort
Median Odds-Ratio

| | | 1:1 matched Cohort MEAN Relative Risk | | | |
|---|---|---|---|---|---|
| Diagnose | Drug | Relative Risk | Conf-Low | Conf-Up | P-Value |
| bladder | atorvastatin_charite_Cohort | 0,730 | 0,609 | 0,88 | 0,001 |
| brain | atorvastatin_charite_Cohort | 0,596 | 0,413 | 0,86 | 0,006 |
| breast | atorvastatin_charite_Cohort | 0,545 | 0,371 | 0,80 | 0,002 |
| cancer | atorvastatin_charite_Cohort | 0,513 | 0,481 | 0,55 | 0,000 |
| colorectal | atorvastatin_charite_Cohort | 0,450 | 0,352 | 0,57 | 0,000 |
| esophagus | atorvastatin_charite_Cohort | 0,294 | 0,148 | 0,58 | 0,000 |
| kidney | atorvastatin_charite_Cohort | 0,678 | 0,534 | 0,86 | 0,001 |
| liver | atorvastatin_charite_Cohort | 0,308 | 0,199 | 0,48 | 0,000 |
| lung | atorvastatin_charite_Cohort | 0,629 | 0,517 | 0,77 | 0,000 |
| ovaries | atorvastatin_charite_Cohort | 0,338 | 0,163 | 0,70 | 0,004 |
| pancreas | atorvastatin_charite_Cohort | 0,544 | 0,396 | 0,75 | 0,000 |
| prostate | atorvastatin_charite_Cohort | 0,499 | 0,400 | 0,62 | 0,000 |
| seq77 | atorvastatin_charite_Cohort | 0,418 | 0,341 | 0,51 | 0,000 |
| seq78 | atorvastatin_charite_Cohort | 0,342 | 0,278 | 0,42 | 0,000 |
| seq79 | atorvastatin_charite_Cohort | 0,413 | 0,340 | 0,50 | 0,000 |
| stomach | atorvastatin_charite_Cohort | 0,354 | 0,212 | 0,59 | 0,000 |
| bladder | atorvastatin_transatlantic_Cohort | 0,575 | 0,481 | 0,69 | 0,000 |
| brain | atorvastatin_transatlantic_Cohort | 0,502 | 0,370 | 0,68 | 0,000 |
| breast | atorvastatin_transatlantic_Cohort | 0,413 | 0,298 | 0,57 | 0,000 |
| cancer | atorvastatin_transatlantic_Cohort | 0,467 | 0,445 | 0,49 | 0,000 |
| colorectal | atorvastatin_transatlantic_Cohort | 0,388 | 0,315 | 0,48 | 0,000 |
| esophagus | atorvastatin_transatlantic_Cohort | 0,445 | 0,302 | 0,66 | 0,000 |
| kidney | atorvastatin_transatlantic_Cohort | 0,555 | 0,438 | 0,70 | 0,000 |
| liver | atorvastatin_transatlantic_Cohort | 0,267 | 0,181 | 0,40 | 0,000 |
| lung | atorvastatin_transatlantic_Cohort | 0,583 | 0,497 | 0,68 | 0,000 |
| ovaries | atorvastatin_transatlantic_Cohort | 0,295 | 0,171 | 0,51 | 0,000 |
| pancreas | atorvastatin_transatlantic_Cohort | 0,480 | 0,366 | 0,63 | 0,000 |
| prostate | atorvastatin_transatlantic_Cohort | 0,481 | 0,405 | 0,57 | 0,000 |
| seq77 | atorvastatin_transatlantic_Cohort | 0,365 | 0,307 | 0,43 | 0,000 |
| seq78 | atorvastatin_transatlantic_Cohort | 0,323 | 0,276 | 0,38 | 0,000 |
| seq79 | atorvastatin_transatlantic_Cohort | 0,399 | 0,345 | 0,46 | 0,000 |
| stomach | atorvastatin_transatlantic_Cohort | 0,271 | 0,163 | 0,45 | 0,000 |
| bladder | atorvastatin_UVA_Cohort | 0,410 | 0,252 | 0,67 | 0,000 |
| brain | atorvastatin_UVA_Cohort | 0,422 | 0,250 | 0,71 | 0,001 |
| breast | atorvastatin_UVA_Cohort | 0,322 | 0,182 | 0,57 | 0,000 |
| cancer | atorvastatin_UVA_Cohort | 0,448 | 0,416 | 0,48 | 0,000 |
| colorectal | atorvastatin_UVA_Cohort | 0,427 | 0,292 | 0,63 | 0,000 |
| esophagus | atorvastatin_UVA_Cohort | 0,728 | 0,471 | 1,13 | 0,155 |
| kidney | atorvastatin_UVA_Cohort | 0,432 | 0,200 | 0,94 | 0,033 |
| liver | atorvastatin_UVA_Cohort | 0,404 | 0,185 | 0,88 | 0,023 |
| lung | atorvastatin_UVA_Cohort | 0,587 | 0,453 | 0,76 | 0,000 |
| ovaries | atorvastatin_UVA_Cohort | 0,301 | 0,135 | 0,68 | 0,004 |
| pancreas | atorvastatin_UVA_Cohort | 0,520 | 0,319 | 0,85 | 0,009 |
| prostate | atorvastatin_UVA_Cohort | 0,519 | 0,396 | 0,68 | 0,000 |
| seq77 | atorvastatin_UVA_Cohort | 0,378 | 0,278 | 0,52 | 0,000 |
| seq78 | atorvastatin_UVA_Cohort | 0,357 | 0,282 | 0,45 | 0,000 |
| seq79 | atorvastatin_UVA_Cohort | 0,417 | 0,337 | 0,52 | 0,000 |
| stomach | atorvastatin_UVA_Cohort | 0,154 | 0,023 | 1,01 | 0,052 |
| bladder | fluvastatin_charite_Cohort | 0,877 | 0,576 | 1,34 | 0,552 |

Supplementary Table 3
1:1 matched Cohort
Mean Relative Risk

| | | | | | |
|---|---|---|---|---|---|
| brain | fluvastatin_charite_Cohort | 0,000 | 0,000 | nan | nan |
| breast | fluvastatin_charite_Cohort | 0,517 | 0,161 | 1,67 | 0,273 |
| cancer | fluvastatin_charite_Cohort | 0,651 | 0,562 | 0,76 | 0,000 |
| colorectal | fluvastatin_charite_Cohort | 0,549 | 0,305 | 0,99 | 0,045 |
| esophagus | fluvastatin_charite_Cohort | 1,013 | 0,557 | 1,85 | 0,970 |
| kidney | fluvastatin_charite_Cohort | 1,141 | 0,811 | 1,61 | 0,458 |
| liver | fluvastatin_charite_Cohort | 0,552 | 0,246 | 1,24 | 0,151 |
| lung | fluvastatin_charite_Cohort | 0,609 | 0,343 | 1,08 | 0,090 |
| ovaries | fluvastatin_charite_Cohort | 0,503 | 0,154 | 2,37 | 0,330 |
| pancreas | fluvastatin_charite_Cohort | 0,365 | 0,107 | 1,25 | 0,108 |
| prostate | fluvastatin_charite_Cohort | 0,741 | 0,475 | 1,16 | 0,188 |
| seq77 | fluvastatin_charite_Cohort | 0,436 | 0,253 | 0,75 | 0,003 |
| seq78 | fluvastatin_charite_Cohort | 0,538 | 0,363 | 0,80 | 0,002 |
| seq79 | fluvastatin_charite_Cohort | 0,616 | 0,419 | 0,90 | 0,013 |
| stomach | fluvastatin_charite_Cohort | 0,302 | 0,051 | 1,80 | 0,189 |
| bladder | fluvastatin_transatlantic_Cohort | 0,909 | 0,601 | 1,38 | 0,665 |
| brain | fluvastatin_transatlantic_Cohort | 0,000 | 0,000 | nan | nan |
| breast | fluvastatin_transatlantic_Cohort | 0,484 | 0,147 | 1,60 | 0,235 |
| cancer | fluvastatin_transatlantic_Cohort | 0,679 | 0,587 | 0,79 | 0,000 |
| colorectal | fluvastatin_transatlantic_Cohort | 0,655 | 0,384 | 1,12 | 0,120 |
| esophagus | fluvastatin_transatlantic_Cohort | 1,056 | 0,618 | 1,85 | 0,856 |
| kidney | fluvastatin_transatlantic_Cohort | 1,265 | 0,926 | 1,73 | 0,141 |
| liver | fluvastatin_transatlantic_Cohort | 0,609 | 0,270 | 1,37 | 0,234 |
| lung | fluvastatin_transatlantic_Cohort | 0,665 | 0,380 | 1,17 | 0,154 |
| ovaries | fluvastatin_transatlantic_Cohort | 0,532 | 0,160 | 2,52 | 0,376 |
| pancreas | fluvastatin_transatlantic_Cohort | 0,436 | 0,130 | 1,47 | 0,180 |
| prostate | fluvastatin_transatlantic_Cohort | 0,711 | 0,452 | 1,12 | 0,140 |
| seq77 | fluvastatin_transatlantic_Cohort | 0,484 | 0,283 | 0,83 | 0,008 |
| seq78 | fluvastatin_transatlantic_Cohort | 0,611 | 0,416 | 0,90 | 0,012 |
| seq79 | fluvastatin_transatlantic_Cohort | 0,639 | 0,437 | 0,94 | 0,021 |
| stomach | fluvastatin_transatlantic_Cohort | 0,329 | 0,057 | 1,92 | 0,217 |
| bladder | fluvastatin_UVA_Cohort | nan | nan | nan | nan |
| brain | fluvastatin_UVA_Cohort | 0,000 | 0,000 | nan | nan |
| breast | fluvastatin_UVA_Cohort | 0,000 | 0,000 | nan | nan |
| cancer | fluvastatin_UVA_Cohort | 0,605 | 0,126 | 2,98 | 0,544 |
| colorectal | fluvastatin_UVA_Cohort | 1,847 | 1,644 | 2,40 | 0,000 |
| esophagus | fluvastatin_UVA_Cohort | nan | nan | nan | nan |
| kidney | fluvastatin_UVA_Cohort | 0,000 | 0,000 | nan | nan |
| liver | fluvastatin_UVA_Cohort | 0,000 | 0,000 | nan | nan |
| lung | fluvastatin_UVA_Cohort | 0,000 | 0,000 | nan | nan |
| ovaries | fluvastatin_UVA_Cohort | 0,000 | 0,000 | nan | nan |
| pancreas | fluvastatin_UVA_Cohort | 0,000 | 0,000 | nan | nan |
| prostate | fluvastatin_UVA_Cohort | 0,000 | 0,000 | nan | nan |
| seq77 | fluvastatin_UVA_Cohort | 0,000 | 0,000 | nan | nan |
| seq78 | fluvastatin_UVA_Cohort | 0,000 | 0,000 | nan | nan |
| seq79 | fluvastatin_UVA_Cohort | 0,000 | 0,000 | nan | nan |
| stomach | fluvastatin_UVA_Cohort | 0,000 | 0,000 | nan | nan |
| bladder | pravastatin_charite_Cohort | 0,989 | 0,713 | 1,37 | 0,952 |
| brain | pravastatin_charite_Cohort | 0,486 | 0,149 | 1,59 | 0,235 |
| breast | pravastatin_charite_Cohort | 0,798 | 0,409 | 1,56 | 0,519 |
| cancer | pravastatin_charite_Cohort | 0,796 | 0,710 | 0,89 | 0,000 |
| colorectal | pravastatin_charite_Cohort | 0,411 | 0,203 | 0,84 | 0,014 |
| esophagus | pravastatin_charite_Cohort | 0,724 | 0,304 | 1,74 | 0,478 |

| | | | | | |
|---|---|---|---|---|---|
| kidney | pravastatin_charite_Cohort | 0,993 | 0,657 | 1,50 | 0,976 |
| liver | pravastatin_charite_Cohort | 0,129 | 0,019 | 0,85 | 0,034 |
| lung | pravastatin_charite_Cohort | 0,724 | 0,462 | 1,14 | 0,160 |
| ovaries | pravastatin_charite_Cohort | 1,083 | 0,637 | 1,85 | 0,782 |
| pancreas | pravastatin_charite_Cohort | 0,634 | 0,310 | 1,30 | 0,214 |
| prostate | pravastatin_charite_Cohort | 0,959 | 0,714 | 1,29 | 0,793 |
| seq77 | pravastatin_charite_Cohort | 0,743 | 0,541 | 1,02 | 0,066 |
| seq78 | pravastatin_charite_Cohort | 0,602 | 0,424 | 0,85 | 0,004 |
| seq79 | pravastatin_charite_Cohort | 0,587 | 0,398 | 0,86 | 0,007 |
| stomach | pravastatin_charite_Cohort | 0,714 | 0,331 | 1,55 | 0,399 |
| bladder | pravastatin_transatlantic_Cohort | 0,628 | 0,428 | 0,92 | 0,017 |
| brain | pravastatin_transatlantic_Cohort | 0,523 | 0,265 | 1,03 | 0,062 |
| breast | pravastatin_transatlantic_Cohort | 0,601 | 0,350 | 1,04 | 0,065 |
| cancer | pravastatin_transatlantic_Cohort | 0,645 | 0,590 | 0,70 | 0,000 |
| colorectal | pravastatin_transatlantic_Cohort | 0,375 | 0,226 | 0,62 | 0,000 |
| esophagus | pravastatin_transatlantic_Cohort | 0,537 | 0,235 | 1,23 | 0,141 |
| kidney | pravastatin_transatlantic_Cohort | 0,730 | 0,474 | 1,12 | 0,154 |
| liver | pravastatin_transatlantic_Cohort | 0,149 | 0,039 | 0,56 | 0,005 |
| lung | pravastatin_transatlantic_Cohort | 0,702 | 0,512 | 0,96 | 0,028 |
| ovaries | pravastatin_transatlantic_Cohort | 0,869 | 0,534 | 1,42 | 0,584 |
| pancreas | pravastatin_transatlantic_Cohort | 0,494 | 0,272 | 0,90 | 0,021 |
| prostate | pravastatin_transatlantic_Cohort | 0,903 | 0,720 | 1,13 | 0,385 |
| seq77 | pravastatin_transatlantic_Cohort | 0,486 | 0,350 | 0,68 | 0,000 |
| seq78 | pravastatin_transatlantic_Cohort | 0,491 | 0,375 | 0,64 | 0,000 |
| seq79 | pravastatin_transatlantic_Cohort | 0,480 | 0,360 | 0,64 | 0,000 |
| stomach | pravastatin_transatlantic_Cohort | 0,450 | 0,191 | 1,06 | 0,067 |
| bladder | pravastatin_UVA_Cohort | 0,000 | 0,000 | nan | nan |
| brain | pravastatin_UVA_Cohort | 0,591 | 0,263 | 1,33 | 0,206 |
| breast | pravastatin_UVA_Cohort | 0,519 | 0,225 | 1,20 | 0,124 |
| cancer | pravastatin_UVA_Cohort | 0,563 | 0,492 | 0,64 | 0,000 |
| colorectal | pravastatin_UVA_Cohort | 0,579 | 0,296 | 1,13 | 0,110 |
| esophagus | pravastatin_UVA_Cohort | 0,369 | 0,066 | 2,08 | 0,261 |
| kidney | pravastatin_UVA_Cohort | 0,652 | 0,216 | 1,99 | 0,459 |
| liver | pravastatin_UVA_Cohort | 0,424 | 0,077 | 2,36 | 0,330 |
| lung | pravastatin_UVA_Cohort | 0,780 | 0,511 | 1,19 | 0,253 |
| ovaries | pravastatin_UVA_Cohort | 0,564 | 0,222 | 1,44 | 0,233 |
| pancreas | pravastatin_UVA_Cohort | 0,569 | 0,221 | 1,47 | 0,245 |
| prostate | pravastatin_UVA_Cohort | 0,915 | 0,650 | 1,29 | 0,623 |
| seq77 | pravastatin_UVA_Cohort | 0,162 | 0,054 | 0,48 | 0,001 |
| seq78 | pravastatin_UVA_Cohort | 0,486 | 0,326 | 0,73 | 0,000 |
| seq79 | pravastatin_UVA_Cohort | 0,472 | 0,312 | 0,72 | 0,000 |
| stomach | pravastatin_UVA_Cohort | 0,000 | 0,000 | nan | nan |
| bladder | simvastatin_charite_Cohort | 0,963 | 0,893 | 1,04 | 0,334 |
| brain | simvastatin_charite_Cohort | 0,800 | 0,687 | 0,93 | 0,004 |
| breast | simvastatin_charite_Cohort | 0,687 | 0,585 | 0,81 | 0,000 |
| cancer | simvastatin_charite_Cohort | 0,764 | 0,746 | 0,78 | 0,000 |
| colorectal | simvastatin_charite_Cohort | 0,685 | 0,625 | 0,75 | 0,000 |
| esophagus | simvastatin_charite_Cohort | 0,849 | 0,724 | 1,00 | 0,043 |
| kidney | simvastatin_charite_Cohort | 0,906 | 0,820 | 1,00 | 0,053 |
| liver | simvastatin_charite_Cohort | 0,595 | 0,516 | 0,69 | 0,000 |
| lung | simvastatin_charite_Cohort | 0,895 | 0,829 | 0,97 | 0,005 |
| ovaries | simvastatin_charite_Cohort | 0,638 | 0,509 | 0,80 | 0,000 |
| pancreas | simvastatin_charite_Cohort | 0,728 | 0,639 | 0,83 | 0,000 |

| | | | | | |
|---|---|---|---|---|---|
| prostate | simvastatin_charite_Cohort | 0,779 | 0,717 | 0,85 | 0,000 |
| seq77 | simvastatin_charite_Cohort | 0,710 | 0,661 | 0,76 | 0,000 |
| seq78 | simvastatin_charite_Cohort | 0,627 | 0,585 | 0,67 | 0,000 |
| seq79 | simvastatin_charite_Cohort | 0,665 | 0,619 | 0,71 | 0,000 |
| stomach | simvastatin_charite_Cohort | 0,763 | 0,658 | 0,89 | 0,000 |
| bladder | simvastatin_transatlantic_Cohort | 1,030 | 0,958 | 1,11 | 0,431 |
| brain | simvastatin_transatlantic_Cohort | 0,800 | 0,691 | 0,93 | 0,003 |
| breast | simvastatin_transatlantic_Cohort | 0,706 | 0,608 | 0,82 | 0,000 |
| cancer | simvastatin_transatlantic_Cohort | 0,781 | 0,763 | 0,80 | 0,000 |
| colorectal | simvastatin_transatlantic_Cohort | 0,753 | 0,690 | 0,82 | 0,000 |
| esophagus | simvastatin_transatlantic_Cohort | 0,878 | 0,755 | 1,02 | 0,092 |
| kidney | simvastatin_transatlantic_Cohort | 1,009 | 0,917 | 1,11 | 0,864 |
| liver | simvastatin_transatlantic_Cohort | 0,676 | 0,589 | 0,78 | 0,000 |
| lung | simvastatin_transatlantic_Cohort | 0,945 | 0,879 | 1,02 | 0,123 |
| ovaries | simvastatin_transatlantic_Cohort | 0,637 | 0,515 | 0,79 | 0,000 |
| pancreas | simvastatin_transatlantic_Cohort | 0,788 | 0,696 | 0,89 | 0,000 |
| prostate | simvastatin_transatlantic_Cohort | 0,802 | 0,741 | 0,87 | 0,000 |
| seq77 | simvastatin_transatlantic_Cohort | 0,755 | 0,705 | 0,81 | 0,000 |
| seq78 | simvastatin_transatlantic_Cohort | 0,653 | 0,612 | 0,70 | 0,000 |
| seq79 | simvastatin_transatlantic_Cohort | 0,676 | 0,631 | 0,72 | 0,000 |
| stomach | simvastatin_transatlantic_Cohort | 0,853 | 0,740 | 0,98 | 0,027 |
| bladder | simvastatin_UVA_Cohort | 0,383 | 0,186 | 0,78 | 0,009 |
| brain | simvastatin_UVA_Cohort | 0,686 | 0,403 | 1,17 | 0,165 |
| breast | simvastatin_UVA_Cohort | 0,684 | 0,447 | 1,05 | 0,080 |
| cancer | simvastatin_UVA_Cohort | 0,606 | 0,555 | 0,66 | 0,000 |
| colorectal | simvastatin_UVA_Cohort | 0,427 | 0,247 | 0,74 | 0,002 |
| esophagus | simvastatin_UVA_Cohort | 0,734 | 0,388 | 1,39 | 0,348 |
| kidney | simvastatin_UVA_Cohort | 0,000 | 0,000 | nan | nan |
| liver | simvastatin_UVA_Cohort | 0,248 | 0,040 | 1,53 | 0,134 |
| lung | simvastatin_UVA_Cohort | 0,748 | 0,553 | 1,01 | 0,059 |
| ovaries | simvastatin_UVA_Cohort | 0,549 | 0,279 | 1,08 | 0,083 |
| pancreas | simvastatin_UVA_Cohort | 0,558 | 0,284 | 1,10 | 0,091 |
| prostate | simvastatin_UVA_Cohort | 0,550 | 0,371 | 0,82 | 0,003 |
| seq77 | simvastatin_UVA_Cohort | 0,535 | 0,379 | 0,75 | 0,000 |
| seq78 | simvastatin_UVA_Cohort | 0,501 | 0,383 | 0,66 | 0,000 |
| seq79 | simvastatin_UVA_Cohort | 0,494 | 0,376 | 0,65 | 0,000 |
| stomach | simvastatin_UVA_Cohort | 0,716 | 0,293 | 1,76 | 0,474 |
| bladder | statins_charite_Cohort | 0,943 | 0,879 | 1,01 | 0,100 |
| brain | statins_charite_Cohort | 0,743 | 0,644 | 0,86 | 0,000 |
| breast | statins_charite_Cohort | 0,647 | 0,559 | 0,75 | 0,000 |
| cancer | statins_charite_Cohort | 0,710 | 0,694 | 0,73 | 0,000 |
| cancersubentities | statins_charite_Cohort | 0,729 | 0,709 | 0,75 | 0,000 |
| colorectal | statins_charite_Cohort | 0,631 | 0,579 | 0,69 | 0,000 |
| esophagus | statins_charite_Cohort | 0,743 | 0,638 | 0,87 | 0,000 |
| kidney | statins_charite_Cohort | 0,900 | 0,823 | 0,99 | 0,021 |
| liver | statins_charite_Cohort | 0,533 | 0,466 | 0,61 | 0,000 |
| lung | statins_charite_Cohort | 0,842 | 0,783 | 0,90 | 0,000 |
| ovaries | statins_charite_Cohort | 0,611 | 0,498 | 0,75 | 0,000 |
| pancreas | statins_charite_Cohort | 0,672 | 0,596 | 0,76 | 0,000 |
| prostate | statins_charite_Cohort | 0,737 | 0,683 | 0,79 | 0,000 |
| seq77 | statins_charite_Cohort | 0,663 | 0,620 | 0,71 | 0,000 |
| seq78 | statins_charite_Cohort | 0,578 | 0,542 | 0,62 | 0,000 |
| seq79 | statins_charite_Cohort | 0,621 | 0,583 | 0,66 | 0,000 |

| | | | | | |
|---|---|---|---|---|---|
| stomach | statins_charite_Cohort | 0,676 | 0,586 | 0,78 | 0,000 |
| bladder | statins_transatlantic_Cohort | 0,915 | 0,855 | 0,98 | 0,010 |
| brain | statins_transatlantic_Cohort | 0,696 | 0,611 | 0,79 | 0,000 |
| breast | statins_transatlantic_Cohort | 0,613 | 0,538 | 0,70 | 0,000 |
| cancer | statins_transatlantic_Cohort | 0,676 | 0,662 | 0,69 | 0,000 |
| colorectal | statins_transatlantic_Cohort | 0,626 | 0,577 | 0,68 | 0,000 |
| esophagus | statins_transatlantic_Cohort | 0,780 | 0,681 | 0,90 | 0,000 |
| kidney | statins_transatlantic_Cohort | 0,895 | 0,820 | 0,98 | 0,014 |
| liver | statins_transatlantic_Cohort | 0,540 | 0,474 | 0,62 | 0,000 |
| lung | statins_transatlantic_Cohort | 0,820 | 0,768 | 0,88 | 0,000 |
| ovaries | statins_transatlantic_Cohort | 0,549 | 0,455 | 0,66 | 0,000 |
| pancreas | statins_transatlantic_Cohort | 0,671 | 0,599 | 0,75 | 0,000 |
| prostate | statins_transatlantic_Cohort | 0,735 | 0,686 | 0,79 | 0,000 |
| seq77 | statins_transatlantic_Cohort | 0,637 | 0,598 | 0,68 | 0,000 |
| seq78 | statins_transatlantic_Cohort | 0,557 | 0,525 | 0,59 | 0,000 |
| seq79 | statins_transatlantic_Cohort | 0,599 | 0,565 | 0,64 | 0,000 |
| stomach | statins_transatlantic_Cohort | 0,677 | 0,588 | 0,78 | 0,000 |
| bladder | statins_UVA_Cohort | 0,376 | 0,257 | 0,55 | 0,000 |
| brain | statins_UVA_Cohort | 0,545 | 0,394 | 0,75 | 0,000 |
| breast | statins_UVA_Cohort | 0,470 | 0,343 | 0,64 | 0,000 |
| cancer | statins_UVA_Cohort | 0,521 | 0,495 | 0,55 | 0,000 |
| cancersubentities | statins_UVA_Cohort | 0,501 | 0,464 | 0,54 | 0,000 |
| colorectal | statins_UVA_Cohort | 0,451 | 0,342 | 0,60 | 0,000 |
| esophagus | statins_UVA_Cohort | 0,760 | 0,551 | 1,05 | 0,094 |
| kidney | statins_UVA_Cohort | 0,343 | 0,175 | 0,68 | 0,002 |
| liver | statins_UVA_Cohort | 0,327 | 0,166 | 0,65 | 0,001 |
| lung | statins_UVA_Cohort | 0,682 | 0,574 | 0,81 | 0,000 |
| ovaries | statins_UVA_Cohort | 0,436 | 0,282 | 0,67 | 0,000 |
| pancreas | statins_UVA_Cohort | 0,512 | 0,356 | 0,74 | 0,000 |
| prostate | statins_UVA_Cohort | 0,599 | 0,497 | 0,72 | 0,000 |
| seq77 | statins_UVA_Cohort | 0,413 | 0,332 | 0,51 | 0,000 |
| seq78 | statins_UVA_Cohort | 0,433 | 0,371 | 0,51 | 0,000 |
| seq79 | statins_UVA_Cohort | 0,472 | 0,407 | 0,55 | 0,000 |
| stomach | statins_UVA_Cohort | 0,301 | 0,122 | 0,74 | 0,009 |

| | | 1:1 matched Cohort<br>MEDIAN Relative Risk | | | |
|---|---|---|---|---|---|
| Diagnose | Drug | Relative Risk | Conf-Low | Conf-Up | P-Value |
| bladder | atorvastatin_charite_Cohort | 0,727 | 0,606 | 0,87 | 0,00066 |
| brain | atorvastatin_charite_Cohort | 0,600 | 0,415 | 0,87 | 0,00659 |
| breast | atorvastatin_charite_Cohort | 0,540 | 0,368 | 0,79 | 0,00174 |
| cancer | atorvastatin_charite_Cohort | 0,512 | 0,480 | 0,55 | 0,00000 |
| colorectal | atorvastatin_charite_Cohort | 0,450 | 0,352 | 0,58 | 0,00000 |
| esophagus | atorvastatin_charite_Cohort | 0,304 | 0,154 | 0,60 | 0,00065 |
| kidney | atorvastatin_charite_Cohort | 0,673 | 0,528 | 0,86 | 0,00133 |
| liver | atorvastatin_charite_Cohort | 0,306 | 0,198 | 0,47 | 0,00000 |
| lung | atorvastatin_charite_Cohort | 0,619 | 0,508 | 0,75 | 0,00000 |
| ovaries | atorvastatin_charite_Cohort | 0,333 | 0,161 | 0,69 | 0,00317 |
| pancreas | atorvastatin_charite_Cohort | 0,540 | 0,393 | 0,74 | 0,00016 |
| prostate | atorvastatin_charite_Cohort | 0,491 | 0,393 | 0,61 | 0,00000 |
| seq77 | atorvastatin_charite_Cohort | 0,418 | 0,342 | 0,51 | 0,00000 |
| seq78 | atorvastatin_charite_Cohort | 0,338 | 0,275 | 0,42 | 0,00000 |
| seq79 | atorvastatin_charite_Cohort | 0,406 | 0,334 | 0,49 | 0,00000 |
| stomach | atorvastatin_charite_Cohort | 0,356 | 0,213 | 0,60 | 0,00009 |
| bladder | atorvastatin_transatlantic_Cohort | 0,575 | 0,481 | 0,69 | 0,00000 |
| brain | atorvastatin_transatlantic_Cohort | 0,506 | 0,373 | 0,69 | 0,00001 |
| breast | atorvastatin_transatlantic_Cohort | 0,410 | 0,296 | 0,57 | 0,00000 |
| cancer | atorvastatin_transatlantic_Cohort | 0,468 | 0,446 | 0,49 | 0,00000 |
| colorectal | atorvastatin_transatlantic_Cohort | 0,389 | 0,315 | 0,48 | 0,00000 |
| esophagus | atorvastatin_transatlantic_Cohort | 0,436 | 0,296 | 0,64 | 0,00003 |
| kidney | atorvastatin_transatlantic_Cohort | 0,547 | 0,431 | 0,69 | 0,00000 |
| liver | atorvastatin_transatlantic_Cohort | 0,272 | 0,184 | 0,40 | 0,00000 |
| lung | atorvastatin_transatlantic_Cohort | 0,586 | 0,500 | 0,69 | 0,00000 |
| ovaries | atorvastatin_transatlantic_Cohort | 0,287 | 0,166 | 0,50 | 0,00001 |
| pancreas | atorvastatin_transatlantic_Cohort | 0,474 | 0,361 | 0,62 | 0,00000 |
| prostate | atorvastatin_transatlantic_Cohort | 0,479 | 0,403 | 0,57 | 0,00000 |
| seq77 | atorvastatin_transatlantic_Cohort | 0,366 | 0,308 | 0,44 | 0,00000 |
| seq78 | atorvastatin_transatlantic_Cohort | 0,323 | 0,276 | 0,38 | 0,00000 |
| seq79 | atorvastatin_transatlantic_Cohort | 0,401 | 0,347 | 0,46 | 0,00000 |
| stomach | atorvastatin_transatlantic_Cohort | 0,272 | 0,164 | 0,45 | 0,00000 |
| bladder | atorvastatin_UVA_Cohort | 0,410 | 0,252 | 0,67 | 0,00036 |
| brain | atorvastatin_UVA_Cohort | 0,415 | 0,244 | 0,70 | 0,00117 |
| breast | atorvastatin_UVA_Cohort | 0,333 | 0,189 | 0,59 | 0,00016 |
| cancer | atorvastatin_UVA_Cohort | 0,449 | 0,416 | 0,48 | 0,00000 |
| colorectal | atorvastatin_UVA_Cohort | 0,412 | 0,281 | 0,61 | 0,00001 |
| esophagus | atorvastatin_UVA_Cohort | 0,724 | 0,465 | 1,13 | 0,15360 |
| kidney | atorvastatin_UVA_Cohort | 0,428 | 0,197 | 0,94 | 0,03239 |
| liver | atorvastatin_UVA_Cohort | 0,399 | 0,182 | 0,88 | 0,02183 |
| lung | atorvastatin_UVA_Cohort | 0,593 | 0,458 | 0,77 | 0,00009 |
| ovaries | atorvastatin_UVA_Cohort | 0,294 | 0,131 | 0,66 | 0,00308 |
| pancreas | atorvastatin_UVA_Cohort | 0,502 | 0,307 | 0,82 | 0,00612 |
| prostate | atorvastatin_UVA_Cohort | 0,521 | 0,397 | 0,68 | 0,00000 |
| seq77 | atorvastatin_UVA_Cohort | 0,375 | 0,275 | 0,51 | 0,00000 |
| seq78 | atorvastatin_UVA_Cohort | 0,356 | 0,282 | 0,45 | 0,00000 |
| seq79 | atorvastatin_UVA_Cohort | 0,417 | 0,337 | 0,52 | 0,00000 |
| stomach | atorvastatin_UVA_Cohort | 0,153 | 0,023 | 1,01 | 0,05112 |
| bladder | fluvastatin_charite_Cohort | 0,856 | 0,558 | 1,32 | 0,48671 |

| | | | | | |
|---|---|---|---|---|---|
| brain | fluvastatin_charite_Cohort | 0,000 | 0,000 | nan | nan |
| breast | fluvastatin_charite_Cohort | 0,472 | 0,141 | 1,58 | 0,22567 |
| cancer | fluvastatin_charite_Cohort | 0,649 | 0,559 | 0,75 | 0,00000 |
| colorectal | fluvastatin_charite_Cohort | 0,570 | 0,317 | 1,03 | 0,06003 |
| esophagus | fluvastatin_charite_Cohort | 1,000 | 0,537 | 1,86 | 1,00000 |
| kidney | fluvastatin_charite_Cohort | 1,144 | 0,811 | 1,62 | 0,45259 |
| liver | fluvastatin_charite_Cohort | 0,484 | 0,206 | 1,14 | 0,09544 |
| lung | fluvastatin_charite_Cohort | 0,591 | 0,330 | 1,06 | 0,07642 |
| ovaries | fluvastatin_charite_Cohort | 0,399 | 0,069 | 2,31 | 0,30909 |
| pancreas | fluvastatin_charite_Cohort | 0,332 | 0,094 | 1,18 | 0,08713 |
| prostate | fluvastatin_charite_Cohort | 0,726 | 0,462 | 1,14 | 0,16575 |
| seq77 | fluvastatin_charite_Cohort | 0,424 | 0,245 | 0,74 | 0,00228 |
| seq78 | fluvastatin_charite_Cohort | 0,536 | 0,361 | 0,80 | 0,00204 |
| seq79 | fluvastatin_charite_Cohort | 0,625 | 0,426 | 0,92 | 0,01616 |
| stomach | fluvastatin_charite_Cohort | 0,285 | 0,046 | 1,75 | 0,17729 |
| bladder | fluvastatin_transatlantic_Cohort | 0,872 | 0,570 | 1,34 | 0,53944 |
| brain | fluvastatin_transatlantic_Cohort | 0,000 | 0,000 | nan | nan |
| breast | fluvastatin_transatlantic_Cohort | 0,500 | 0,150 | 1,66 | 0,26136 |
| cancer | fluvastatin_transatlantic_Cohort | 0,679 | 0,587 | 0,79 | 0,00000 |
| colorectal | fluvastatin_transatlantic_Cohort | 0,666 | 0,390 | 1,14 | 0,13635 |
| esophagus | fluvastatin_transatlantic_Cohort | 1,000 | 0,537 | 1,86 | 1,00000 |
| kidney | fluvastatin_transatlantic_Cohort | 1,247 | 0,903 | 1,72 | 0,18191 |
| liver | fluvastatin_transatlantic_Cohort | 0,615 | 0,272 | 1,39 | 0,24529 |
| lung | fluvastatin_transatlantic_Cohort | 0,639 | 0,361 | 1,13 | 0,12455 |
| ovaries | fluvastatin_transatlantic_Cohort | 0,500 | 0,091 | 2,52 | 0,42092 |
| pancreas | fluvastatin_transatlantic_Cohort | 0,399 | 0,116 | 1,38 | 0,14618 |
| prostate | fluvastatin_transatlantic_Cohort | 0,705 | 0,447 | 1,11 | 0,13286 |
| seq77 | fluvastatin_transatlantic_Cohort | 0,476 | 0,277 | 0,82 | 0,00715 |
| seq78 | fluvastatin_transatlantic_Cohort | 0,594 | 0,403 | 0,88 | 0,00845 |
| seq79 | fluvastatin_transatlantic_Cohort | 0,625 | 0,426 | 0,92 | 0,01616 |
| stomach | fluvastatin_transatlantic_Cohort | 0,285 | 0,046 | 1,75 | 0,17729 |
| bladder | fluvastatin_UVA_Cohort | nan | nan | nan | nan |
| brain | fluvastatin_UVA_Cohort | 0,000 | 0,000 | nan | nan |
| breast | fluvastatin_UVA_Cohort | 0,000 | 0,000 | nan | nan |
| cancer | fluvastatin_UVA_Cohort | 0,500 | 0,092 | 2,73 | 0,43091 |
| colorectal | fluvastatin_UVA_Cohort | 2,001 | 1,898 | 2,11 | 0,00000 |
| esophagus | fluvastatin_UVA_Cohort | nan | nan | nan | nan |
| kidney | fluvastatin_UVA_Cohort | 0,000 | 0,000 | nan | nan |
| liver | fluvastatin_UVA_Cohort | 0,000 | 0,000 | nan | nan |
| lung | fluvastatin_UVA_Cohort | 0,000 | 0,000 | nan | nan |
| ovaries | fluvastatin_UVA_Cohort | 0,000 | 0,000 | nan | nan |
| pancreas | fluvastatin_UVA_Cohort | 0,000 | 0,000 | nan | nan |
| prostate | fluvastatin_UVA_Cohort | 0,000 | 0,000 | nan | nan |
| seq77 | fluvastatin_UVA_Cohort | 0,000 | 0,000 | nan | nan |
| seq78 | fluvastatin_UVA_Cohort | 0,000 | 0,000 | nan | nan |
| seq79 | fluvastatin_UVA_Cohort | 0,000 | 0,000 | nan | nan |
| stomach | fluvastatin_UVA_Cohort | 0,000 | 0,000 | nan | nan |
| bladder | pravastatin_charite_Cohort | 1,000 | 0,720 | 1,39 | 1,00000 |
| brain | pravastatin_charite_Cohort | 0,444 | 0,131 | 1,51 | 0,19388 |
| breast | pravastatin_charite_Cohort | 0,833 | 0,426 | 1,63 | 0,60544 |
| cancer | pravastatin_charite_Cohort | 0,794 | 0,709 | 0,89 | 0,00009 |
| colorectal | pravastatin_charite_Cohort | 0,393 | 0,192 | 0,80 | 0,01055 |
| esophagus | pravastatin_charite_Cohort | 0,631 | 0,248 | 1,61 | 0,34004 |

| | | | | | |
|---|---|---|---|---|---|
| kidney | pravastatin_charite_Cohort | 0,936 | 0,607 | 1,44 | 0,77709 |
| liver | pravastatin_charite_Cohort | 0,124 | 0,019 | 0,83 | 0,03007 |
| lung | pravastatin_charite_Cohort | 0,726 | 0,462 | 1,14 | 0,16575 |
| ovaries | pravastatin_charite_Cohort | 1,046 | 0,601 | 1,82 | 0,88290 |
| pancreas | pravastatin_charite_Cohort | 0,624 | 0,301 | 1,29 | 0,20578 |
| prostate | pravastatin_charite_Cohort | 0,968 | 0,721 | 1,30 | 0,83985 |
| seq77 | pravastatin_charite_Cohort | 0,726 | 0,527 | 1,00 | 0,04967 |
| seq78 | pravastatin_charite_Cohort | 0,602 | 0,424 | 0,85 | 0,00447 |
| seq79 | pravastatin_charite_Cohort | 0,579 | 0,392 | 0,86 | 0,00605 |
| stomach | pravastatin_charite_Cohort | 0,696 | 0,316 | 1,54 | 0,37531 |
| bladder | pravastatin_transatlantic_Cohort | 0,618 | 0,421 | 0,91 | 0,01404 |
| brain | pravastatin_transatlantic_Cohort | 0,509 | 0,255 | 1,02 | 0,05510 |
| breast | pravastatin_transatlantic_Cohort | 0,579 | 0,334 | 1,00 | 0,05124 |
| cancer | pravastatin_transatlantic_Cohort | 0,645 | 0,591 | 0,70 | 0,00000 |
| colorectal | pravastatin_transatlantic_Cohort | 0,373 | 0,224 | 0,62 | 0,00017 |
| esophagus | pravastatin_transatlantic_Cohort | 0,483 | 0,206 | 1,13 | 0,09414 |
| kidney | pravastatin_transatlantic_Cohort | 0,720 | 0,466 | 1,11 | 0,13937 |
| liver | pravastatin_transatlantic_Cohort | 0,137 | 0,036 | 0,52 | 0,00362 |
| lung | pravastatin_transatlantic_Cohort | 0,697 | 0,508 | 0,96 | 0,02526 |
| ovaries | pravastatin_transatlantic_Cohort | 0,856 | 0,522 | 1,41 | 0,54936 |
| pancreas | pravastatin_transatlantic_Cohort | 0,498 | 0,273 | 0,91 | 0,02278 |
| prostate | pravastatin_transatlantic_Cohort | 0,900 | 0,716 | 1,13 | 0,37260 |
| seq77 | pravastatin_transatlantic_Cohort | 0,491 | 0,354 | 0,68 | 0,00003 |
| seq78 | pravastatin_transatlantic_Cohort | 0,487 | 0,372 | 0,64 | 0,00000 |
| seq79 | pravastatin_transatlantic_Cohort | 0,475 | 0,356 | 0,64 | 0,00000 |
| stomach | pravastatin_transatlantic_Cohort | 0,443 | 0,186 | 1,05 | 0,06493 |
| bladder | pravastatin_UVA_Cohort | 0,000 | 0,000 | nan | nan |
| brain | pravastatin_UVA_Cohort | 0,568 | 0,248 | 1,30 | 0,18243 |
| breast | pravastatin_UVA_Cohort | 0,497 | 0,212 | 1,16 | 0,10739 |
| cancer | pravastatin_UVA_Cohort | 0,558 | 0,488 | 0,64 | 0,00000 |
| colorectal | pravastatin_UVA_Cohort | 0,554 | 0,280 | 1,10 | 0,08948 |
| esophagus | pravastatin_UVA_Cohort | 0,330 | 0,055 | 1,98 | 0,22732 |
| kidney | pravastatin_UVA_Cohort | 0,615 | 0,194 | 1,95 | 0,41655 |
| liver | pravastatin_UVA_Cohort | 0,396 | 0,068 | 2,30 | 0,30647 |
| lung | pravastatin_UVA_Cohort | 0,784 | 0,512 | 1,20 | 0,26634 |
| ovaries | pravastatin_UVA_Cohort | 0,519 | 0,196 | 1,38 | 0,18810 |
| pancreas | pravastatin_UVA_Cohort | 0,541 | 0,205 | 1,43 | 0,21604 |
| prostate | pravastatin_UVA_Cohort | 0,887 | 0,626 | 1,26 | 0,50939 |
| seq77 | pravastatin_UVA_Cohort | 0,165 | 0,056 | 0,49 | 0,00115 |
| seq78 | pravastatin_UVA_Cohort | 0,486 | 0,325 | 0,73 | 0,00047 |
| seq79 | pravastatin_UVA_Cohort | 0,453 | 0,298 | 0,69 | 0,00023 |
| stomach | pravastatin_UVA_Cohort | 0,000 | 0,000 | nan | nan |
| bladder | simvastatin_charite_Cohort | 0,962 | 0,892 | 1,04 | 0,31797 |
| brain | simvastatin_charite_Cohort | 0,807 | 0,692 | 0,94 | 0,00609 |
| breast | simvastatin_charite_Cohort | 0,685 | 0,583 | 0,80 | 0,00001 |
| cancer | simvastatin_charite_Cohort | 0,766 | 0,747 | 0,79 | 0,00000 |
| colorectal | simvastatin_charite_Cohort | 0,682 | 0,623 | 0,75 | 0,00000 |
| esophagus | simvastatin_charite_Cohort | 0,835 | 0,712 | 0,98 | 0,02670 |
| kidney | simvastatin_charite_Cohort | 0,909 | 0,822 | 1,00 | 0,06109 |
| liver | simvastatin_charite_Cohort | 0,593 | 0,514 | 0,68 | 0,00000 |
| lung | simvastatin_charite_Cohort | 0,887 | 0,822 | 0,96 | 0,00219 |
| ovaries | simvastatin_charite_Cohort | 0,631 | 0,504 | 0,79 | 0,00007 |
| pancreas | simvastatin_charite_Cohort | 0,728 | 0,640 | 0,83 | 0,00000 |

| | | | | | |
|---|---|---|---|---|---|
| prostate | simvastatin_charite_Cohort | 0,776 | 0,714 | 0,84 | 0,00000 |
| seq77 | simvastatin_charite_Cohort | 0,708 | 0,659 | 0,76 | 0,00000 |
| seq78 | simvastatin_charite_Cohort | 0,628 | 0,586 | 0,67 | 0,00000 |
| seq79 | simvastatin_charite_Cohort | 0,661 | 0,617 | 0,71 | 0,00000 |
| stomach | simvastatin_charite_Cohort | 0,758 | 0,654 | 0,88 | 0,00029 |
| bladder | simvastatin_transatlantic_Cohort | 1,026 | 0,956 | 1,10 | 0,49152 |
| brain | simvastatin_transatlantic_Cohort | 0,797 | 0,689 | 0,92 | 0,00240 |
| breast | simvastatin_transatlantic_Cohort | 0,705 | 0,608 | 0,82 | 0,00001 |
| cancer | simvastatin_transatlantic_Cohort | 0,780 | 0,762 | 0,80 | 0,00000 |
| colorectal | simvastatin_transatlantic_Cohort | 0,754 | 0,690 | 0,82 | 0,00000 |
| esophagus | simvastatin_transatlantic_Cohort | 0,883 | 0,759 | 1,03 | 0,10658 |
| kidney | simvastatin_transatlantic_Cohort | 1,015 | 0,922 | 1,12 | 0,77260 |
| liver | simvastatin_transatlantic_Cohort | 0,682 | 0,595 | 0,78 | 0,00000 |
| lung | simvastatin_transatlantic_Cohort | 0,942 | 0,877 | 1,01 | 0,10404 |
| ovaries | simvastatin_transatlantic_Cohort | 0,634 | 0,512 | 0,79 | 0,00003 |
| pancreas | simvastatin_transatlantic_Cohort | 0,792 | 0,700 | 0,90 | 0,00025 |
| prostate | simvastatin_transatlantic_Cohort | 0,801 | 0,740 | 0,87 | 0,00000 |
| seq77 | simvastatin_transatlantic_Cohort | 0,758 | 0,708 | 0,81 | 0,00000 |
| seq78 | simvastatin_transatlantic_Cohort | 0,653 | 0,612 | 0,70 | 0,00000 |
| seq79 | simvastatin_transatlantic_Cohort | 0,679 | 0,634 | 0,73 | 0,00000 |
| stomach | simvastatin_transatlantic_Cohort | 0,838 | 0,727 | 0,97 | 0,01471 |
| bladder | simvastatin_UVA_Cohort | 0,365 | 0,177 | 0,75 | 0,00643 |
| brain | simvastatin_UVA_Cohort | 0,715 | 0,422 | 1,21 | 0,21364 |
| breast | simvastatin_UVA_Cohort | 0,677 | 0,441 | 1,04 | 0,07366 |
| cancer | simvastatin_UVA_Cohort | 0,603 | 0,552 | 0,66 | 0,00000 |
| colorectal | simvastatin_UVA_Cohort | 0,426 | 0,246 | 0,74 | 0,00244 |
| esophagus | simvastatin_UVA_Cohort | 0,719 | 0,377 | 1,37 | 0,32152 |
| kidney | simvastatin_UVA_Cohort | 0,000 | 0,000 | nan | nan |
| liver | simvastatin_UVA_Cohort | 0,232 | 0,037 | 1,46 | 0,11928 |
| lung | simvastatin_UVA_Cohort | 0,752 | 0,557 | 1,02 | 0,06308 |
| ovaries | simvastatin_UVA_Cohort | 0,526 | 0,265 | 1,05 | 0,06660 |
| pancreas | simvastatin_UVA_Cohort | 0,549 | 0,277 | 1,09 | 0,08564 |
| prostate | simvastatin_UVA_Cohort | 0,539 | 0,362 | 0,80 | 0,00234 |
| seq77 | simvastatin_UVA_Cohort | 0,538 | 0,382 | 0,76 | 0,00042 |
| seq78 | simvastatin_UVA_Cohort | 0,498 | 0,381 | 0,65 | 0,00000 |
| seq79 | simvastatin_UVA_Cohort | 0,504 | 0,384 | 0,66 | 0,00000 |
| stomach | simvastatin_UVA_Cohort | 0,658 | 0,259 | 1,67 | 0,38583 |
| bladder | statins_charite_Cohort | 0,944 | 0,881 | 1,01 | 0,10547 |
| brain | statins_charite_Cohort | 0,741 | 0,643 | 0,86 | 0,00004 |
| breast | statins_charite_Cohort | 0,650 | 0,562 | 0,75 | 0,00000 |
| cancer | statins_charite_Cohort | 0,710 | 0,694 | 0,73 | 0,00000 |
| cancersubentities | statins_charite_Cohort | 0,728 | 0,708 | 0,75 | 0,00000 |
| colorectal | statins_charite_Cohort | 0,629 | 0,579 | 0,69 | 0,00000 |
| esophagus | statins_charite_Cohort | 0,743 | 0,637 | 0,87 | 0,00016 |
| kidney | statins_charite_Cohort | 0,901 | 0,823 | 0,99 | 0,02354 |
| liver | statins_charite_Cohort | 0,532 | 0,466 | 0,61 | 0,00000 |
| lung | statins_charite_Cohort | 0,837 | 0,779 | 0,90 | 0,00000 |
| ovaries | statins_charite_Cohort | 0,607 | 0,494 | 0,75 | 0,00000 |
| pancreas | statins_charite_Cohort | 0,675 | 0,599 | 0,76 | 0,00000 |
| prostate | statins_charite_Cohort | 0,737 | 0,683 | 0,79 | 0,00000 |
| seq77 | statins_charite_Cohort | 0,662 | 0,620 | 0,71 | 0,00000 |
| seq78 | statins_charite_Cohort | 0,578 | 0,543 | 0,62 | 0,00000 |
| seq79 | statins_charite_Cohort | 0,619 | 0,580 | 0,66 | 0,00000 |

| | | | | | |
|---|---|---|---|---|---|
| stomach | statins_charite_Cohort | 0,673 | 0,583 | 0,78 | 0,00000 |
| bladder | statins_transatlantic_Cohort | 0,910 | 0,850 | 0,97 | 0,00665 |
| brain | statins_transatlantic_Cohort | 0,698 | 0,613 | 0,80 | 0,00000 |
| breast | statins_transatlantic_Cohort | 0,610 | 0,534 | 0,70 | 0,00000 |
| cancer | statins_transatlantic_Cohort | 0,675 | 0,661 | 0,69 | 0,00000 |
| colorectal | statins_transatlantic_Cohort | 0,622 | 0,573 | 0,67 | 0,00000 |
| esophagus | statins_transatlantic_Cohort | 0,786 | 0,685 | 0,90 | 0,00061 |
| kidney | statins_transatlantic_Cohort | 0,893 | 0,817 | 0,98 | 0,01255 |
| liver | statins_transatlantic_Cohort | 0,540 | 0,474 | 0,62 | 0,00000 |
| lung | statins_transatlantic_Cohort | 0,817 | 0,764 | 0,87 | 0,00000 |
| ovaries | statins_transatlantic_Cohort | 0,547 | 0,454 | 0,66 | 0,00000 |
| pancreas | statins_transatlantic_Cohort | 0,668 | 0,596 | 0,75 | 0,00000 |
| prostate | statins_transatlantic_Cohort | 0,730 | 0,681 | 0,78 | 0,00000 |
| seq77 | statins_transatlantic_Cohort | 0,635 | 0,595 | 0,68 | 0,00000 |
| seq78 | statins_transatlantic_Cohort | 0,558 | 0,526 | 0,59 | 0,00000 |
| seq79 | statins_transatlantic_Cohort | 0,599 | 0,565 | 0,64 | 0,00000 |
| stomach | statins_transatlantic_Cohort | 0,676 | 0,587 | 0,78 | 0,00000 |
| bladder | statins_UVA_Cohort | 0,376 | 0,257 | 0,55 | 0,00000 |
| brain | statins_UVA_Cohort | 0,532 | 0,384 | 0,74 | 0,00017 |
| breast | statins_UVA_Cohort | 0,473 | 0,346 | 0,65 | 0,00000 |
| cancer | statins_UVA_Cohort | 0,522 | 0,496 | 0,55 | 0,00000 |
| cancersubentities | statins_UVA_Cohort | 0,498 | 0,461 | 0,54 | 0,00000 |
| colorectal | statins_UVA_Cohort | 0,448 | 0,339 | 0,59 | 0,00000 |
| esophagus | statins_UVA_Cohort | 0,762 | 0,552 | 1,05 | 0,09826 |
| kidney | statins_UVA_Cohort | 0,332 | 0,169 | 0,66 | 0,00147 |
| liver | statins_UVA_Cohort | 0,309 | 0,156 | 0,61 | 0,00079 |
| lung | statins_UVA_Cohort | 0,679 | 0,571 | 0,81 | 0,00002 |
| ovaries | statins_UVA_Cohort | 0,427 | 0,276 | 0,66 | 0,00015 |
| pancreas | statins_UVA_Cohort | 0,507 | 0,352 | 0,73 | 0,00029 |
| prostate | statins_UVA_Cohort | 0,611 | 0,506 | 0,74 | 0,00000 |
| seq77 | statins_UVA_Cohort | 0,409 | 0,330 | 0,51 | 0,00000 |
| seq78 | statins_UVA_Cohort | 0,432 | 0,371 | 0,51 | 0,00000 |
| seq79 | statins_UVA_Cohort | 0,475 | 0,410 | 0,55 | 0,00000 |
| stomach | statins_UVA_Cohort | 0,277 | 0,112 | 0,69 | 0,00572 |

**Supplementary Table 4**

This spreadsheet supplies the results of our analyses of co-medications prescribed with statins. The first tab "Co-medication Counts" provides a list of drugs prescribed together with statins. The total number of counts of those prescriptions is provided (in descending order) for the top 10 comedications. The second tab "Co-medication General" consists of the results (odds-ratios [ORs]) for those 10 drugs provided in tab 1. The ORs are calculated for the transatlantic cohort as well as the 1:1 matched cohort design study. Besides the ORs, p-values are also provided. Drugs with an OR < 1 are highlighted in yellow. The third tab "Exclusive Drug Calculation" consists of the analysis of previously identified drugs (tab 2) with an OR < 1. Those co-medications were analyzed separately by excluding patients taking at least two drugs identified in tab 2. These analyses were performed for the transatlantic as well as the 1:1 matched cohort. In addition, we analyzed the two most prominent statins (simvastatin and atorvastatin) separately to point out differences between statins. As can be seen in the tables, atorvastatin outperformed simvastatin with respect to the ORs in the transatlantic as well as 1:1 matched cohort

| Co-medication | | |
|---|---|---|
| | NAME | Drugs prescibed with statins |
| | STATINS | 42.682 |
| 1 | Aspirin | 26.800 |
| 2 | Metoprolol | 16.740 |
| 3 | Pantoprazole | 13.652 |
| 4 | Ramipril | 11.179 |
| 5 | Amlodipine | 7.636 |
| 6 | Torsemide | 6.512 |
| 7 | Levothyroxine sodium | 5.394 |
| 8 | hydrochlorothiazide | 5.372 |
| 9 | Acetaminophen | 5.358 |
| 10 | Furosemide | 5.198 |

## ODDS-RATIO analysis for co-medication

### TRANSATLANTIC COHORT

| Diagnose | Drug | NbDrug | NbDiag | NbDiag/Diag | AllRatio | Conf-Lower | Conf-Upper | P-Value | RelativRisk | Conf-Upper | Conf-Lower | P-Value |
|---|---|---|---|---|---|---|---|---|---|---|---|---|
| cancer | statins | 42682 | 53113 | 6426 | 0,716 | 0,7 | 0,74 | 2,1139E-108 | 0,759 | 0,777 | 0,741 | 1,2698E-101 |
| cancer | aspirin | 57943 | 53158 | 8305 | 0,661 | 0,64 | 0,68 | 1,5790E-138 | 0,71 | 0,725 | 0,694 | 8,0867E-181 |
| cancer | metoprolol | 41414 | 53338 | 7833 | 0,977 | 0,95 | 1 | 7,4983E-02 | 0,981 | 1,003 | 0,96 | 8,5809E-02 |
| cancer | pantoprazole | 79764 | 55830 | 20265 | 1,607 | 1,58 | 1,64 | 0,0000E+00 | 1,453 | 1,475 | 1,431 | 0,0000E+00 |
| cancer | ramipril | 27732 | 53726 | 5829 | 1,106 | 1,07 | 1,14 | 1,1013E-09 | 1,084 | 1,11 | 1,058 | 1,2657E-10 |
| cancer | amlodipine | 22105 | 53466 | 4545 | 1,083 | 1,05 | 1,12 | 1,7966E-06 | 1,066 | 1,095 | 1,038 | 3,7359E-06 |
| cancer | torsemide | 16416 | 53791 | 4220 | 1,453 | 1,4 | 1,51 | 1,6900E-74 | 1,336 | 1,373 | 1,301 | 1,3900E-87 |
| cancer | levothyroxine | 21138 | 53350 | 4890 | 1,267 | 1,22 | 1,31 | 1,7838E-35 | 1,205 | 1,236 | 1,174 | 1,3360E-41 |
| cancer | hydrochlorothiazide | 14207 | 53549 | 2854 | 1,037 | 0,99 | 1,08 | 1,0146E-01 | 1,03 | 1,065 | 0,995 | 8,8010E-02 |
| cancer | acetaminophen | 50920 | 53467 | 9971 | 1,04 | 1,01 | 1,07 | 7,7279E-03 | 1,032 | 1,052 | 1,012 | 1,4967E-03 |
| cancer | furosemide | 17553 | 53126 | 2789 | 0,78 | 0,75 | 0,81 | 1,3308E-33 | 0,815 | 0,844 | 0,787 | 4,6544E-28 |

### 1:1 MATCHED COHORT

| Diagnose | Drug | NbDrug | NbDiag | NbDiag/Drug | MatchedRatio | Conf-Lower | Conf-Upper | P-Value |
|---|---|---|---|---|---|---|---|---|
| cancer | statins | 17859 | 53113 | 6425 | 0,497 | 0,481 | 0,514 | 0,0000E+00 |
| cancer | aspirin | 23299 | 53158 | 8304 | 0,474 | 0,46 | 0,489 | 0,0000E+00 |
| cancer | metoprolol | 18062 | 53338 | 7833 | 0,72 | 0,697 | 0,743 | 2,3898E-80 |
| cancer | pantoprazole | 36013 | 55830 | 20264 | 1,449 | 1,413 | 1,486 | 3,3304E-160 |
| cancer | ramipril | 12719 | 53726 | 5829 | 0,824 | 0,794 | 0,855 | 6,5749E-23 |
| cancer | amlodipine | 10018 | 53466 | 4545 | 0,827 | 0,793 | 0,861 | 2,4136E-18 |
| cancer | torsemide | 8069 | 53791 | 4220 | 1,093 | 1,044 | 1,143 | 1,3400E-04 |
| cancer | levothyroxine | 9506 | 53350 | 4890 | 1,061 | 1,017 | 1,106 | 5,6948E-03 |
| cancer | hydrochlorothiazide | 6365 | 53549 | 2854 | 0,803 | 0,763 | 0,844 | 1,6515E-16 |
| cancer | acetaminophen | 19907 | 53467 | 9970 | 1,009 | 0,978 | 1,041 | 5,8563E-01 |
| cancer | furosemide | 6903 | 53126 | 2789 | 0,639 | 0,608 | 0,671 | 1,4929E-63 |

Supplementary Table 4
Odds-Ratios for co-medication

## ANALYSIS OF CO-MEDICATION

### ANALYSIS OF STATINS AS A GROUP

| | | | | | | Transatlantic Study | | | | Matched Transatlantic Study | | | |
|---|---|---|---|---|---|---|---|---|---|---|---|---|---|
| | Statins | Aspirin | Furosemide | Cancer | Drug & Cancer | Odds Ratio | Lower Bound | Upper Bound | P-Value | Odds Ratio | Lower Bound | Upper Bound | P-Value |
| Statins | Yes | No | No | 53227 | 2635 | 1,025 | 0,98 | 1,07 | 0,2734 | 0,756 | 0,718 | 0,797 | <0.0001 |
| Aspirin | No | Yes | No | 53245 | 4332 | 0,821 | 0,79 | 0,85 | <0.0001 | 0,655 | 0,629 | 0,683 | <0.0001 |
| Furosemide | No | No | Yes | 53175 | 1806 | 1,154 | 1,09 | 1,22 | <0.0001 | 1,057 | 0,988 | 1,13 | 0,1055 |

Exclusion of co-medication with positive cancer preventive effecte

### ANALYSIS OF SEPARATE STATINS

| | | | | | | | Transatlantic Study | | | | Matched Transatlantic Study | | | |
|---|---|---|---|---|---|---|---|---|---|---|---|---|---|---|
| | Simvastatin | Atorvastatin | Aspirin | Furosemide | Cancer | Drug & Cancer | Odds Ratio | Lower Bound | Upper Bound | P-Value | Odds Ratio | Lower Bound | Upper Bound | P-Value |
| Simvastatin | Yes | No | No | No | 53280 | 1984 | 1,191 | 1,13 | 1,25 | <0.0001 | 0,871 | 0,819 | 0,926 | <0.0001 |
| Atorvastatin | No | Yes | No | No | 53016 | 403 | 0,634 | 0,57 | 0,7 | <0.0001 | 0,475 | 0,422 | 0,536 | <0.0001 |
| Aspirin | No | No | Yes | No | 53245 | 4332 | 0,821 | 0,79 | 0,85 | <0.0001 | 0,655 | 0,629 | 0,683 | <0.0001 |
| Furosemide | No | No | No | Yes | 53175 | 1806 | 1,154 | 1,09 | 1,22 | <0.0001 | 1,057 | 0,988 | 1,13 | 0,1055 |

Exclusion of co-medication with positive cancer preventive effecte

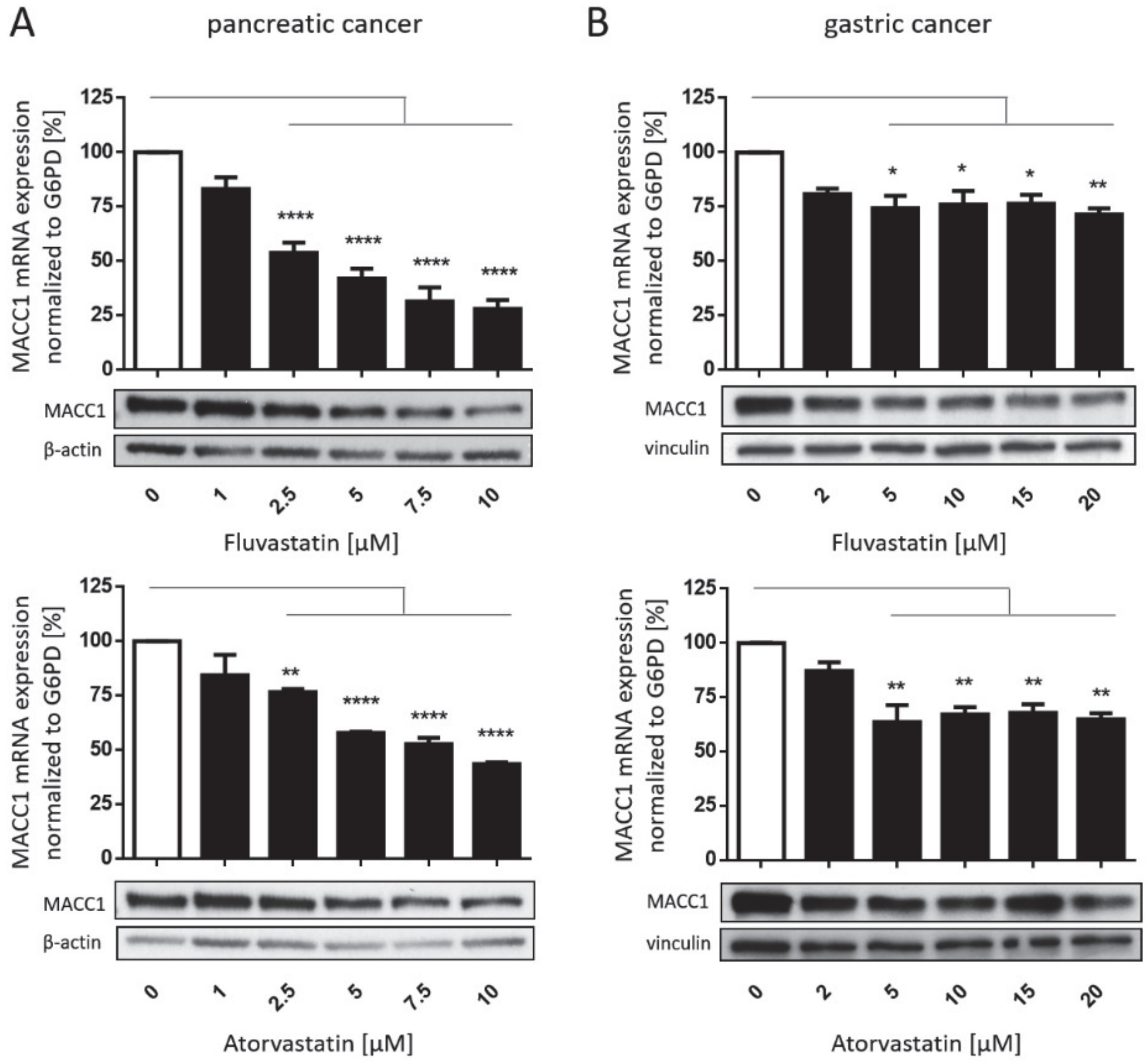


**Supplementary Figure 1.**

**Statins reduce MACC1 expression in different cancer entities.**

Fluvastatin and atorvastatin decreased MACC1 mRNA and protein expression in pancreatic cancer (BxPC3; Panel A) and gastric cancer (MKN45; Panel B) cells. MACC1 mRNA levels were normalized to G6PD mRNA expression and respective treatment controls (DMSO, indicated with white bars). Results for mRNA represent means + SEMs of three independent experiments, and for WB one representative example of at least two independent experiments is shown. In the WB, β-actin or vinculin served as loading control. Significant results were determined by one-way ANOVA and Dunnett's multiple comparison test with a confidence interval of 95% (* = $p < 0.05$, ** = $p < 0.01$, *** = $p < 0.001$, **** = $p < 0.0001$).

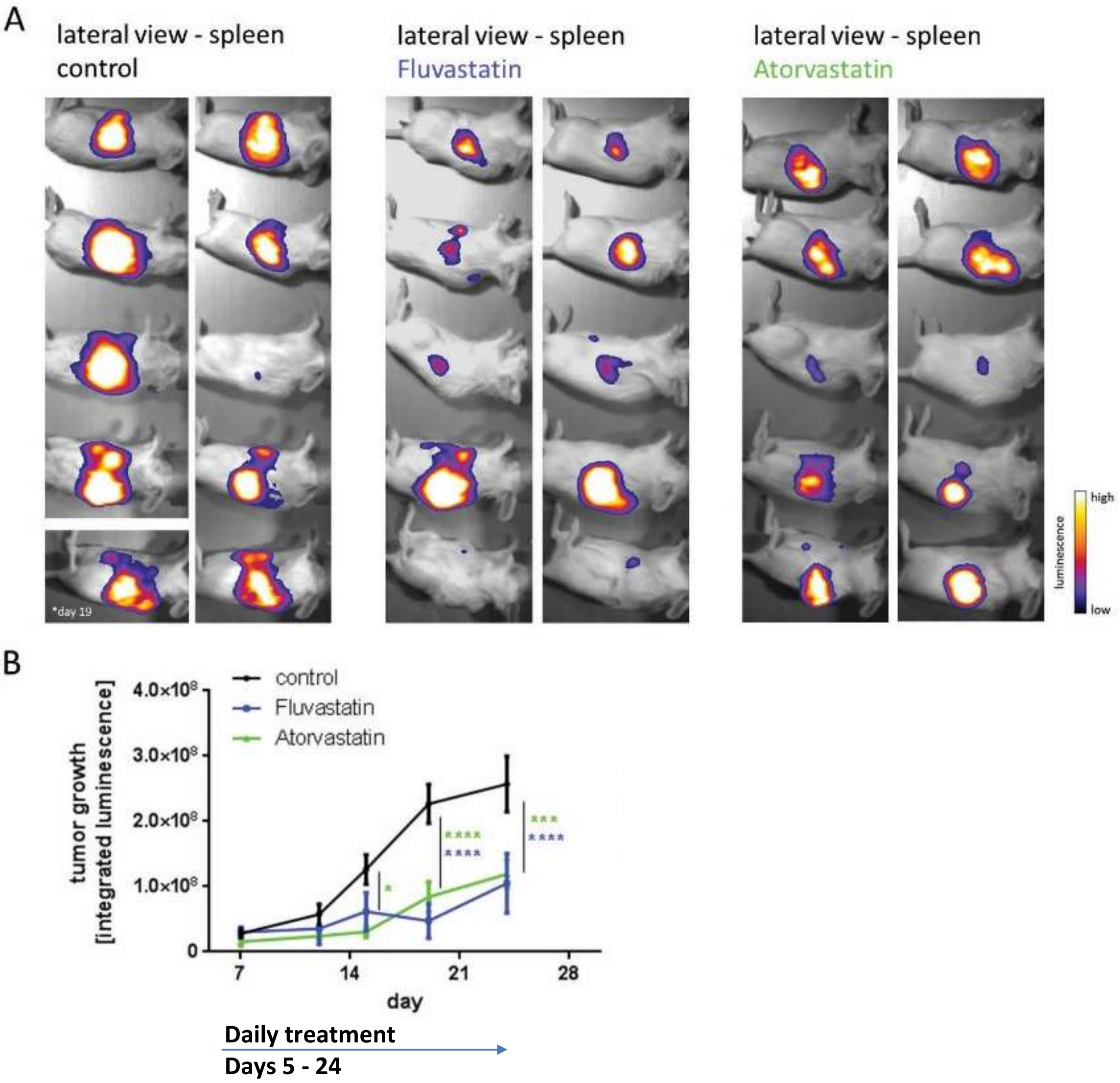


**Supplementary Figure 2.**

**Statin treatment decreases tumor burden in vivo.**

Intra-splenically xenografted (HCT116/CMVp-Luc cells) SCID-beige mice were treated either with solvent or daily doses of 13 mg/kg body weight fluvastatin or atorvastatin as indicated. Bioluminescent imaging of animals from a lateral view (A), at day 24 of statin treatment, showed significantly weaker signals, indicating restricted tumor burden in the spleen. Bioluminescent signals from all ten animals over the time course of the experiment were quantified (B). Results represent means ± SEM and significant results were determined by two-way ANOVA and Dunnet's multiple comparison test with a 95% CI (* = $p < 0.05$, ** = $p < 0.01$, *** = $p < 0.001$, **** = $p < 0.0001$). Data were analyzed by ANOVA and Dunnett's multiple comparison post tests or two-way ANOVA including Tukey's additivity test.

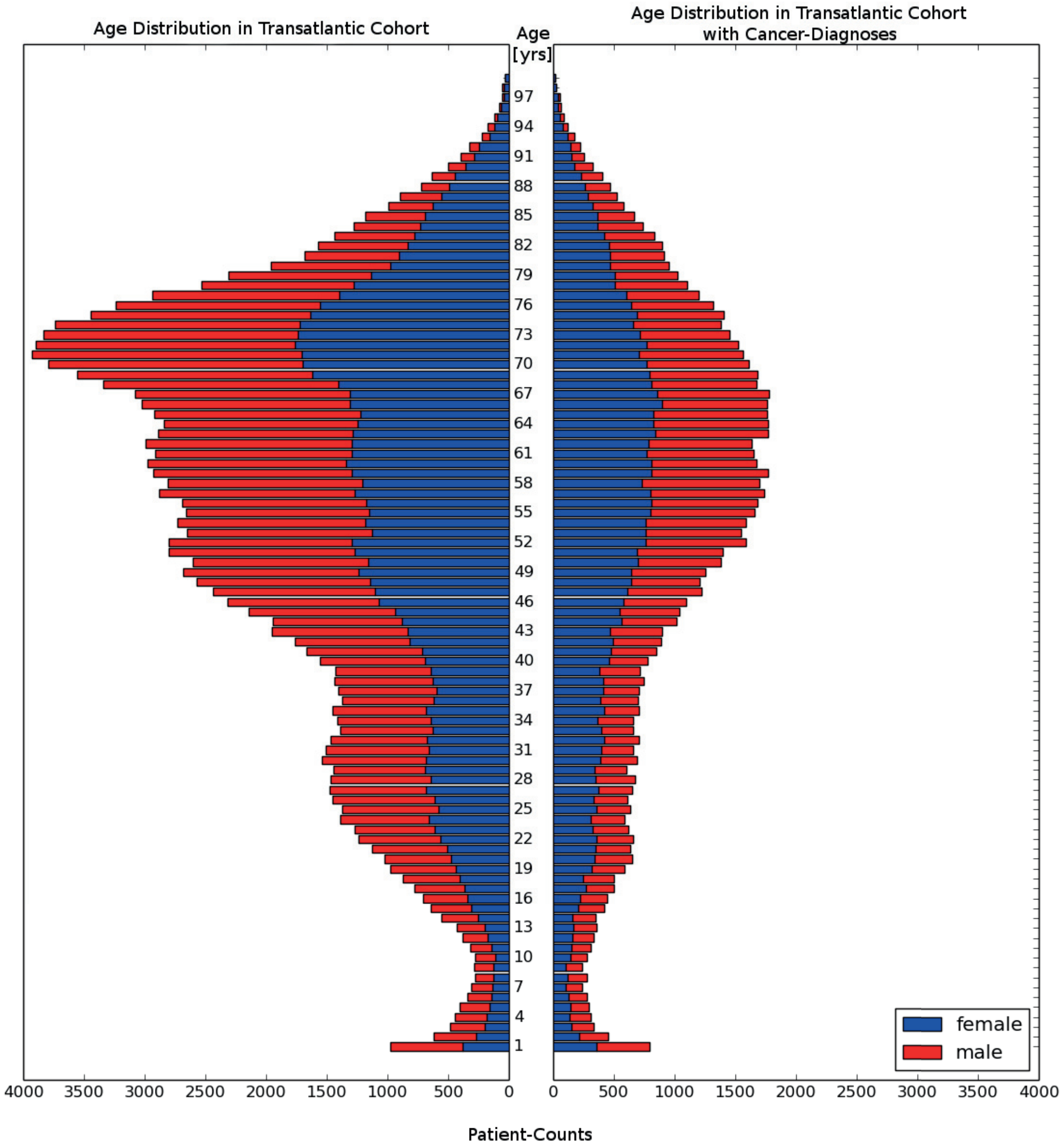


**Supplementary Figure 3.**

**Age and gender distribution in the trans-Atlantic cohort and patients diagnosed with cancer.**

The age distribution for both genders in the full trans-Atlantic cohort is shown (left half), along with the analogous distribution for patients diagnosed with cancer (right); any effects that might stem from the visible differences between these two groups were mitigated by using a 1:1 matched study design.

# Transatlantic Cohort

| Cancer Entities | Number Cancer cases | Cancer cases with statins | Cancer cases with atorvastatin | OR (95% CI) All Statins | OR (95% CI) Atorvastatin | P-Value |
|---|---|---|---|---|---|---|
| Bladder | 2480 | 570 | 88 | 1.59 (1.45-1.75) | 0.67 (0.54-0.83) | <0.001 |
| Brain | 1823 | 154 | 31 | 0.49 (0.41-0.58) | 0.32 (0.22-0.45) | <0.001 |
| Breast | 2067 | 158 | 29 | 0.44 (0.37-0.52) | 0.26 (0.18-0.37) | <0.001 |
| Colorectal | 3803 | 425 | 71 | 0.67 (0.6-0.74) | 0.34 (0.27-0.43) | <0.001 |
| Esophagus | 886 | 140 | 20 | 1.0 (0.83-1.19) | 0.43 (0.27-0.67) | 0.976 [<0.001] |
| Gastric | 1176 | 136 | 13 | 0.69 (0.58-0.83) | 0.2 (0.12-0.35) | <0.001 |
| Kidney | 1688 | 325 | 50 | 1.27 (1.12-1.43) | 0.55 (0.41-0.73) | <0.001 |
| liver | 1814 | 169 | 22 | 0.54 (0.46-0.64) | 0.22 (0.15-0.34) | <0.001 |
| Lung | 3449 | 596 | 110 | 1.11 (1.02-1.22) | 0.6 (0.5-0.73) | 0.018 [<0.001] |
| Ovaries | 1225 | 82 | 11 | 0.38 (0.3-0.48) | 0.17 (0.09-0.3) | <0.001 |
| Pancreas | 1774 | 213 | 40 | 0.72 (0.63-0.84) | 0.42 (0.31-0.58) | <0.001 |
| Prostate | 3151 | 573 | 98 | 1.18 (1.08-1.3) | 0.58 (0.47-0.71) | <0.001 |
| Sec. Cancers | 16799 | 1705 | 285 | 0.6 (0.57-0.63) | 0.31 (0.27-0.34) | <0.001 |
| Other Cancers | 27067 | 2732 | 605 | 0.59 (0.57-0.62) | 0.41 (0.37-0.44) | <0.001 |
| **Cancer Patients** | **53113** | **6425** | | **0.72 (0.7-0.74)** | | **<0.001** |
| Atorvastatin | | 1235 | | 0.41 (0.38 - 0.43) | | <0.001 |
| Fluvastatin | | 119 | | 0.7 (0.57 - 0.85) | | <0.001 |
| Lovastatin | | 61 | | 0.69 (0.53 - 0.91) | | <0.007 |
| Pravastatin | | 331 | | 0.63 (0.56 - 0.71) | | <0.001 |
| Rosuvastatin | | 147 | | 0.43 (0.36 - 0.51) | | <0.001 |
| Simvastatin | | 4609 | | 0.9 (0.87 - 0.94) | | <0.001 |

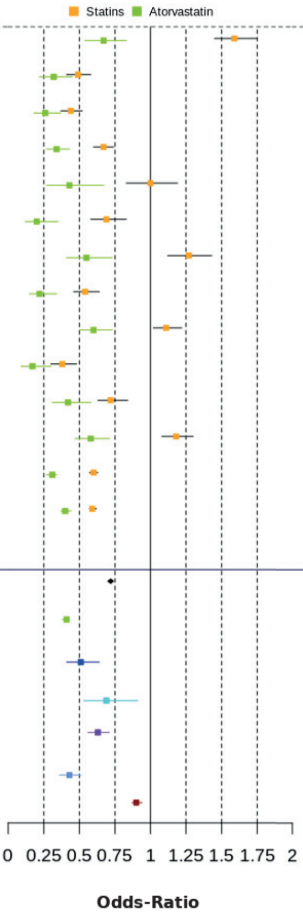


Simvastatin Pravastatin Fluvastatin Lovastatin Rosuvastatin

**Supplementary Figure 4.**

Cancer preventive effect of different statins and different cancers across the transatlantic cohort. The cancer preventive effect of statins as a group, and atorvastatin alone, were calculated for a number of different cancer types. A detailed overview of cancer incidences, cancer diagnoses and prescribed statins is provided. The cancer preventive effect is calculated as an odds-ratios for both, statins and atorvastatin. The 95% confidence intervals p-values are provided. P-values in square brackets are provided for atorvastatin, for any values that differ from the statins at large. An overview of results for all statins prescribed in the study population is presented in the bottom of the figure.

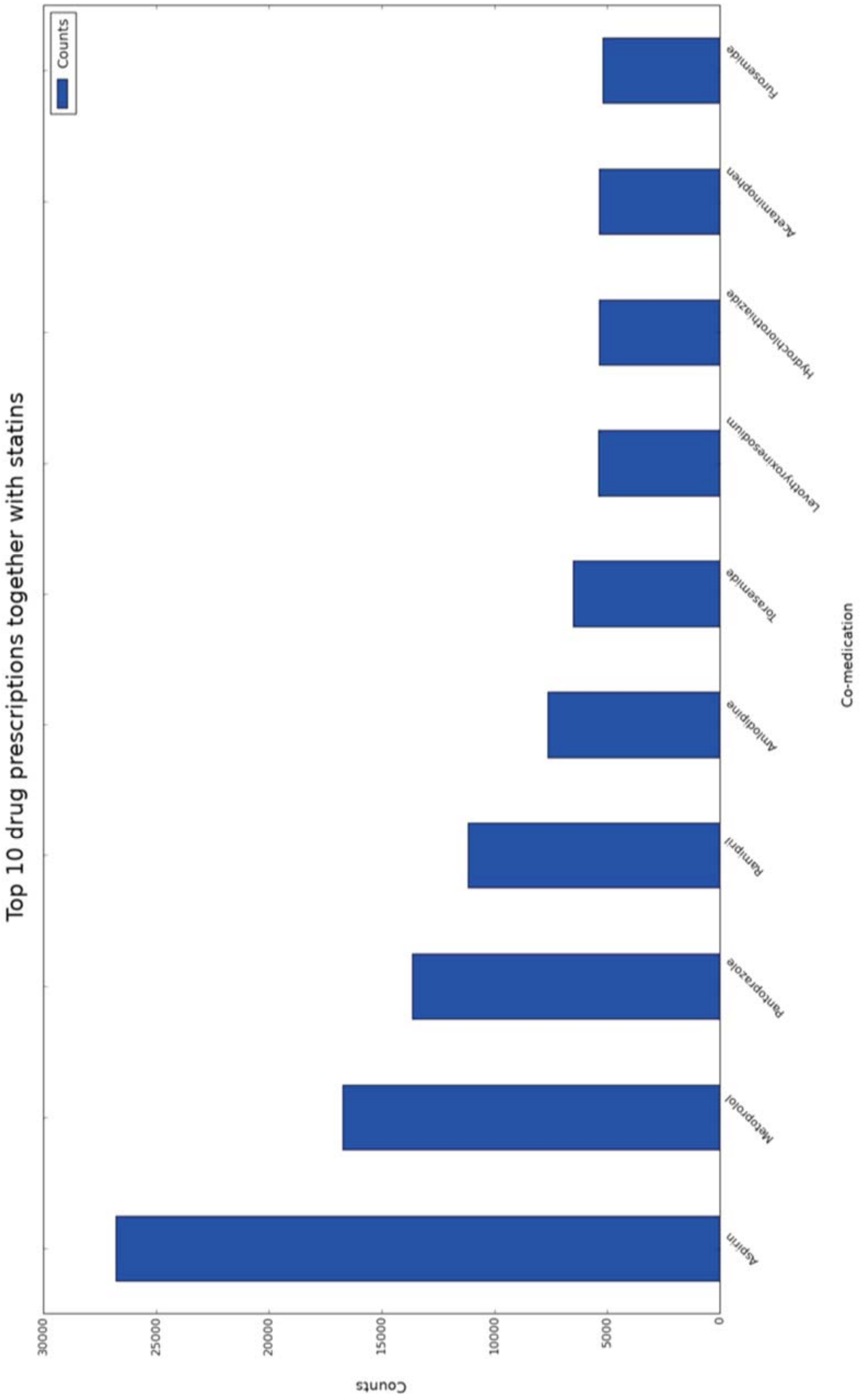


**Supplementary Figure 5.**

Co-medication with statins. The medications prescribed alongside statins are shown, in decreasing order of the total number of prescriptions of each
